# Catalyst-free synthesis of tetrahydropyrimidines *via* formal [3+3]-cycloaddition of imines with 1,3,5-hexahydro-1,3,5-triazines†

**DOI:** 10.1039/c7ra11973a

**Published:** 2018-02-01

**Authors:** Long Chen, Kai Liu, Jiangtao Sun

**Affiliations:** Jiangsu Key Laboratory of Advanced Catalytic Materials & Technology, School of Petrochemical Engineering, Changzhou University Changzhou 213164 P. R. China jtsun@cczu.edu.cn jtsun08@gmail.com

## Abstract

A practical and environmentally benign synthesis of poly-substituted tetrahydropyrimidines from readily available starting materials has been developed. This process features an unprecedented intermolecular formal [3+3]-annulation of imines and 1,3,5-hexahydro-1,3,5-triazines under catalyst-free conditions. Importantly, differing from previous transformations, the 1,3,5-triazines are firstly utilized as formal 1,3-dipoles in cycloaddition reactions.

## Abstract 2

A practical and environmentally benign synthesis of poly-substituted tetrahydropyrimidines *via* formal [3+3]-annulation of imines and 1,3,5-hexahydro-1,3,5-triazines under catalyst-free conditions has been developed.

## Graphical abstract

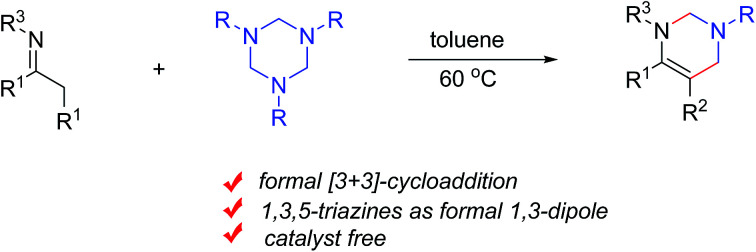

Tetrahydropyrimidines are important heterocycles which have been widely explored in various biologically active molecules and advanced materials, possessing unique properties such as antiviral activity, anti-inflammatory, muscarinic agonist activity, and sensitivity to protein–DNA interactions.^1^ However, efficient methods for tetrahydropyrimidine synthesis are rare^2^ and some of them suffer from poor practicability with low yields, harsh reaction conditions and lack readily available starting materials. Therefore, to develop simple but efficient methodologies for the direct synthesis of polysubstituted tetrahydropyrimidines is highly demanded.

As stable and readily available intermediates, 1,3,5-trisubstituted-hexahydro-1,3,5-triazines (simply as 1,3,5-triazines in this text) have been previously utilized as imine equivalents in various Lewis acid promoted [*n*+2]-cycloaddition reactions (Scheme 1a).^3^ They also have been employed as precursors of *N*-aryl formaldimines in hydroaminomethylation of π-unsaturated reactants pioneered by the Krische group,^4^ and as suitable reagents in asymmetric Mannich reaction subsequently reported by the groups of Feng^5^ and Kang^6^ (Scheme 1b). Recently, inspired by Krische's work, we found that 1,3,5-triazines could be utilized as formal 1,4-dipoles in gold-catalyzed formal [4+1] and [4+3]-cycloaddition reactions to synthesize five- and seven N-heterocycles.^7*a*^ Afterwards, we have successfully developed a series of 1,3,5-triazines involved [2+2+2],^7*b*^ [2+1+2]^7*c*,*d*^ and formal [4+3] annulations^7*e*^ (Scheme 1c). Concurrently, the groups of Hashmi^8^ and Xu^9^ also described the gold-catalyzed cycloaddition of 1,3,5-triazines with activated alkynes to produce six- and seven-membered heterocycles. Just recently, Werz and co-worker reported an elegant formal [4+3]-cycloaddition of 1,3,5-triazines with donor–acceptor cyclopropanes to prepare seven-membered ring system.^10^ Although the great advances, the former reports focused on the use of 1,3,5-triazines as formal 1,2- or 1,4-dipoles. In continuation with our ongoing interest, we want to report here the first use of 1,3,5-triazines as formal 1,3-dipoles to react with imines, providing tetrahydropyrimidines in moderate to excellent yields under catalyst-free reaction conditions.

**Scheme 1:**
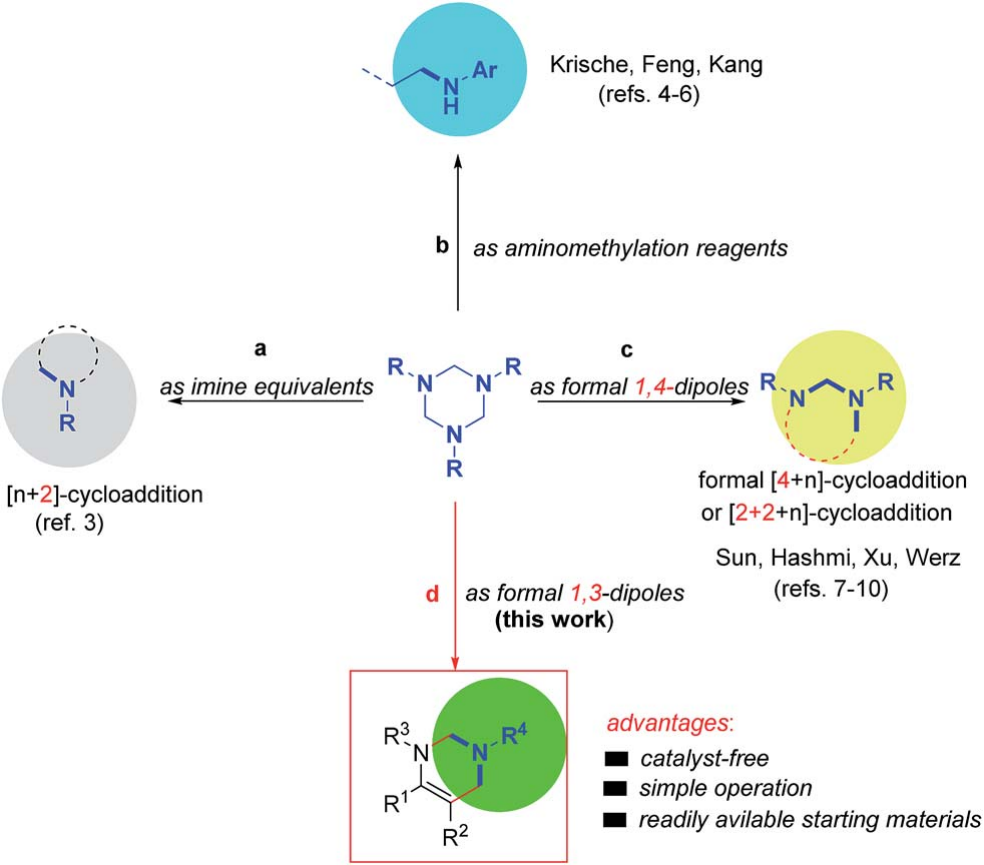
Previous reports and our protocol.

Initially, the reaction of imine 1a and 1,3,5-triazine 2a was performed in various solvents at room temperature (Table 1). All of the solvents examined such as toluene, dichloromethane (CH_2_Cl_2_), chloroform (CHCl_3_), 1,2-dichloroethane (DCE), tetrahydrofuran (THF), acetonitrile (MeCN), 1,4-dioxane, methanol, ethanol, *N*,*N*-dimethylformamide (DMF), dimethyl sulfoxide (DMSO), were all amenable to this reaction, providing tetrahydropyrimidine 3a in moderate to high yields with good to excellent conversion in 10 hours, whereas toluene proved to be the best one (entry 1). The reaction temperature was then evaluated. When the reaction was performed at 40 °C in toluene, shorter reaction time (7 h) and same yield (84%) were observed (entry 12). Furthermore, the reaction was finished in 6 hours at 60 °C or 80 °C and furnished 3a in 86% yield (entries 13 and 14).

**Table 1:** Selected optimization^a^

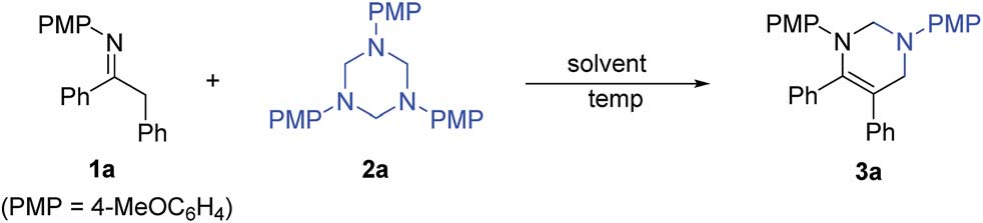					
Entry	Solvent	Temp (°C)	Time (h)	Conv.^b^ (%)	Yield^c^ (%)
1	Toluene	rt	10	100	84
2	CH_2_Cl_2_	rt	10	94	79
3	CHCl_3_	rt	10	92	78
4	DCE	rt	10	98	80
5	THF	rt	10	100	81
6	MeCN	rt	10	90	78
7	1,4-Dioxane	rt	10	93	77
8	MeOH	rt	10	92	78
9	EtOH	rt	10	87	67
10	DMF	rt	10	78	60
11	DMSO	rt	10	81	64
12	Toluene	40	7	100	84
13	Toluene	60	6	100	86
14	Toluene	80	6	100	86

a Reaction conditions: 1a (0.3 mmol), 2a (0.33 mmol), solvent (6 mL).

b Determined by GC analysis.

c Isolated yields.

With the optimal conditions in hand, we started to examine the scope of substrates (Table 2). The reaction of 1,3,5-triazines with an array of imines was carried out at 60 °C in toluene. In the beginning, the variation of R^1^ and R^2^ groups of imines has been evaluated. For *N*-4-methoxy-substituted imines, the aryl R^1^ and R^2^ groups bearing electron-donating and electron-withdrawing groups were tolerated, providing the corresponding products (3a–3f) in moderate to good yields. However, longer reaction was required for the imines bearing electron-deficient aryl groups (3c–3e), whereas *ortho*-methyl substituted substrate gave the desired product 3f in 61% yield. Varying R^2^ from aryl to alkyl groups furnished 3g (11 h) and 3h (18 h) in 72% and 58% yields, respectively. The carboxylic ester group of R^2^ was also amenable to the reaction, and tetrahydropyrimidine 3i was isolated in 74% yield after 16 h. The reaction of cyclic imine with 2a afforded 3j in 89% yield within 8 h. The molecular structure of 3j was characterized by X-ray diffraction.^11^

**Table 2:** Substrate scope^a^^,^^b^

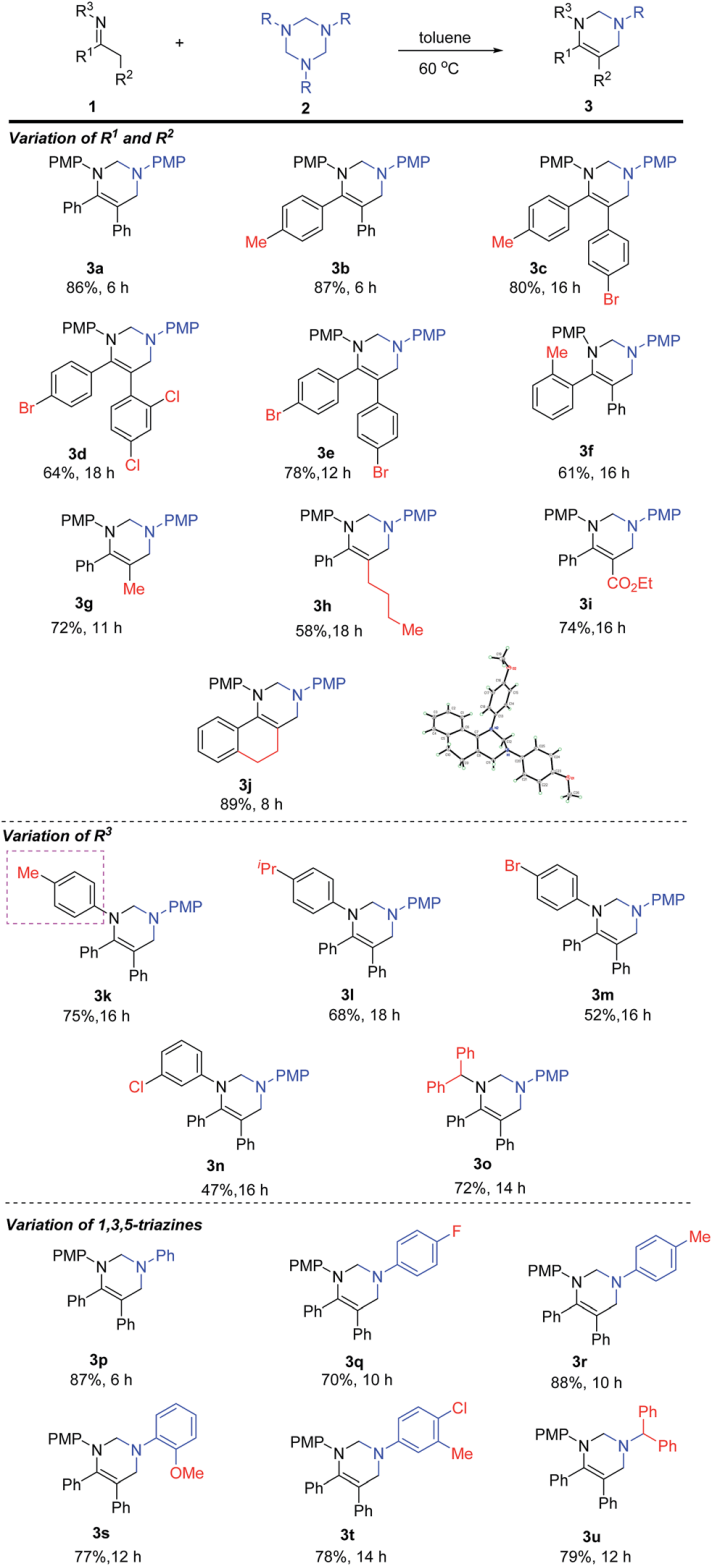

a Reaction conditions: all reactions were performed with 1 (0.3 mmol), 2 (0.33 mmol), in toluene (6 mL) and stirred at 60 °C for 6–18 h.

b Isolated yields.

Next, the scope of *N*-substituent of imines was examined. It was observed that *N*-aryl imines with electron-donating substituents gave the corresponding products in higher yields than the ones with electron-withdrawing groups (3k, 3l*vs.*3m–3n). The reaction was also applicable to *N*-alkyl imine and the desired product 3o was isolated in 72% yield. Finally, the scope of 1,3,5-triazines was evaluated. *N*-aryl-1,3,5-triazines bearing both electron-donating and electron-withdrawing groups were tolerated and the desired products were obtained in moderate to excellent yields (3p to 3t). It should be noted that *N*-benzhydryl triazine was also tolerated, providing 3u in 79% yield.

To examine the practicability of this protocol, a gram scale reaction was performed (Scheme 2). Treatment of 5 g of 1a (16.6 mmol, 1 equiv.) and 1,3,5-tribenzhydryl-1,3,5-triazine (10.7 g, 18.2 mmol, 1.1 equiv.) at 60 °C in toluene for 24 h gave 6.1 g of 3u (72% yield) as white solid after recrystallization.

**Scheme 2:**
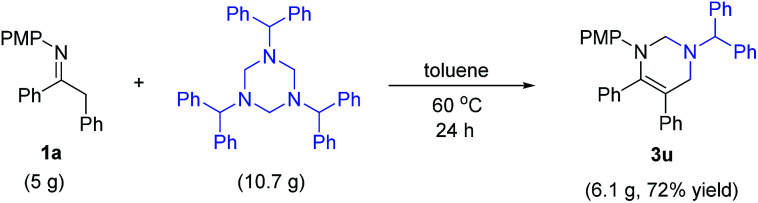
Large scale reaction.

Mechanistic studies were conducted to understand the reaction mechanism (Scheme 3). First, the reaction of 1a with aniline and paraformaldehyde in toluene at 60 °C for 12 h delivered 3p, 3a and 4 in 42%, 19% and 9% yields, respectively, indicating the exchange of imine with aniline occurred (Scheme 3a). Next, the reaction of 1a and 2a in the presence of 1 equiv. of aniline provided 78% yield of 3a and 7% yield of 3p, providing evidence for the exchange between aniline and 1,3,5-triazine (Scheme 3b). Moreover, treatment of 1a with D-2a in the presence of paraformaldehyde produces D-3a in 80% yield with 35% and 41% of hydrogen incorporation, indicating the decomposition of 1,3,5-triazines to formaldimines and further decomposed to aniline and formaldehyde (Scheme 3c).

**Scheme 3:**
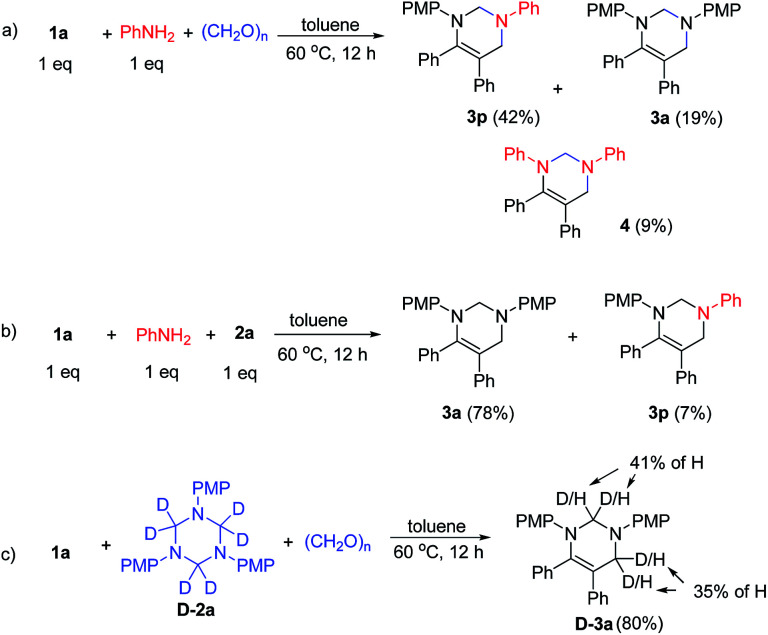
Mechanistic studies.

Based on the above results, a possible mechanism for the catalyst-free formal [3+3]-cycloaddition is proposed in Scheme 4. The dissolution of 1,3,5-triazine 2a in solvent would generate formaldimine 2a′, which can further decompose to 4-methoxyaniline and formaldehyde due to the existence of small amount of water in the solvent. Imine 1a can isomerize to enamine 1a′ in the reaction system. The whole process might be triggered by a formal aza-ene type reaction^12,1*j*^ between enamine 1a′ and formaldimine 2a′, generating active intermediates β-aminoimine IA or 1,3-diamine IB. These intermediates then react with *in situ* formed formaldehyde to generate the condensation product tetrahydropyrimidine 3a and release one molecular of water into the reaction system. The role of water and the existence of formaldehyde can be confirmed by the crossover experiments (Scheme 3).

**Scheme 4:**
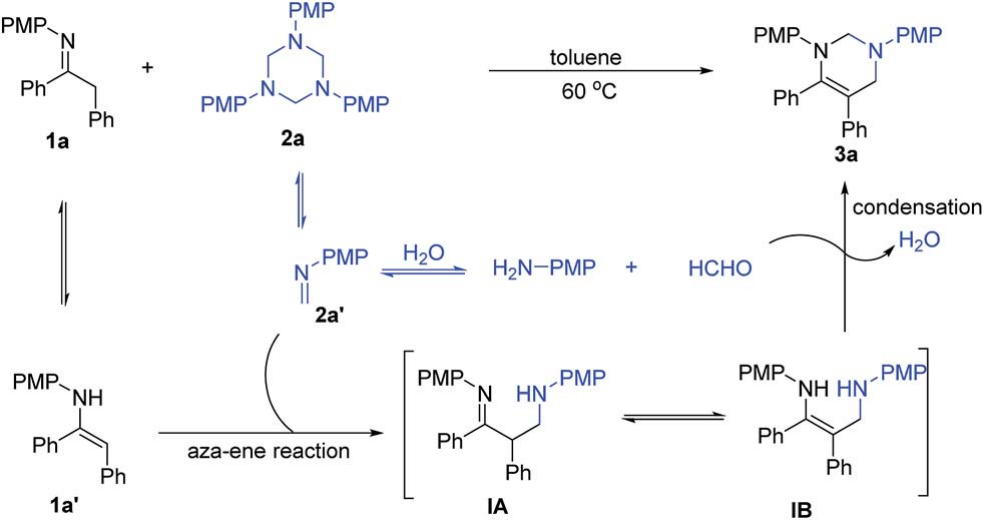
Plausible mechanism.

In summary, we have developed a novel protocol to synthesize poly-substituted tetrahydropyrimidines from readily available starting materials under catalyst-free conditions. The process is simple, practical and environmentally benign, proceeding *via* a formal [3+3]-cycloaddition between imines and 1,3,5-triazines. Typically, the 1,3,5-triazines have been firstly utilized as formal 1,3-dipoles in the cycloaddition reactions, which is unprecedented. Mechanistic studies show that this reaction is a step-wise process, namely the initial reaction of imines with *in situ* generated formaldimines, followed by condensation with formaldehyde to give the final product.

## Conflicts of interest

There are no conflicts to declare.

## Supplementary Material

RA-008-C7RA11973A-s001

RA-008-C7RA11973A-s002
