# Supplementary material for: Catalyst-free synthesis of tetrahydropyrimidines via formal [3+3]-cycloaddition of imines with 1,3,5-hexahydro-1,3,5-triazines
Source: RSC Adv. 2018 Feb 1;8(10):5532–5. doi: 10.1039/c7ra11973a (PMC9078107; doi:10.1039/c7ra11973a)
Supplement: RA-008-C7RA11973A-s001 [file RA-008-C7RA11973A-s001.pdf]

Electronic Supplementary Material (ESI) for RSC Advances.
This journal is © The Royal Society of Chemistry 2017

Catalyst-Free Synthesis of Tetrahydropyrimidines via Formal [3+3]-Cycloaddition of Imines with 1,3,5-Hexahydro-1,3,5-Triazines

Long Chen, Kai Liu and Jiangtao Sun*

Jiangsu Key Laboratory of Advanced Catalytic Materials & Technology, School of Petrochemical Engineering, Changzhou University

1 Gehu Road, 213164 Changzhou, China

E-mail: jtsun08@gmail.com or jtsun@cczu.edu.cn

Table of Contents

1. General information

2. Preparation of substrates

3 General procedure for Table 2

4. Large scale reaction for Scheme 2

5. Mechanistic studies for Scheme 3

6. X-ray structure of **3j**

7. References

8. ^{1}H NMR and ^{13}C NMR Spectra of compounds

General information

All of the reactions were performed in flame-dried tubes. For column chromatography, 200-300 mesh silica gel was used. ^1H NMR were recorded on Bruker 300 MHz, 400 MHz spectrometer and ^{13}C NMR were recorded on Bruker 75 MHz, 100 MHz or 125MHz spectrometer in $CDCl_3$. HRMS were performed on Agilent 6540 Q-TOF mass spectrometer (ESI). Melting points were determined on a SGW X-4B melting point apparatus. Triazines **2** were prepared according to previously reported literature.[1]

Preparation of substrates

Imines **1** were prepared according to the follow procedure. [2, 3]

Procedure A:

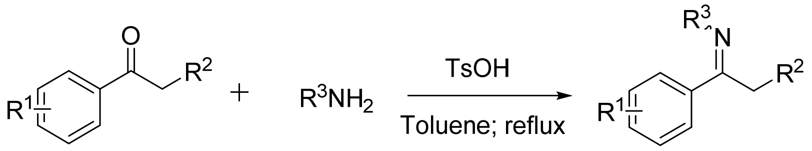

1

Ketone (10 mmol, 1 eq), R^3NH_2 (20 mmol, 2 eq), TsOH (1 mmol, 0.1 eq) and toluene (80 mL) were added into a round-bottom flask, the mixture was heated to reflux overnight under Dean-Stark conditions. The reaction mixture was cooled to room temperature. After evaporating the solvents under reduced pressure, the residue was purified by flash chromatography on silica gel (petroleum ether/ethyl acetate/Et_3N=100:1:2) or crystallization from petroleum ether to afford the imine **1**, which was unstable and used directly to next step.

Procedure B:

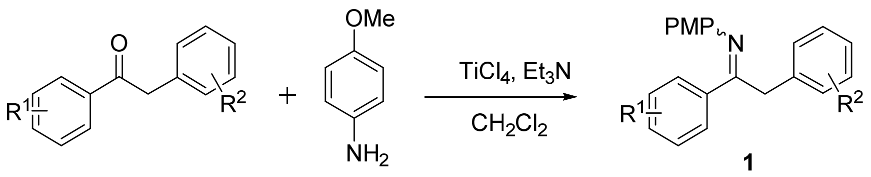

To a solution of 4-Methoxyaniline (27 mmol) and Et_3N (27 mmol) in CH_2Cl_2 (70 mL)

was added dropwise $TiCl_4$ (1 mL, 9 mmol) at 0 °C under argon atmosphere. After stirring for 30 min, the reaction mixture was allowed to warm to room temperature and a solution of ketone (9 mmol) in CH_2Cl_2 (30 mL) was added dropwisely. The reaction mixture was stirred at 40 °C overnight. The reaction mixture was filtered and washed with EtOAc, the filtrate was concentrated under vacuum to give crude product. The crude product was purified by recrystallization to give imine **1,** which was unstable and used directly to next step.

General procedure for Table 2

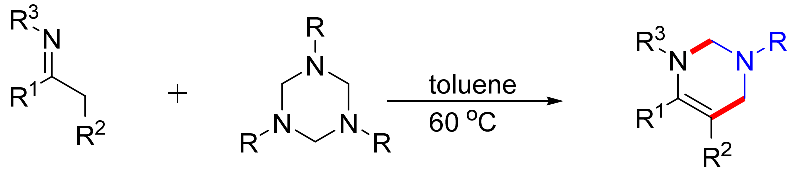

1 **2** **3**

To a tube was added **1** (0.3 mmol, 1 eq), **2** (0.33 mmol, 1.1 eq) and toluene (6 mL), the resulting mixture was stirred at 60 °C for 6-18 h. The reaction solution was concentrated and purified by flash chromatography on silica gel (petroleum ether/ethyl acetate = 80:1-20:1) to give **3**.

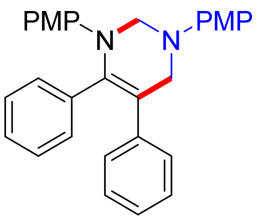

1,3-bis(4-methoxyphenyl)-5,6-diphenyl-1,2,3,4-tetrahydropyrimidine (3a):

This compound was prepared via general procedure as yellow oil (116 mg, yield: 86%).

^{1}H NMR (400 MHz, $CDCl_3$) δ 7.20-7.12 (m, 7H), 7.00-6.96 (m, 3H), 6.94-6.90 (m, 2H), 6.77 (s, 4H), 6.66-6.62 (m, 2H), 4.81 (s, 2H), 4.13 (s, 2H), 3.73 (s, 3H), 3.66 (s, 3H). ^{13}C NMR (100 MHz, $CDCl_3$) δ 155.52, 153.58, 142.50, 141.14, 140.78, 140.17, 136.84, 130.91, 129.81, 127.99, 127.49, 127.07, 126.08, 125.91, 120.11, 117.68,

114.56, 113.83, 72.65, 55.67, 55.27, 53.07. HRMS (ESI) calcd. for $C_{30}H_{29}N_2O_2$ $[M+H]^+$: 449.2224, found: 449.2221.

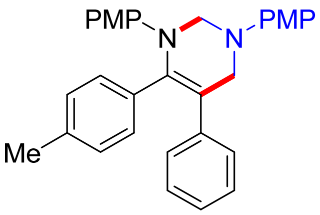

1,3-bis(4-methoxyphenyl)-5-phenyl-6-(p-tolyl)-1,2,3,4-tetrahydropyrimidine (3b):

This compound was prepared via general procedure as yellow oil (121 mg, yield: 87%).

^{1}H NMR (400 MHz, $CDCl_3$) δ 7.20-7.10 (m, 5H), 7.04 (d, J = 8.0 Hz, 2H), 6.96-6.89 (m, 2H), 6.80-6.72 (m, 6H), 6.67-6.60 (m, 2H), 4.79 (s, 2H), 4.12 (s, 2H), 3.71 (s, 3H), 3.65 (s, 3H), 2.14 (s, 3H). ^{13}C NMR (100 MHz, $CDCl_3$) δ 155.48, 153.54, 142.56, 141.33, 140.77, 140.42, 136.71, 133.86, 130.75, 129.79, 128.31, 128.00, 125.97, 125.91, 119.64, 117.61, 114.57, 113.83, 72.63, 55.67, 55.27, 53.09, 21.21. HRMS (ESI) calcd. for $C_{31}H_{30}N_2NaO_2$ $[M+Na]^+$: 485.2199, found: 485.2194.

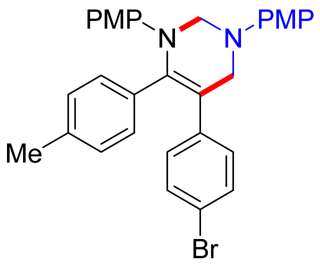

5-(4-bromophenyl)-1,3-bis(4-methoxyphenyl)-6-(p-tolyl)-1,2,3,4-tetrahydropyrimidine (3c):

This compound was prepared via general procedure as yellow oil (130 mg, yield: 80%).

^{1}H NMR (400 MHz, $CDCl_3$) δ 7.28 (d, J = 8.0 Hz, 2H), 7.01 (t, J = 8.0 Hz, 4H), 6.89 (d, J = 8.0 Hz, 2H), 6.81 (d, J = 8.0 Hz, 2H), 6.75 (s, 4H), 6.63 (d, J = 8.0 Hz, 2H), 4.77 (s, 2H), 4.08 (s, 2H), 3.72 (s, 3H), 3.66 (s, 3H), 2.16 (s, 3H). ^{13}C NMR (100 MHz, $CDCl_3$) δ 155.57, 153.63, 142.40, 141.53, 141.03, 139.37, 137.07, 133.43, 131.30,

131.05, 130.76, 128.50, 126.02, 119.64, 117.84, 117.71, 114.55, 113.82, 72.53, 55.66, 55.26, 52.80, 21.23. HRMS (ESI) calcd. for $C_{31}H_{30}BrN_2O_2$ $[M+H]^+$: 541.1485, found: 541.1484.

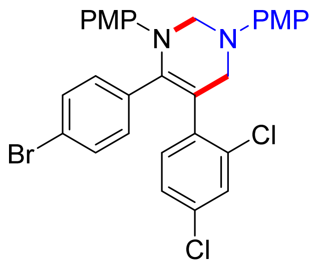

6-(4-bromophenyl)-5-(2,4-dichlorophenyl)-1,3-bis(4-methoxyphenyl)-1,2,3,4-tetrahydropyrimidine (3d):

This compound was prepared via general procedure as a white solid (114 mg, yield: 64%), mp: 99-101 °C.

^{1}H NMR (400 MHz, $CDCl_3$) δ 7.36 (d, J = 4.0 Hz, 1H), 7.15-7.09 (m, 4H), 7.02 (d, J = 8.0 Hz, 2H), 6.93 (d, J = 8.0 Hz, 2H), 6.75 (s, 4H), 6.66 (d, J = 8.0 Hz, 2H), 4.87 (d, J = 12.0 Hz, 1H), 4.77 (d, J = 12.0 Hz, 1H), 4.19 (d, J = 16.0 Hz, 1H), 3.82 (d, J = 16.0 Hz, 1H), 3.72 (s, 3H), 3.69 (s, 3H). ^{13}C NMR (100 MHz, $CDCl_3$) δ 155.86, 153.78, 142.20, 141.32, 140.71, 137.33, 135.71, 135.41, 133.50, 133.44, 131.28, 130.85, 129.53, 127.30, 125.91, 121.56, 118.18, 118.03, 114.51, 114.02, 72.81, 55.64, 55.30, 52.63. HRMS (ESI) calcd. for $C_{30}H_{26}BrCl_2N_2O_2$ $[M+H]^+$: 595.0549, found: 595.0545.

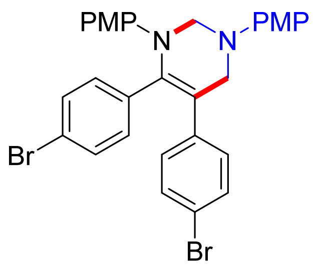

5,6-bis(4-bromophenyl)-1,3-bis(4-methoxyphenyl)-1,2,3,4-tetrahydropyrimidine (3e):

This compound was prepared via general procedure as a white solid (141mg, yield:

78%), mp: 169-171 °C.

^{1}H NMR (400 MHz, $CDCl_3$) δ 7.31 (d, *J* = 8.0 Hz, 2H), 7.14 (d, *J* = 8.0 Hz, 2H), 7.00 (t, *J* = 8.0 Hz, 4H), 6.87 (d, *J* = 8.0 Hz, 2H), 6.80-6.71 (m, 4H), 6.65 (d, *J* = 8.0 Hz, 2H), 4.76 (s, 2H), 4.08 (s, 2H), 3.73 (s, 3H), 3.69 (s, 3H). ^{13}C NMR (100 MHz, $CDCl_3$) δ 155.89, 153.79, 142.22, 140.58, 140.45, 138.70, 135.52, 132.41, 131.31, 131.00, 127.53, 126.11, 121.39, 120.17, 119.19, 117.82, 114.58, 113.98, 72.63, 55.65, 55.30, 52.86. HRMS (ESI) calcd. for $C_{30}H_{27}Br_2N_2O_2$ $[M+H]^+$: 605.0434, found: 605.0435.

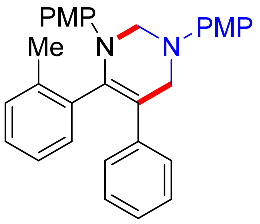

1,3-bis(4-methoxyphenyl)-5-phenyl-6-(o-tolyl)-1,2,3,4-tetrahydropyrimidine (3f):

This compound was prepared via general procedure as yellow oil (85 mg, yield: 61%).

^{1}H NMR (400 MHz, $CDCl_3$) δ 7.14-7.07 (m, 3H), 7.07-7.01 (m, 3H), 6.96-6.91 (m, 1H), 6.88-6.82 (m, 6H), 6.81-6.76 (m, 2H), 6.63-6.57 (m, 2H), 4.89-4.75 (m, 2H), 4.29-4.14 (m, 2H), 3.74 (s, 3H), 3.65 (s, 3H), 2.17 (s, 3H). ^{13}C NMR (100 MHz, $CDCl_3$) δ 155.70, 153.69, 142.75, 140.58, 140.42, 140.00, 137.40, 135.95, 132.45, 130.00, 128.97, 127.72, 127.39, 125.91, 125.74, 124.95, 118.21, 118.09, 114.54, 113.64, 71.57, 55.66, 55.24, 52.68, 20.15. HRMS (ESI) calcd. for $C_{31}H_{30}N_2NaO_2$ $[M+Na]^+$: 485.2199, found: 485.2194.

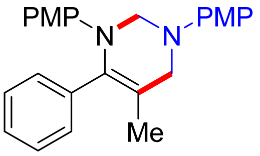

1,3-bis(4-methoxyphenyl)-5-methyl-6-phenyl-1,2,3,4-tetrahydropyrimidine (3g):

This compound was prepared via general procedure as yellow oil (83 mg, yield: 72%).

^{1}H NMR (400 MHz, $CDCl_3$) δ 7.45-7.39 (m, 2H), 7.25 (t, *J* = 8.0 Hz, 2H), 7.17 (t, *J* =

8.0 Hz, 1H), 6.96-6.91 (m, 2H), 6.82-6.77 (m, 2H), 6.76-6.71 (m, 2H), 6.69-6.63 (m, 2H), 4.74 (s, 2H), 3.77 (s, 2H), 3.76 (s, 3H), 3.69 (s, 3H), 1.92 (s, 3H). ^{13}C NMR (100 MHz, $CDCl_3$) δ 155.20, 153.27, 142.70, 141.82, 137.96, 137.42, 130.02, 127.70, 127.15, 125.28, 117.17, 115.32, 114.53, 113.79, 72.41, 55.68, 55.28, 53.66, 17.54. HRMS (ESI) calcd. for $C_{25}H_{27}N_2O_2$ $[M+H]^+$: 387.2067, found: 387.2069.

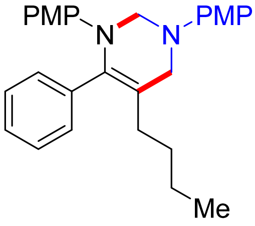

5-butyl-1,3-bis(4-methoxyphenyl)-6-phenyl-1,2,3,4-tetrahydropyrimidine (3h):

This compound was prepared via general procedure as yellow oil (75 mg, yield: 58%).

^{1}H NMR (300 MHz, $CDCl_3$) δ 7.38-7.32 m, 2H), 7.24-7.10 (m, 3H), 6.93-6.86 (m, 2H), 6.79-6.69 (m, 4H), 6.65-6.59 (m, 2H), 4.69 (s, 2H), 3.80 (s, 2H), 3.73 (s, 3H), 3.65 (s, 3H), 2.26-2.14 (m, 2H), 1.61-1.53 (m, 2H), 1.37-1.27 (m, 2H), 0.88 (t, J = 8.0 Hz, 3H). ^{13}C NMR (75 MHz, $CDCl_3$) δ 155.23, 153.27, 142.86, 141.69, 138.44, 137.57, 129.88, 127.66, 127.18, 125.38, 119.92, 117.23, 114.53, 113.75, 72.24, 55.68, 55.26, 51.65, 32.20, 30.96, 22.72, 14.00. HRMS (ESI) calcd. for $C_{28}H_{33}N_2O_2$ $[M+H]^+$: 429.2537, found: 429.2536.

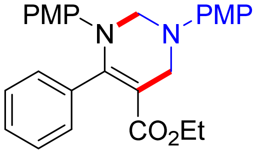

ethyl 1,3-bis(4-methoxyphenyl)-6-phenyl-1,2,3,4-tetrahydropyrimidine-5-carboxylate (3i): [4]

This compound was prepared via general procedure as a white solid (99 mg, yield: 74%).

^{1}H NMR (400 MHz, $CDCl_3$) δ 7.13 (s, 5H), 6.92 (d, J =12.0 Hz, 2H), 6.78 (d, J =12.0 Hz, 2H), 6.62 (d, J = 8.0 Hz, 2H), 6.52 (d, J = 8.0 Hz, 2H), 4.84 (s, 2H), 4.27 (s, 2H),

3.90 (q, J = 8.0 Hz, 2H), 3.75 (s, 3H), 3.64 (s, 3H), 0.87 (t, J = 8.0 Hz, 3H). ^{13}C NMR (100 MHz, $CDCl_3$) δ 167.84, 156.70, 154.86, 154.18, 142.39, 138.90, 136.60, 129.76, 128.13, 128.05, 127.45, 119.30, 114.48, 113.68, 102.29, 71.97, 59.39, 55.62, 55.22, 49.66, 13.81.

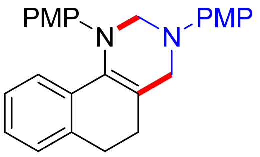

1,3-bis(4-methoxyphenyl)-1,2,3,4,5,6-hexahydrobenzo[h]quinazoline (3j):

This compound was prepared via general procedure as a white solid (106 mg, yield: 89%), mp: 144-146 °C.

^{1}H NMR (400 MHz, $CDCl_3$) δ 7.13 (d, J = 4.0 Hz, 1H), 7.10-7.03 (m, 2H), 7.02-6.93 (m, 3H), 6.82-6.74 (m, 4H), 6.71 (d, J = 8.0 Hz, 2H), 4.74 (s, 2H), 3.85 (s, 2H), 3.72 (s, 3H), 3.73 (s, 3H), 2.93 (t, J = 8.0 Hz, 2H), 2.40 (t, J = 8.0 Hz, 2H). ^{13}C NMR (100 MHz, $CDCl_3$) δ 155.15, 153.29, 148.26, 142.61, 142.02, 136.02, 134.85, 131.78, 127.32, 126.57, 126.27, 123.59, 121.74, 116.96, 114.58, 114.28, 72.24, 55.69, 55.43, 52.12, 28.22, 26.20. HRMS (ESI) calcd. for $C_{26}H_{27}N_2O_2$ $[M+H]^+$: 399.2067, found: 399.2065.

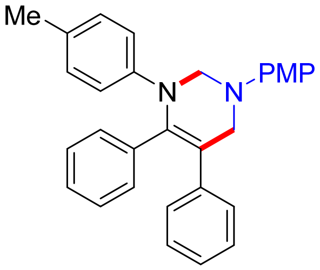

3-(4-methoxyphenyl)-5,6-diphenyl-1-(p-tolyl)-1,2,3,4-tetrahydropyrimidine (3k):

This compound was prepared via general procedure as yellow oil (97 mg, yield: 75%).

^{1}H NMR (400 MHz, $CDCl_3$) δ 7.23-7.11 (m, 7H), 7.04-6.96 (m, 3H), 6.93-6.83 (m, 4H), 6.78 (s, 4H), 4.87 (s, 2H), 4.14 (s, 2H), 3.74 (s, 3H), 2.17 (s, 3H). ^{13}C NMR (125 MHz, $CDCl_3$) δ 153.55, 145.17, 142.48, 140.43, 140.15, 136.82, 132.13, 130.83,

129.81, 129.17, 127.97, 127.48, 127.04, 126.09, 124.24, 120.57, 117.64, 114.53, 72.15, 55.65, 53.21, 20.73. HRMS (ESI) calcd. for $C_{30}H_{29}N_2O$ $[M+H]^+$: 433.2274, found: 433.2278.

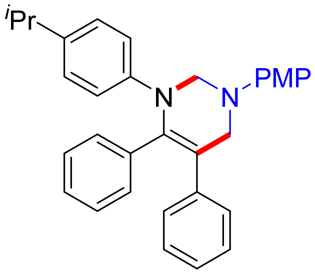

1-(4-isopropylphenyl)-3-(4-methoxyphenyl)-5,6-diphenyl-1,2,3,4-tetrahydropyrimidine (3l):

This compound was prepared via general procedure as yellow oil (94 mg, yield: 68%).

^{1}H NMR (400 MHz, $CDCl_3$) δ 7.22-7.11 (m, 7H), 7.03-6.98 (m, 3H), 6.94 (d, *J* = 8.0 Hz, 2H), 6.88 (d, *J* = 8.0 Hz, 2H), 6.77 (s, 4H), 4.88 (s, 2H), 4.14 (s, 2H), 3.73 (s, 3H), 2.78-2.68 (m, 1H), 1.14 (s, 3H), 1.12 (s, 3H). ^{13}C NMR (101 MHz, $CDCl_3$) δ 153.51, 145.33, 142.93, 142.50, 140.40, 140.19, 136.86, 130.81, 129.81, 128.00, 127.50, 127.03, 126.50, 126.13, 123.92, 120.85, 117.57, 114.51, 71.98, 55.65, 53.30, 33.28, 23.95. HRMS (ESI) calcd. for $C_{32}H_{33}N_2O$ $[M+H]^+$: 461.2587, found: 461.2588.

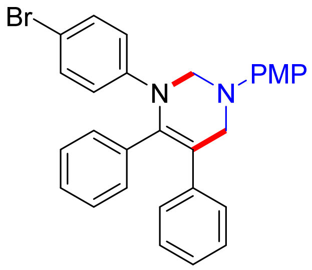

1-(4-bromophenyl)-3-(4-methoxyphenyl)-5,6-diphenyl-1,2,3,4-tetrahydropyrimidine (3m):

This compound was prepared via general procedure as yellow oil (77 mg, yield: 52%).

^{1}H NMR (400 MHz, $CDCl_3$) δ 7.21-7.11 (m, 9H), 7.04-7.97 (m, 3H), 6.80 (d, *J* = 8.0

Hz, 2H), 6.78 (s, 4H), 4.88 (s, 2H), 4.14 (s, 2H), 3.73 (s, 3H). ^{13}C NMR (100 MHz, $CDCl_3$) δ 153.85, 146.82, 142.17, 139.71, 139.68, 136.29, 131.52, 130.71, 129.78, 128.08, 127.69, 127.33, 126.39, 125.73, 121.68, 117.97, 115.55, 114.59, 72.03, 55.67, 53.30. HRMS (ESI) calcd. for $C_{29}H_{26}BrN_2O$ $[M+H]^+$: 497.1223, found: 497.1221.

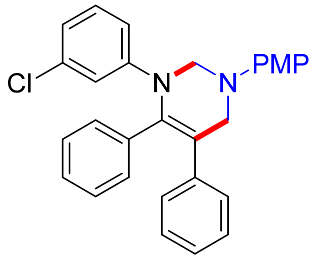

1-(3-chlorophenyl)-3-(4-methoxyphenyl)-5,6-diphenyl-1,2,3,4-tetrahydropyrimidine (3n):

This compound was prepared via general procedure as yellow oil (64 mg, yield: 47%).

^{1}H NMR (400 MHz, $CDCl_3$) δ 7.21-7.12 (m, 7H), 7.03-6.98 (m, 3H), 6.96 (d, J = 8.0 Hz, 1H), 6.92 (t, J = 4.0 Hz, 1H), 6.83-6.74 (m, 6H), 4.90 (s, 2H), 4.15 (s, 2H), 3.73 (s, 3H). ^{13}C NMR (100 MHz, $CDCl_3$) δ 153.89, 148.96, 142.18, 139.67, 139.54, 136.23, 133.98, 130.66, 129.78, 129.37, 128.09, 127.70, 127.35, 126.43, 124.14, 122.69, 122.26, 121.90, 118.08, 114.57, 71.90, 55.65, 53.36. HRMS (ESI) calcd. for $C_{29}H_{26}ClN_2O$ $[M+H]^+$: 453.1728, found: 453.1725.

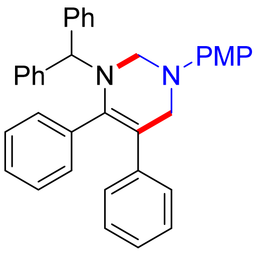

1-benzhydryl-3-(4-methoxyphenyl)-5,6-diphenyl-1,2,3,4-tetrahydropyrimidine (3o):

This compound was prepared via general procedure as a white solid (110 mg, yield: 72%), mp: 186-188 °C.

^{1}H NMR (300 MHz, $CDCl_3$) δ 7.25-7.16 (m, 12H), 7.12-6.99 (m, 8H), 6.76-6.69 (m,

2H), 6.56-6.49 (m, 2H), 5.56 (s, 1H), 4.47 (s, 2H), 3.83 (s, 2H), 3.73 (s, 3H). ^{13}C NMR (100 MHz, $CDCl_3$) δ 153.13, 142.76, 142.63, 140.88, 140.67, 137.07, 130.80, 129.65, 129.02, 128.00, 127.91, 127.72, 127.48, 126.92, 125.49, 116.94, 116.64, 114.32, 66.23, 63.93, 61.29, 55.63, 52.48. HRMS (ESI) calcd. for $C_{36}H_{33}N_2O$ $[M+H]^+$: 509.2587, found: 509.2589.

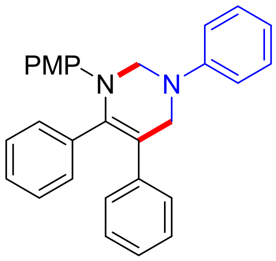

1-(4-methoxyphenyl)-3,5,6-triphenyl-1,2,3,4-tetrahydropyrimidine (3p):

This compound was prepared via general procedure as yellow oil (109 mg, yield: 87%).

^1H NMR (400 MHz, $CDCl_3$) δ 7.24-7.11 (m, 9H), 7.04-6.92 (m, 5H), 6.83-6.75 (m, 3H), 6.71-6.62 (m, 2H), 4.93 (s, 2H), 4.22 (s, 2H), 3.67 (s, 3H). ^{13}C NMR (100 MHz, $CDCl_3$) δ 155.65, 148.18, 141.02, 140.92, 140.07, 136.78, 130.93, 129.76, 129.21, 128.04, 127.54, 127.18, 126.18, 125.88, 120.26, 119.19, 115.26, 113.93, 70.82, 55.28, 52.00. HRMS (ESI) calcd. for $C_{29}H_{27}N_2O$ $[M+H]^+$: 419.2118, found: 419.2115.

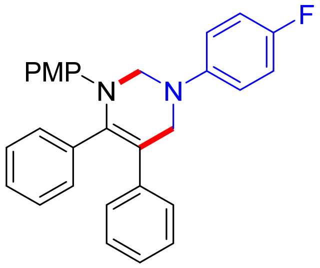

3-(4-fluorophenyl)-1-(4-methoxyphenyl)-5,6-diphenyl-1,2,3,4-tetrahydropyrimidine (3q):

This compound was prepared via general procedure as yellow oil (92 mg, yield: 70%).

^1H NMR (400 MHz, $CDCl_3$) δ 7.22-7.12 (m, 7H), 7.03-6.96 (m, 3H), 6.94-6.85 (m,

4H), 6.77-6.69 (m, 2H), 6.67-6.61 (m, 2H), 4.85 (s, 2H), 4.16 (s, 2H), 3.67 (s, 3H). ^{13}C NMR (100 MHz, $CDCl_3$) δ 155.63, 144.80, 144.78, 140.93, 139.98, 136.68, 130.90, 129.76, 128.04, 127.54, 127.18, 126.19, 125.85, 119.95, 117.16, 117.09, 115.70, 115.48, 113.91, 72.00, 55.27, 52.74. HRMS (ESI) calcd. for $C_{29}H_{26}FN_2O$ $[M+H]^+$: 437.2024, found: 437.2025.

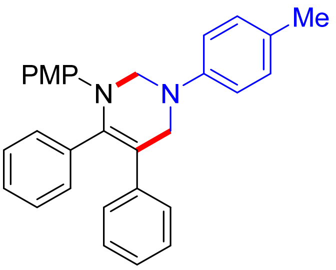

1-(4-methoxyphenyl)-5,6-diphenyl-3-(p-tolyl)-1,2,3,4-tetrahydropyrimidine (3r):

This compound was prepared via general procedure as yellow oil (114 mg, yield: 88%).

^{1}H NMR (400 MHz, $CDCl_3$) δ 7.20-7.09 (m, 7H), 7.01-6.96 (m, 5H), 6.93 (d, J = 8.0 Hz, 2H), 6.69 (d, J = 8.0 Hz, 2H), 6.63 (d, J = 8.0 Hz, 2H), 4.85 (s, 2H), 4.16 (s, 2H), 3.64 (s, 3H), 2.22 (s, 3H). ^{13}C NMR (100 MHz, $CDCl_3$) δ 155.63, 146.06, 141.08, 140.93, 140.18, 136.87, 130.96, 129.81, 129.72, 128.72, 128.04, 127.54, 127.15, 126.15, 125.95, 120.28, 115.68, 113.91, 71.45, 55.29, 52.43, 20.48. HRMS (ESI) calcd. for $C_{30}H_{29}N_2O$ $[M+H]^+$: 433.2274, found: 433.2275.

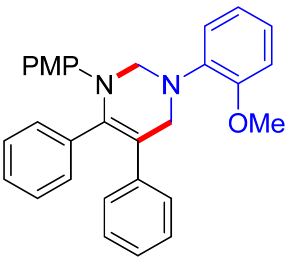

3-(2-methoxyphenyl)-1-(4-methoxyphenyl)-5,6-diphenyl-1,2,3,4-tetrahydropyrimidine (3s):

This compound was prepared via general procedure as colorless oil (104 mg, yield: 77%).

^{1}H NMR (400 MHz, $CDCl_3$) δ 7.20-7.10 (m, 7H), 7.06 (dd, J = 8.0, 4.0 Hz, 1H), 7.01 -6.95 (m, 3H), 6.93-6.85 (m, 2H), 6.84-6.79 (m, 2H), 6.70 (dd, J = 8.0, 4.0 Hz, 1H), 6.62-6.55 (m, 2H), 4.87 (s, 2H), 4.20 (s, 2H), 3.64 (s, 3H), 3.44 (s, 3H). ^{13}C NMR (100 MHz, $CDCl_3$) δ 154.86, 152.20, 141.32, 140.40, 138.56, 137.71, 136.91, 130.97, 129.99, 127.94, 127.45, 126.92, 125.92, 125.74, 122.77, 120.74, 119.41, 118.34, 113.37, 111.38, 71.09, 55.32, 55.17, 53.49. HRMS (ESI) calcd. for $C_{30}H_{29}N_2O_2$ $[M+H]^+$: 449.2224, found: 449.2221.

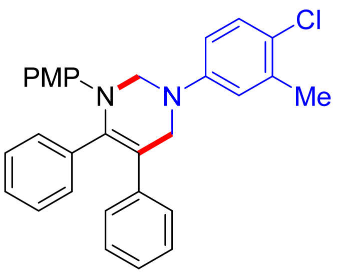

3-(4-chloro-3-methylphenyl)-1-(4-methoxyphenyl)-5,6-diphenyl-1,2,3,4-tetrahydropyrimidine (3t):

This compound was prepared via general procedure as yellow oil (109 mg, yield: 78%).

^{1}H NMR (400 MHz, $CDCl_3$) δ 7.22-7.11 (m, 7H), 7.03-6.96 (m, 4H), 6.93 (d, J = 8.0 Hz, 2H), 6.75 (d, J = 4.0 Hz, 1H), 6.65 (d, J = 8.0 Hz, 2H), 6.54 (dd, J = 8.0, 4.0 Hz, 1H), 4.86 (s, 2H), 4.16 (s, 2H), 3.67 (s, 3H), 2.23 (s, 3H). ^{13}C NMR (75 MHz, $CDCl_3$) δ 155.73, 147.38, 140.95, 140.76, 139.85, 136.61, 134.80, 131.15, 130.88, 129.69, 128.03, 127.52, 127.20, 126.22, 126.12, 125.81, 120.01, 115.61, 113.97, 113.87, 70.46, 55.28, 52.14, 18.96. HRMS (ESI) calcd. for $C_{30}H_{28}ClN_2O$ $[M+H]^+$: 467.1885, found: 467.1884,.

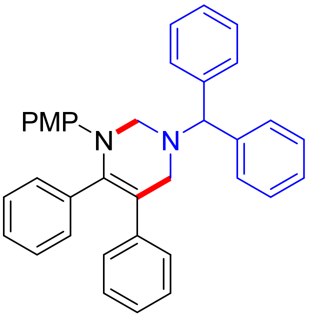

3-benzhydryl-1-(4-methoxyphenyl)-5,6-diphenyl-1,2,3,4-tetrahydropyrimidine (3u):

This compound was prepared via general procedure as a white solid (120 mg, yield: 79%), mp: 200-202 °C.

^{1}H NMR (400 MHz, $CDCl_3$) δ 7.24-7.19 (m, 4H), 7.18-7.10 (m, 8H), 7.09-7.02 (m, 3H), 7.00-6.91 (m, 5H), 6.83 (d, J = 8.0 Hz, 2H), 6.64 (d, J = 8.0 Hz, 2H), 4.56 (s, 1H), 4.21 (s, 2H), 3.72 (s, 3H), 3.50 (s, 2H). ^{13}C NMR (100 MHz, $CDCl_3$) δ 155.23, 142.46, 141.63, 140.44, 139.88, 137.04, 130.82, 129.80, 128.43, 127.77, 127.64, 127.44, 126.95, 126.85, 125.76, 125.65, 120.73, 113.61, 71.43, 70.98, 55.95, 55.44. HRMS (ESI) calcd. for $C_{36}H_{33}N_2O$ $[M+H]^+$: 509.2587, found: 509.2589.

Large scale reaction for Scheme 2

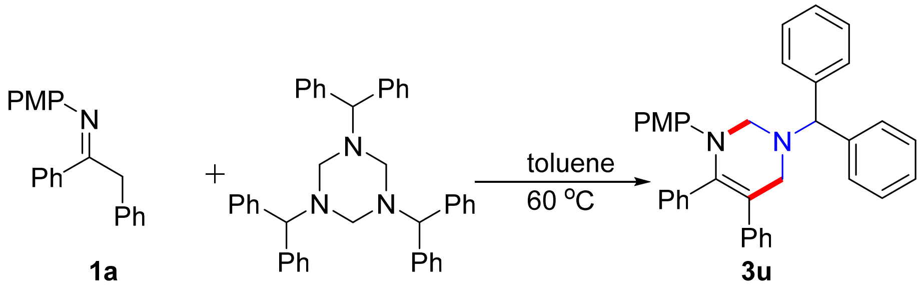

To a flask was added **1a** (5 g, 16.6 mmol), 1,3,5-tribenzhydryl-1,3,5-triazinane (10.7 g, 18.2 mmol) and toluene (300 mL), the resulting solution was stirred at 60 °C for 24 h. The reaction mixture was concentrated, the residue was purified by recrystallization from EtOAc/Hexane to give **3u** as a white solid (6.1 g, yield: 72%).

Mechanistic studies for Scheme 3

1. Scheme 3a

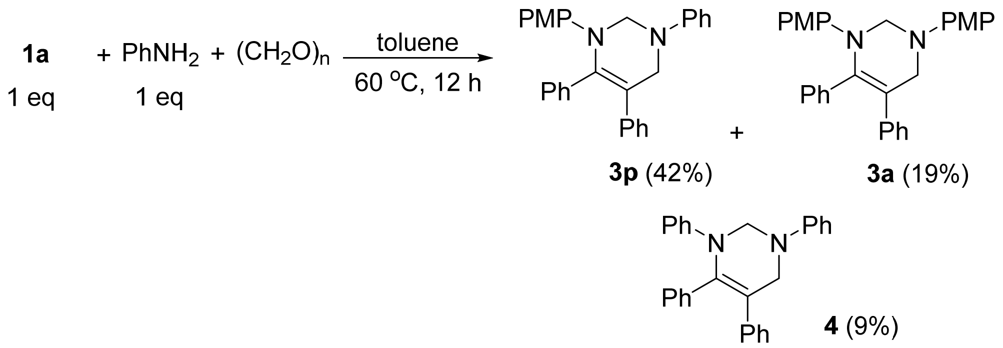

To a tube was added **1a** (0.30 mmol, 1 eq), $PhNH_2$ (0.30 mmol, 1 eq), $(CH_2O)_n$ (0.63 mmol, 2.1 eq) and toluene (6 mL), the resulting mixture was stirred at 60 °C for 12 h. The reaction mixture was concentrated, the residue was purified by flash chromatography on silica gel (petroleum ether/ethyl acetate = 80:1-20:1) to give **3p** (53 mg, yield: 42%), **3a** (26 mg, yield: 19%) as yellow oil and **4** (10 mg, yield: 9%) as yellow oil. Data of **4**: ^{1}H NMR (400 MHz, $CDCl_3$) δ 7.21-7.11 (m, 9H), 7.10-7.04 (m, 2H), 7.02-6.92 (m, 5H), 6.87-6.81(m, 1H), 6.80-6.71(m, 3H), 5.01 (s, 2H), 4.21 (s, 2H). ^{13}C NMR (75 MHz, $CDCl_3$) δ 148.05, 147.42, 140.39, 139.94, 136.59, 130.80, 129.73, 129.17, 128.60, 128.05, 127.56, 127.19, 126.28, 124.17, 122.81, 121.10, 119.21, 115.25, 70.10, 52.18. HRMS (ESI) calcd. for $C_{28}H_{25}N_2$ $[M+H]^+$: 389.2012, found: 389.2016.

2. Scheme 3b

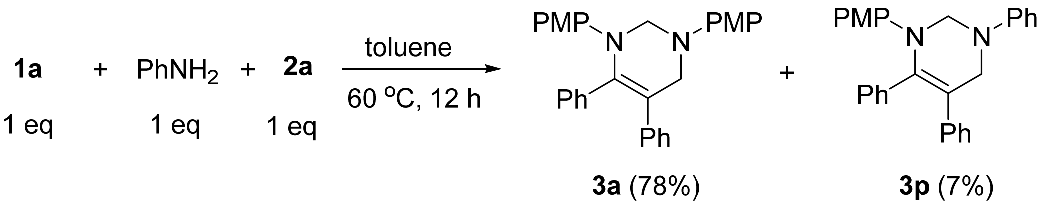

To a tube was added **1a** (0.30 mmol, 1 eq), $PhNH_2$ (0.30 mmol, 1 eq), **2a** (0.30 mmol, 1 eq) and toluene (6 mL), the resulting mixture was stirred at 60 °C for 12 h. The reaction mixture was concentrated, the residue was purified by flash chromatography on silica gel (petroleum ether/ethyl acetate = 80:1-20:1) to give **3a** (105 mg, yield: 78%) as yellow oil and **3p** (9 mg, yield: 7%) as yellow oil.

2. Scheme 3c

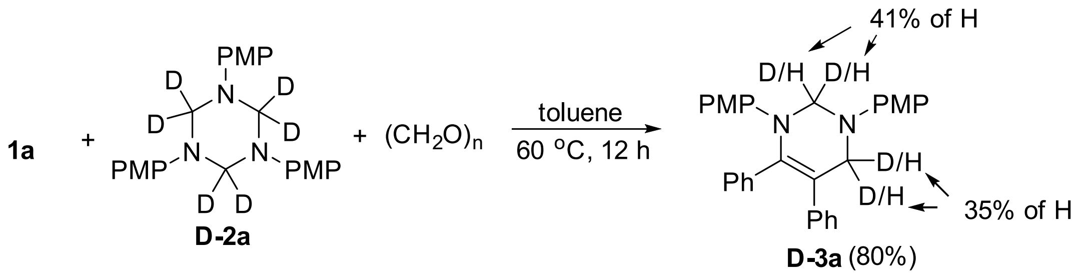

To a tube was added **1a** (0.30 mmol, 1 eq), **D-2a** (0.33 mmol, 1.1 eq), $(CH_2O)_n$ (0.63 mmol, 2.1 eq) and toluene (6 mL), the resulting mixture was stirred at 60 °C for 12 h. The reaction mixture was concentrated, the residue was purified by flash chromatography on silica gel (petroleum ether/ethyl acetate = 80:1-20:1) to give **D-3a** (109 mg, yield: 80%) as yellow oil.

^{1}H NMR (300 MHz, $CDCl_3$) δ 7.20-7.10 (m, 7H), 7.01-6.95 (m, 3H), 6.94-6.87 (m, 2H), 6.76 (s, 4H), 6.63 (d, J = 8.7 Hz, 2H), 4.81 (s, 0.82H), 4.13 (s, 0.7H), 3.72 (s, 3H), 3.65 (s, 3H). ^{13}C NMR (75 MHz, $CDCl_3$) δ 155.52, 153.55, 142.54, 142.51, 141.14, 141.10, 140.87, 140.83, 140.79, 140.76, 140.18, 136.84, 130.92, 129.80, 127.99, 127.49, 127.08, 126.07, 126.06, 125.92, 125.88, 120.10, 119.90, 117.69, 117.64, 114.57, 113.83, 72.64, 72.58, 55.66, 55.26, 53.08, 53.01. HRMS (ESI) calcd. for $C_{30}H_{25}D_4N_2O_2$ $[M+H]^+$: 453.2475, found: 453.2478.

X-ray structure of 3j:

The crystal structures have been deposited at the Cambridge Crystallographic Data Centre (CCDC 1577296, **3j**). The data can be obtained free of charge via the internet at www.ccdc.cam.ac.uk/data_request/cif.

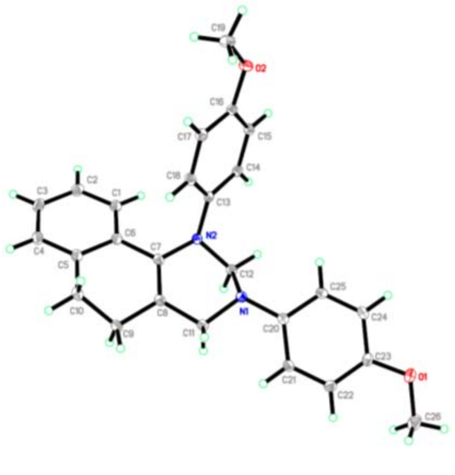

References:

[1] Zhu, C.; Xu, G.; Sun, J. *Angew. Chem. Int. Ed.* **2016**, *55*, 11867.

[2] (a) Gautier, F-M.; Jones, S.; Martin, S. J. *Org. Biomol. Chem.* **2009**, *7*, 229. (b) Saito, K.; Horiguchi, K.; Shibata, Y.; Yamanaka, M.; Akiyama, T. *Chem. Eur. J.* **2014**, *20*, 7616. (c) Lu, B.; Wu, J.; Yoshikai, N. *J. Am. Chem. Soc.* **2014**, *136*, 11598.

[3] Zheng, Y.; Xue, Z.; Liu, L.; Shu, C.; Yuan, W.; Zhang, X. *Org. Biomol. Chem.* **2013**, *11*, 412.

[4] Cao, H.; Wang, X.; Jiang, H.; Zhu, Q.; Zhang, M.; Liu, H. *Chem. Eur. J.* **2008**, *14*, 11623.

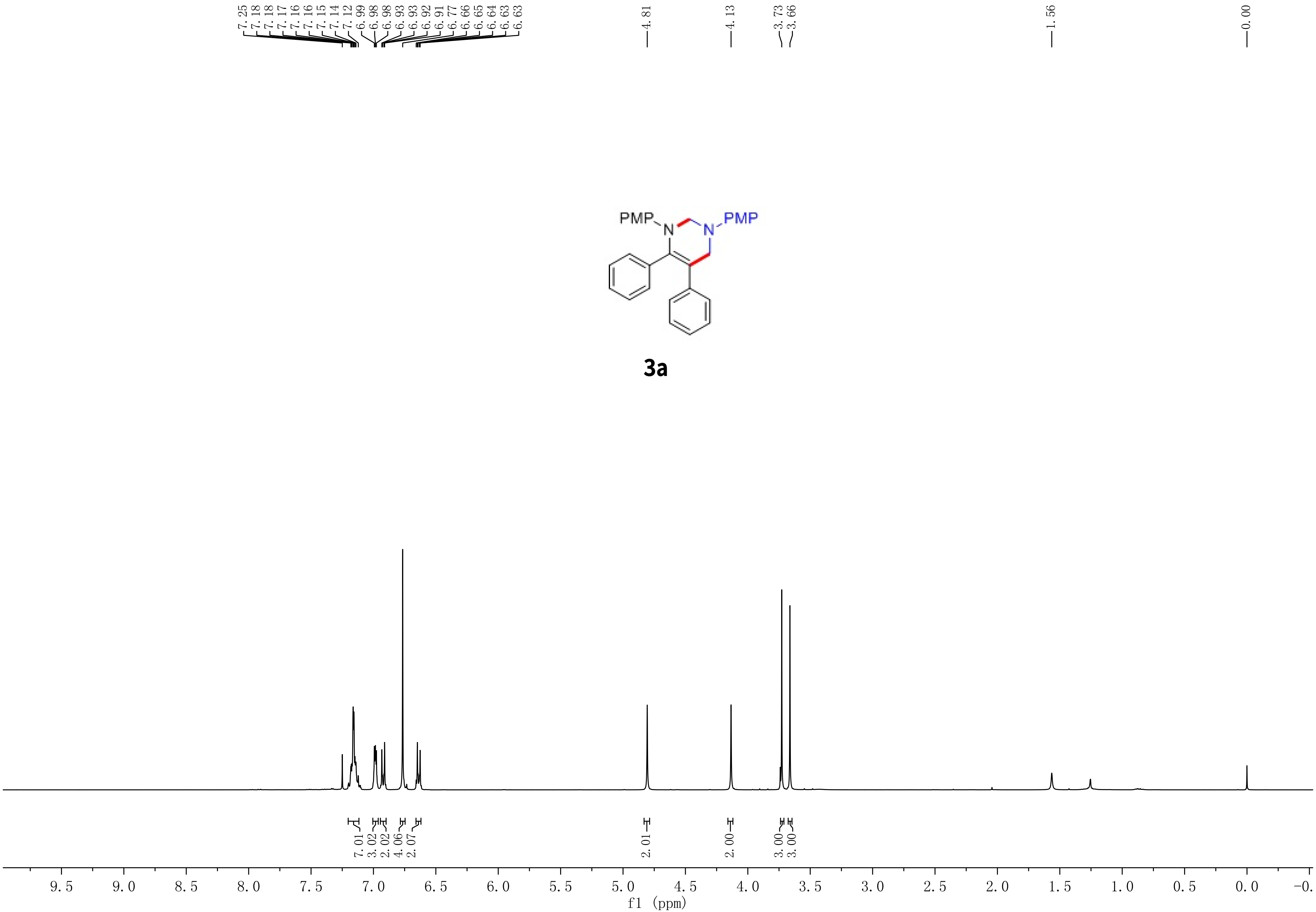
7.25
7.18
7.18
7.17
7.16
7.16
7.15
7.14
7.12
6.99
6.98
6.98
6.93
6.93
6.92
6.91
6.77
6.66
6.65
6.64
6.63
6.63
4.81
4.13
3.73
3.66
1.56
0.00
7.01
3.02
2.02
4.06
2.07
2.01
2.00
3.00
3.00
9.5 9.0 8.5 8.0 7.5 7.0 6.5 6.0 5.5 5.0 4.5 4.0 3.5 3.0 2.5 2.0 1.5 1.0 0.5 0.0 -0.
f1 (ppm)
PMP
N
N
PMP

3a

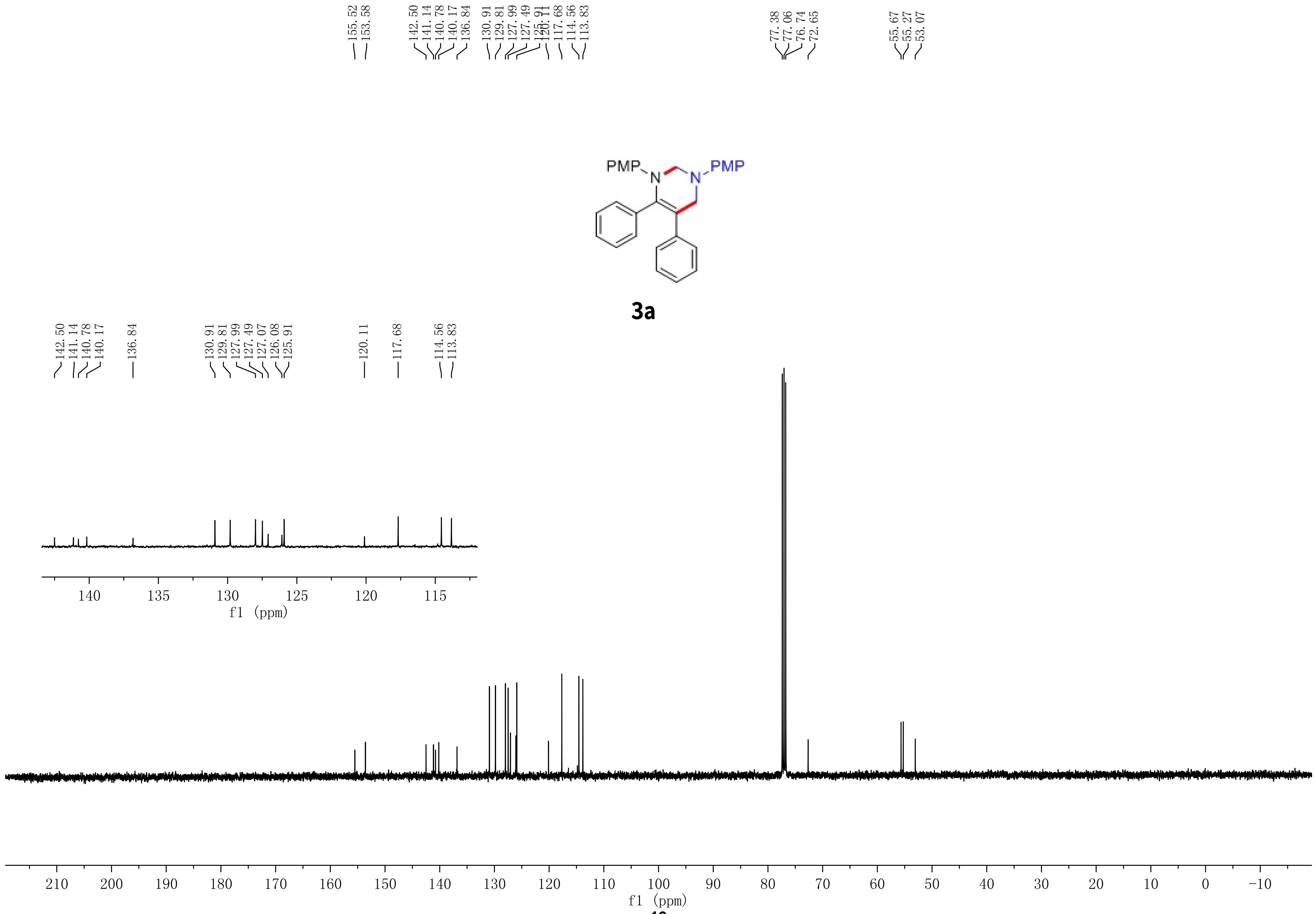
155.52
153.58
142.50
141.14
140.78
140.17
136.84
130.91
129.81
127.99
127.49
125.91
120.11
117.68
114.56
113.83
77.38
77.06
76.74
72.65
55.67
55.27
53.07
PMP
N
N
PMP
3a
142.50
141.14
140.78
140.17
136.84
130.91
129.81
127.99
127.49
127.07
126.08
125.91
120.11
117.68
114.56
113.83
140
135
130
125
120
115
f1 (ppm)
210
200
190
180
170
160
150
140
130
120
110
100
90
80
70
60
50
40
30
20
10
0
-10
f1 (ppm)

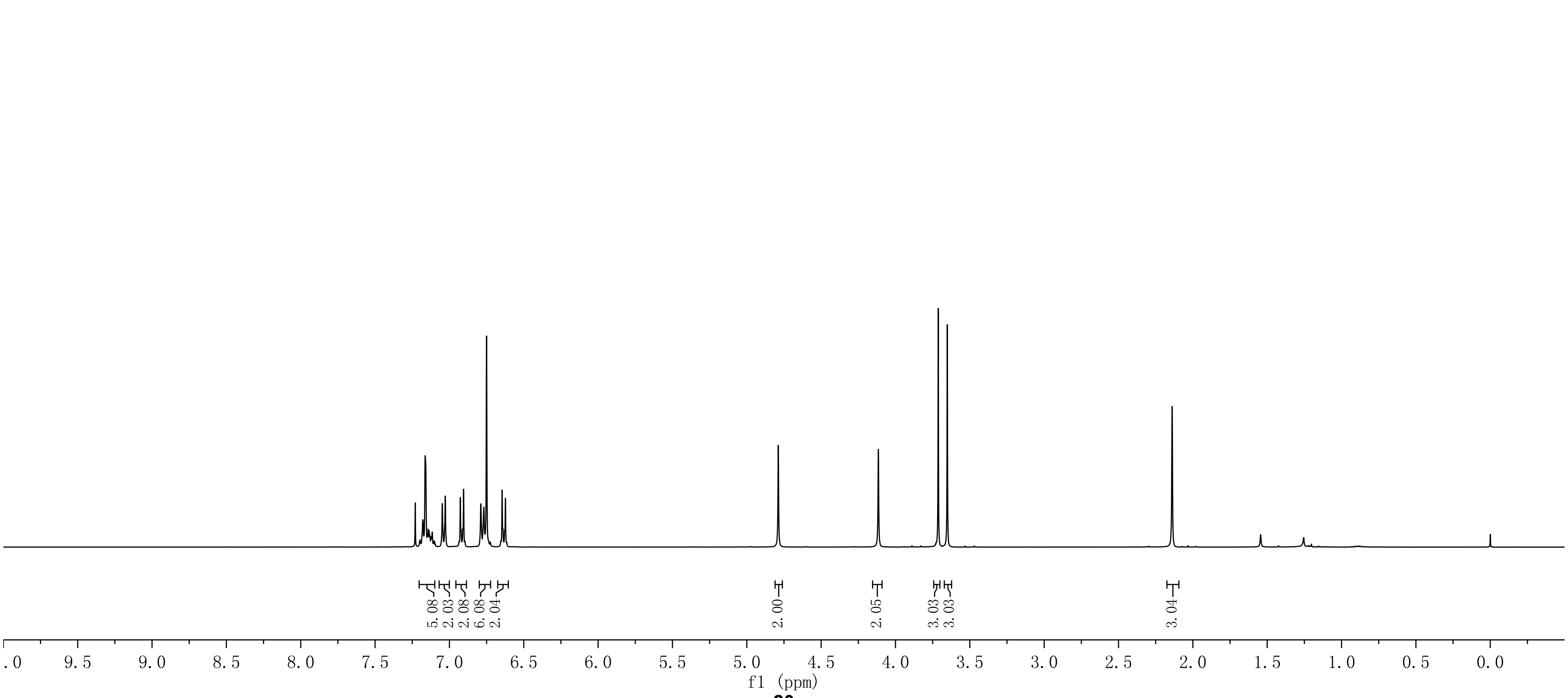
7.23 7.20 7.18 7.18 7.16 7.15 7.14 7.14 7.13 7.12 7.12 7.11 7.10 7.05 7.03 6.93 6.92 6.91 6.90 6.90 6.79 6.77 6.75 6.65 6.65 6.64 6.63 6.62
4.79
4.12
3.71
3.65
2.14
1.54
-0.00
5.08 2.03 2.08 6.08 2.04
2.00
2.05
3.03 3.03
3.04
.0 9.5 9.0 8.5 8.0 7.5 7.0 6.5 6.0 5.5 5.0 4.5 4.0 3.5 3.0 2.5 2.0 1.5 1.0 0.5 0.0
f1 (ppm)

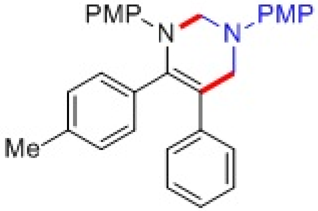
PMP
N
N
PMP
Me

3b

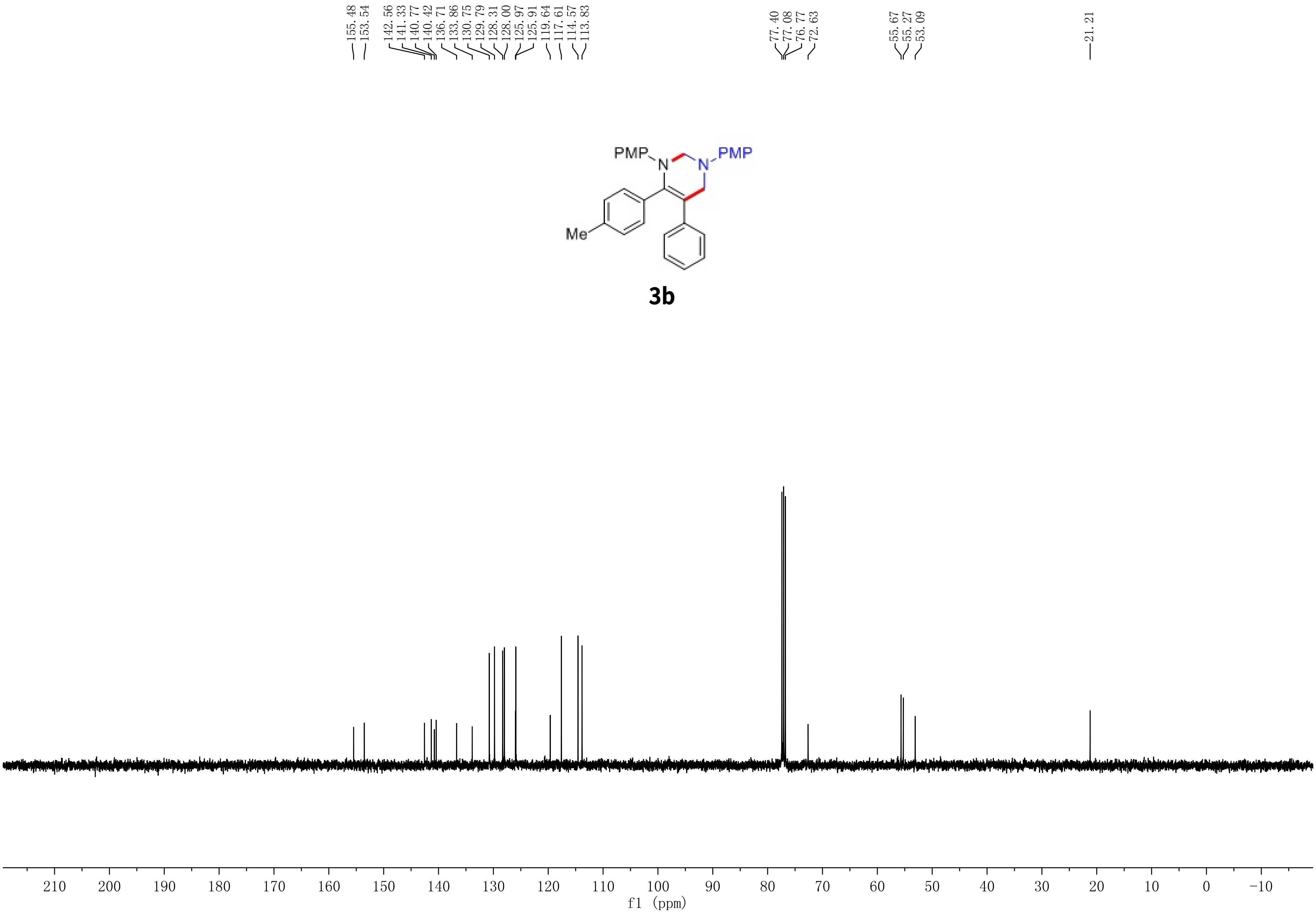
155.48
153.54
142.56
141.33
140.77
140.42
136.71
133.86
130.75
129.79
128.31
128.00
125.97
125.91
119.64
117.61
114.57
113.83
77.40
77.08
76.77
72.63
55.67
55.27
53.09
21.21
PMP
N
N
PMP
Me
3b
210 200 190 180 170 160 150 140 130 120 110 100 90 80 70 60 50 40 30 20 10 0 -10
f1 (ppm)

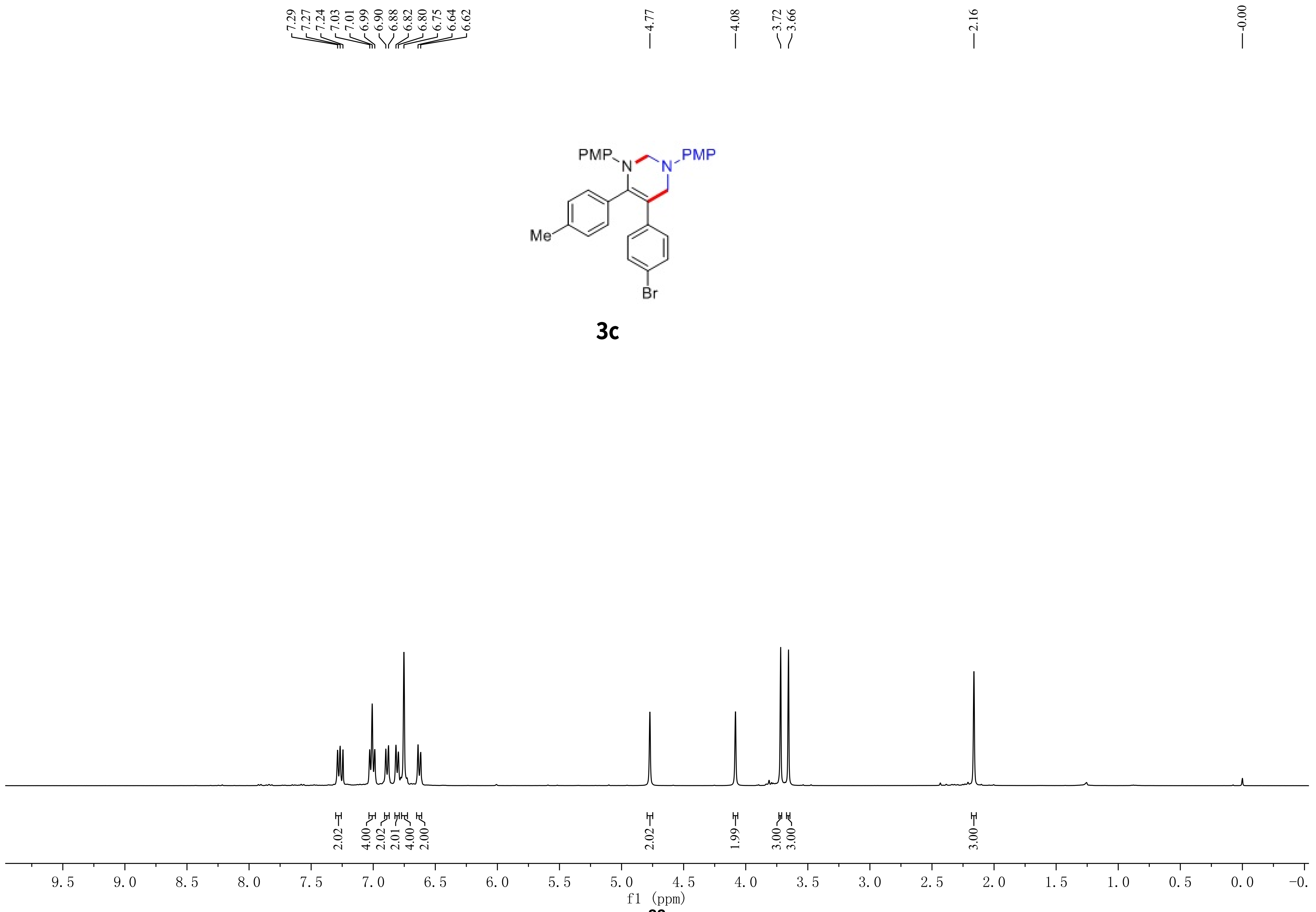
7.29
7.27
7.24
7.03
7.01
6.99
6.90
6.88
6.82
6.80
6.75
6.64
6.62
4.77
4.08
3.72
3.66
2.16
-0.00
PMP
N
N
PMP
Me
Br
2.02
4.00
2.02
2.01
4.00
2.00
2.02
1.99
3.00
3.00
3.00
9.5
9.0
8.5
8.0
7.5
7.0
6.5
6.0
5.5
5.0
4.5
4.0
3.5
3.0
2.5
2.0
1.5
1.0
0.5
0.0
f1 (ppm)

3c

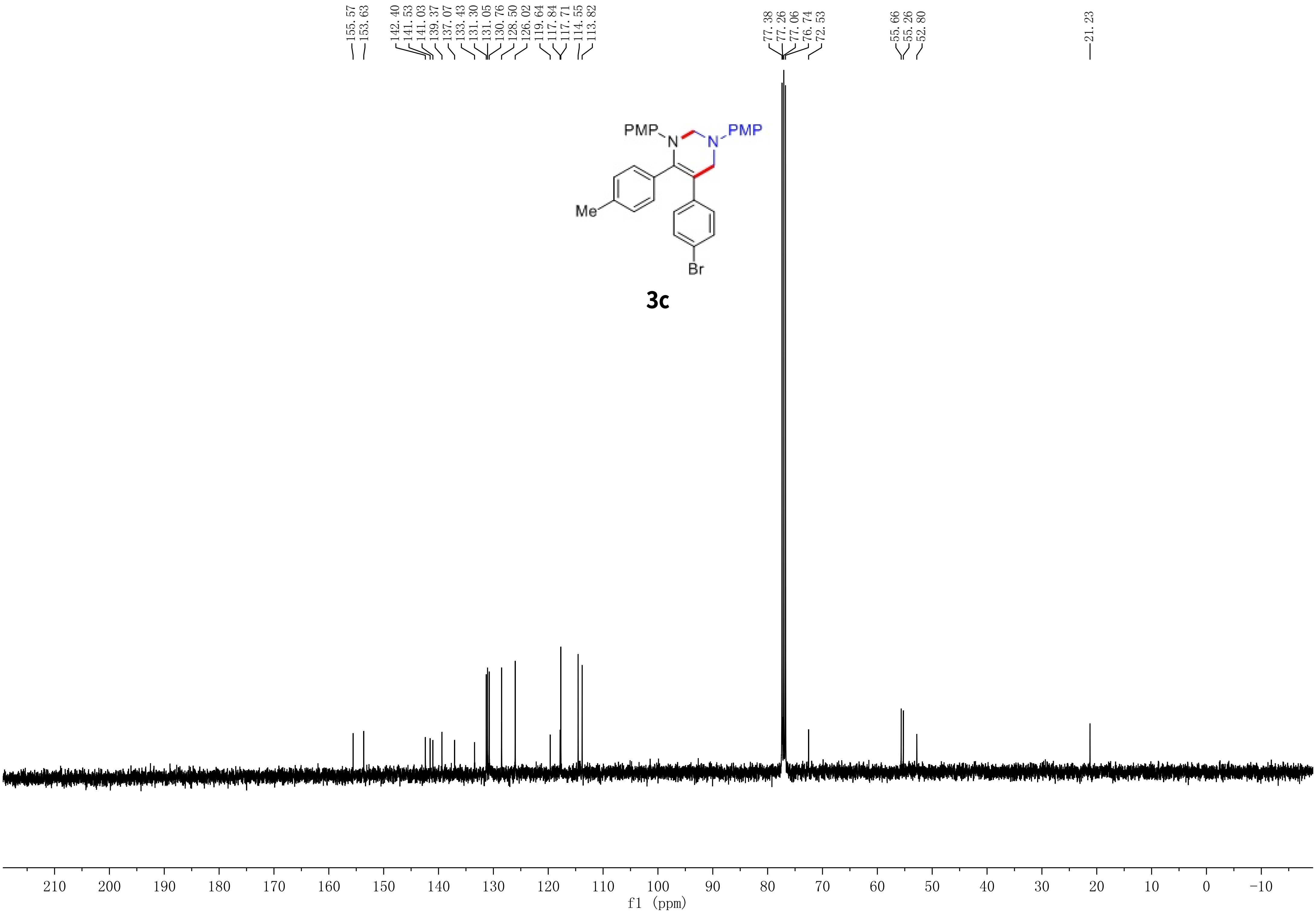
155.57
153.63
142.40
141.53
141.03
139.37
137.07
133.43
131.30
131.05
130.76
128.50
126.02
119.64
117.84
117.71
114.55
113.82
77.38
77.26
77.06
76.74
72.53
55.66
55.26
52.80
21.23
PMP
N
N
PMP
Me
Br
3c
210 200 190 180 170 160 150 140 130 120 110 100 90 80 70 60 50 40 30 20 10 0 -10
f1 (ppm)

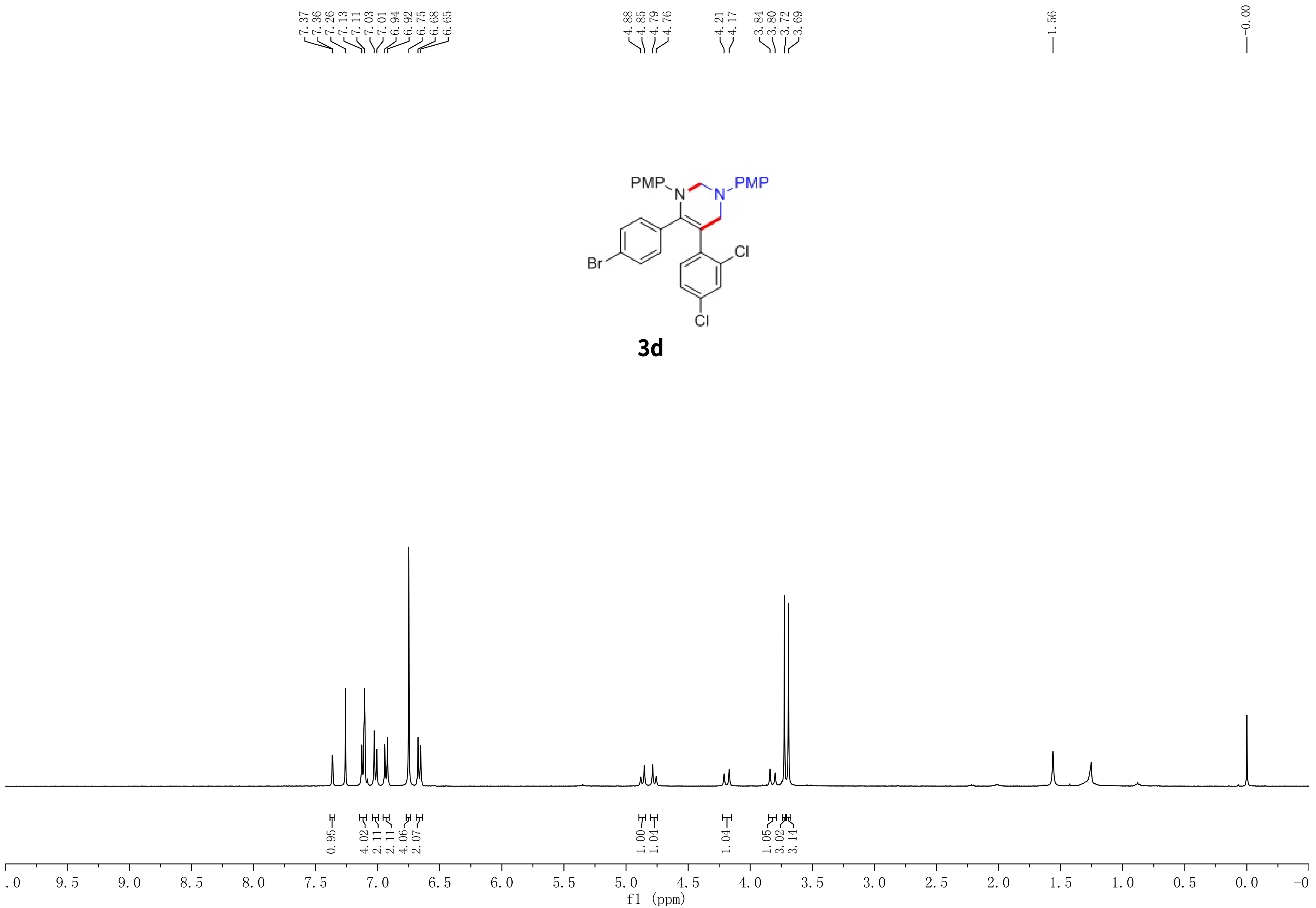
7.37
7.36
7.26
7.13
7.11
7.03
7.01
6.94
6.92
6.75
6.68
6.65
4.88
4.85
4.79
4.76
4.21
4.17
3.84
3.80
3.72
3.69
1.56
-0.00
PMP
N
PMP
N
Br
Cl
Cl
3d
0.95
4.02
2.11
2.11
4.06
2.07
1.00
1.04
1.04
1.05
3.02
3.14
9.5
9.0
8.5
8.0
7.5
7.0
6.5
6.0
5.5
5.0
4.5
4.0
3.5
3.0
2.5
2.0
1.5
1.0
0.5
0.0
f1 (ppm)

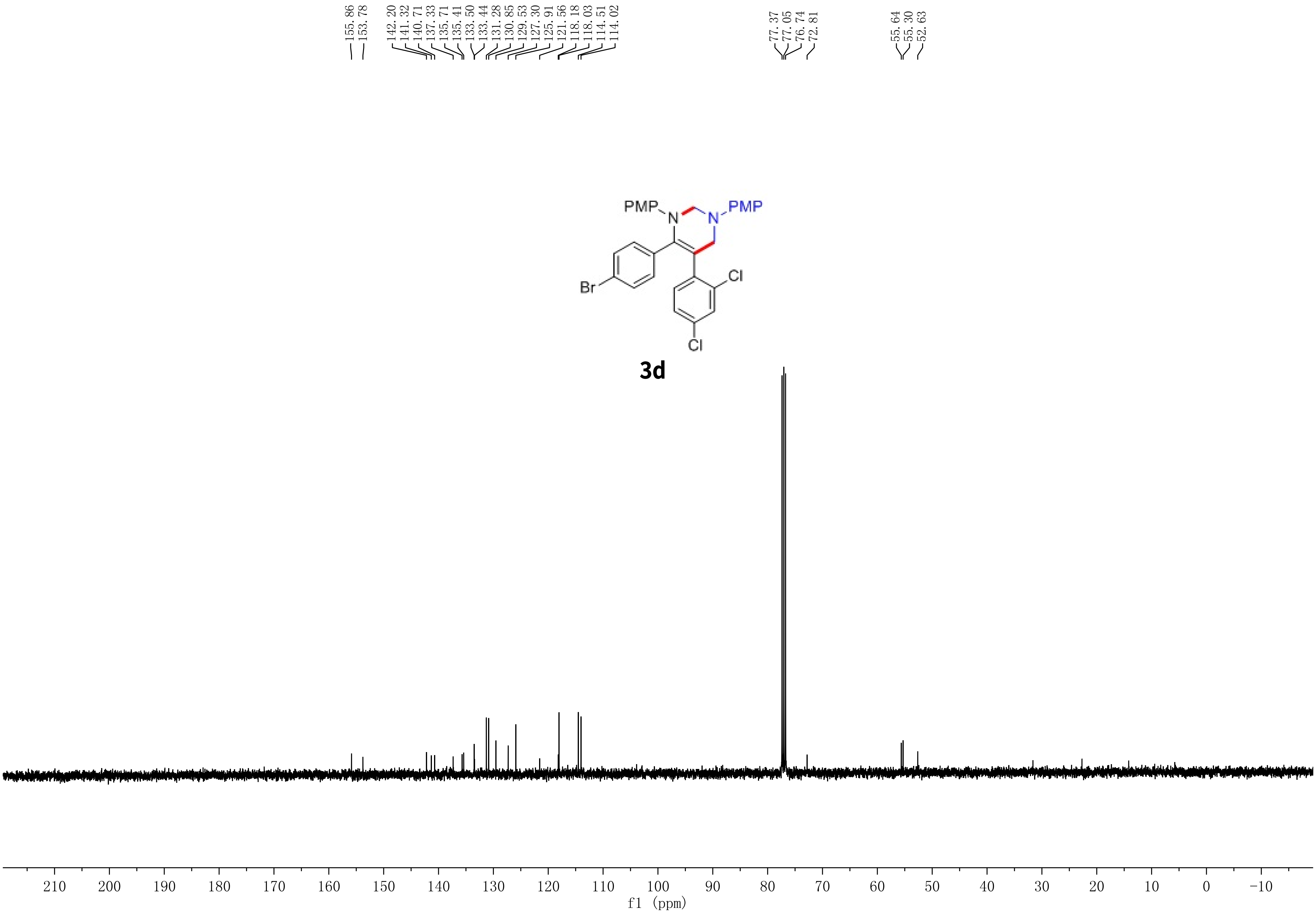
155.86
153.78
142.20
141.32
140.71
137.33
135.71
135.41
133.50
133.44
131.28
130.85
129.53
127.30
125.91
121.56
118.18
118.03
114.51
114.02
77.37
77.05
76.74
72.81
55.64
55.30
52.63
PMP
N
N
PMP
Br
Cl
Cl
3d
210
200
190
180
170
160
150
140
130
120
110
100
90
80
70
60
50
40
30
20
10
0
−10
f1 (ppm)

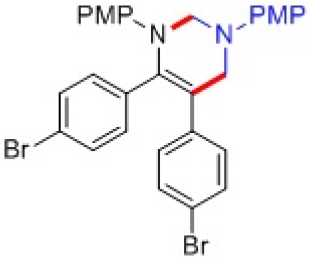
PMP
N
N
PMP
Br
Br

3e

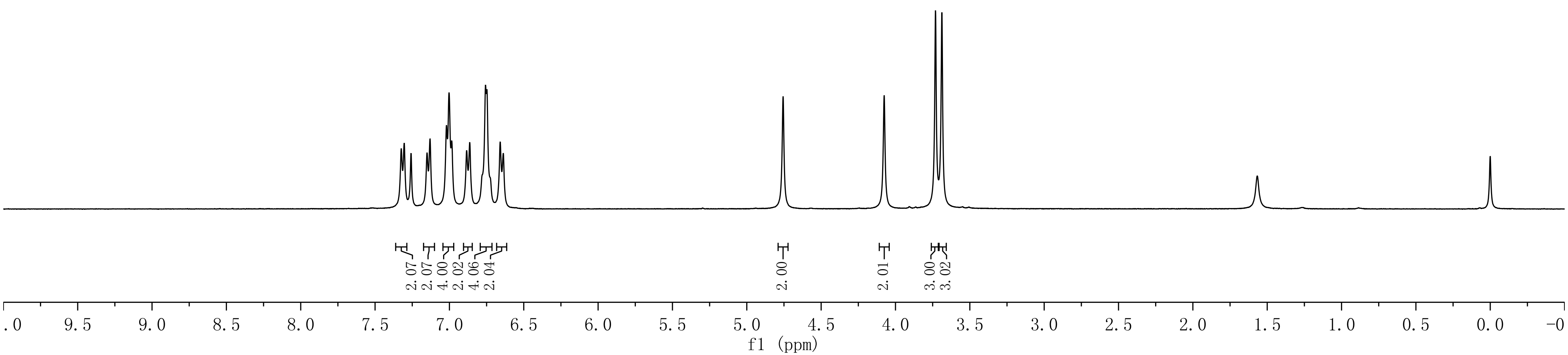
7.32
7.30
7.26
7.15
7.13
7.02
7.00
6.98
6.88
6.86
6.76
6.75
6.66
6.64
4.76
4.08
3.73
3.69
1.57
0.00
2.07
2.07
4.00
2.02
4.06
2.04
2.00
2.01
3.00
3.02
.0
9.5
9.0
8.5
8.0
7.5
7.0
6.5
6.0
5.5
5.0
4.5
4.0
3.5
3.0
2.5
2.0
1.5
1.0
0.5
0.0
-0
f1 (ppm)

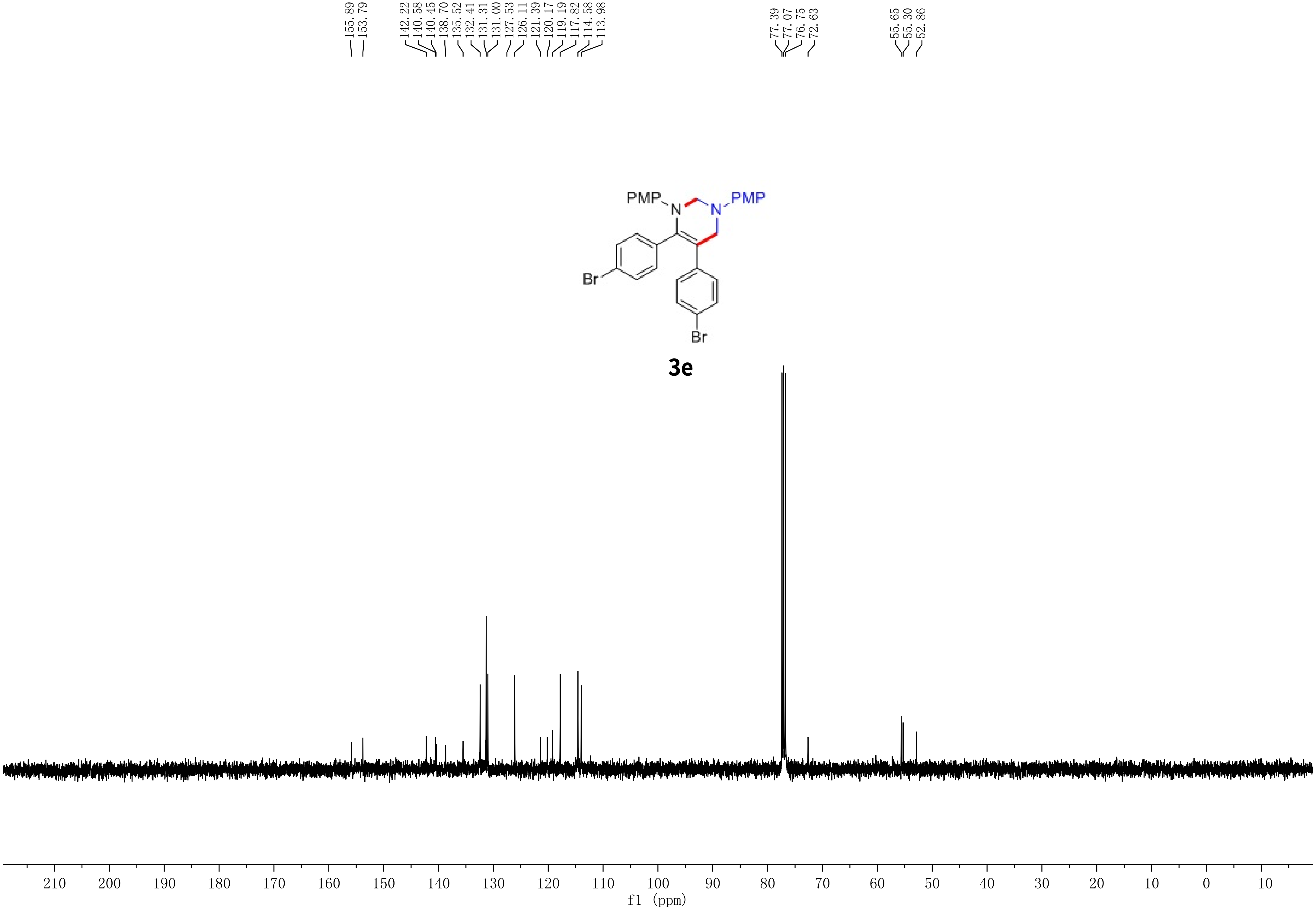
155.89
153.79
142.22
140.58
140.45
138.70
135.52
132.41
131.31
131.00
127.53
126.11
121.39
120.17
119.19
117.82
114.58
113.98
77.39
77.07
76.75
72.63
55.65
55.30
52.86
PMP
N
N
PMP
Br
Br
3e
210 200 190 180 170 160 150 140 130 120 110 100 90 80 70 60 50 40 30 20 10 0 −10
f1 (ppm)

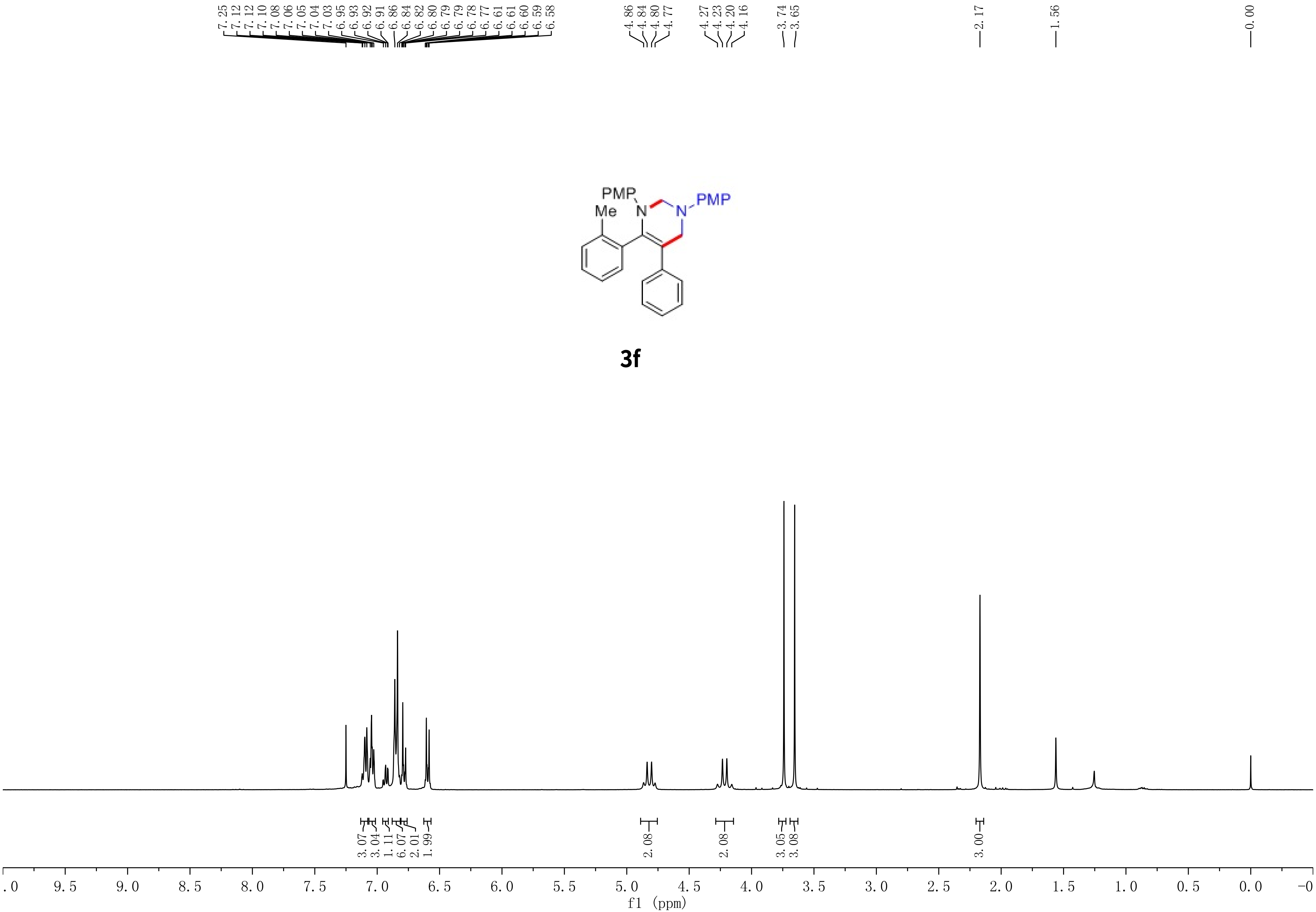
7.25 7.12 7.12 7.10 7.08 7.06 7.05 7.04 7.03 6.95 6.93 6.92 6.91 6.86 6.84 6.82 6.80 6.79 6.79 6.78 6.77 6.61 6.61 6.60 6.59 6.58
4.86 4.84 4.80 4.77
4.27 4.23 4.20 4.16
3.74 3.65
2.17
1.56
0.00
PMP
Me
N
N
PMP
3.07 3.04 1.11 6.07 2.01 1.99
2.08
2.08
3.05 3.08
3.00
.0 9.5 9.0 8.5 8.0 7.5 7.0 6.5 6.0 5.5 5.0 4.5 4.0 3.5 3.0 2.5 2.0 1.5 1.0 0.5 0.0 -0
f1 (ppm)

3f

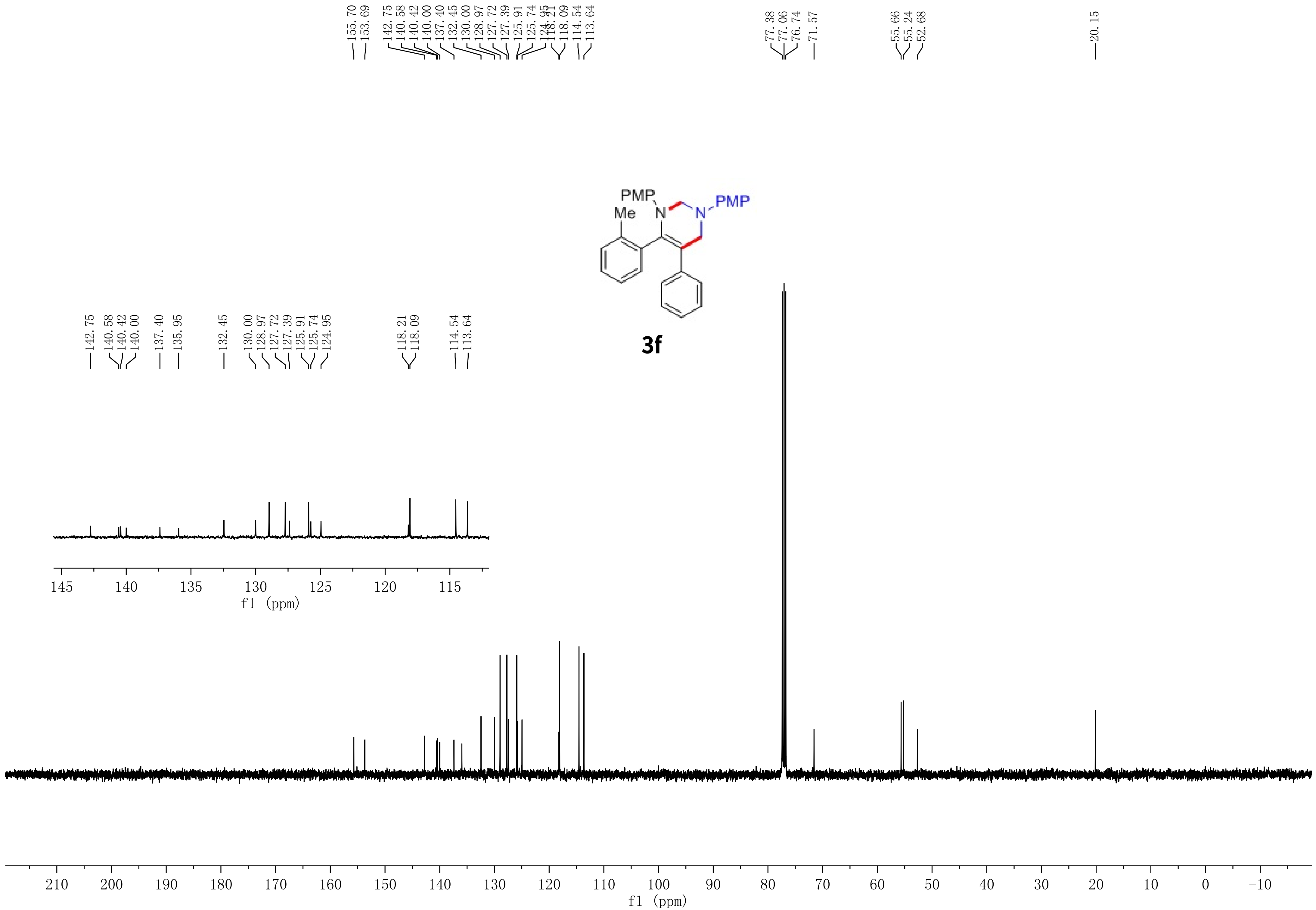
155.70
153.69
142.75
140.58
140.42
140.00
137.40
132.45
130.00
128.97
127.72
127.39
125.91
125.74
124.95
118.21
118.09
114.54
113.64
77.38
77.06
76.74
71.57
55.66
55.24
52.68
20.15
PMP
Me
N
N
PMP
3f
142.75
140.58
140.42
140.00
137.40
135.95
132.45
130.00
128.97
127.72
127.39
125.91
125.74
124.95
118.21
118.09
114.54
113.64
145 140 135 130 125 120 115
f1 (ppm)
210 200 190 180 170 160 150 140 130 120 110 100 90 80 70 60 50 40 30 20 10 0 -10
f1 (ppm)

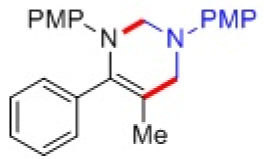
PMP
N
N
PMP
Me

3g

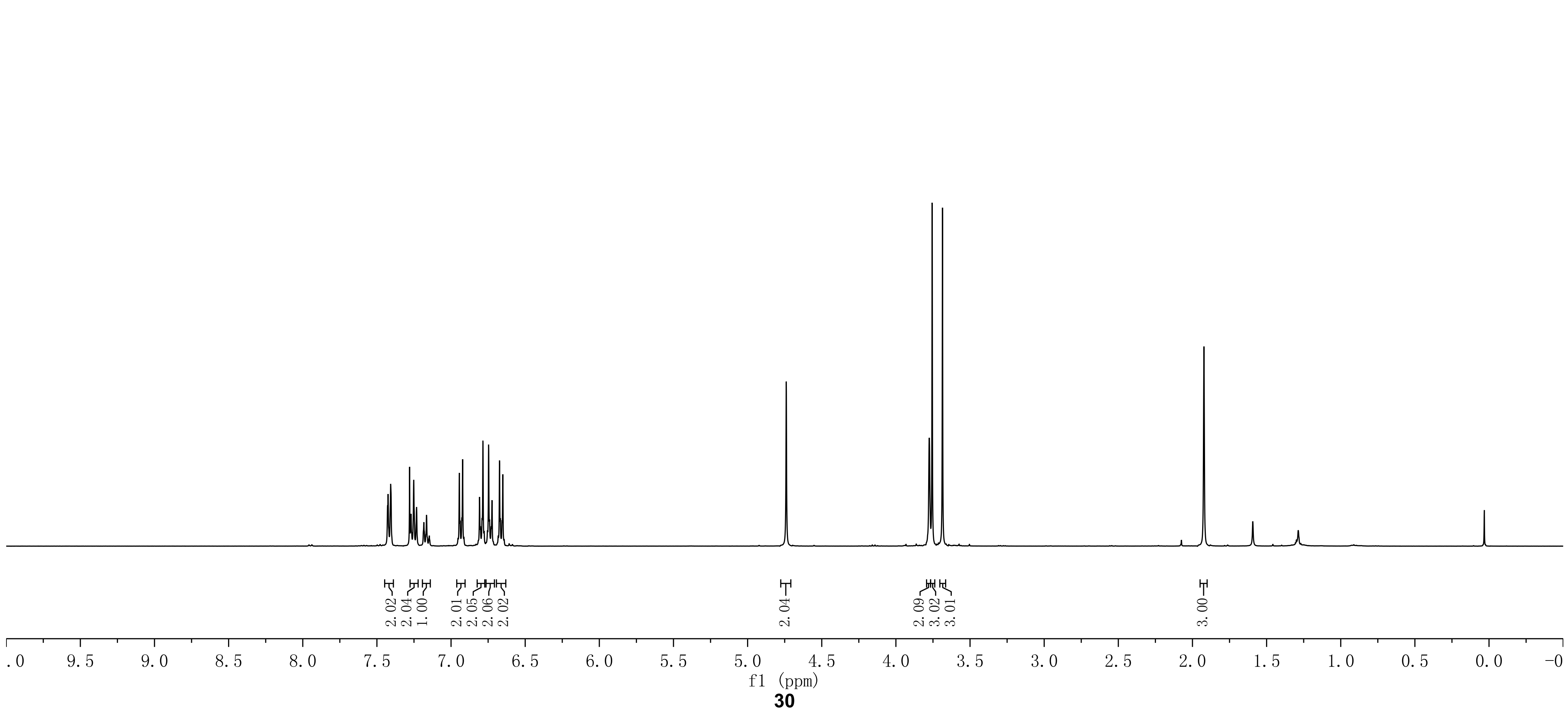
7.43
7.42
7.41
7.28
7.27
7.25
7.23
7.18
7.17
7.15
6.94
6.94
6.93
6.92
6.91
6.81
6.80
6.79
6.78
6.78
6.75
6.74
6.73
6.72
6.68
6.67
6.67
6.66
6.65
4.74
3.77
3.76
3.69
1.92
1.59
0.03
2.02
2.04
1.00
2.01
2.05
2.06
2.02
2.04
2.09
3.02
3.01
3.00
.0 9.5 9.0 8.5 8.0 7.5 7.0 6.5 6.0 5.5 5.0 4.5 4.0 3.5 3.0 2.5 2.0 1.5 1.0 0.5 0.0 -0
f1 (ppm)

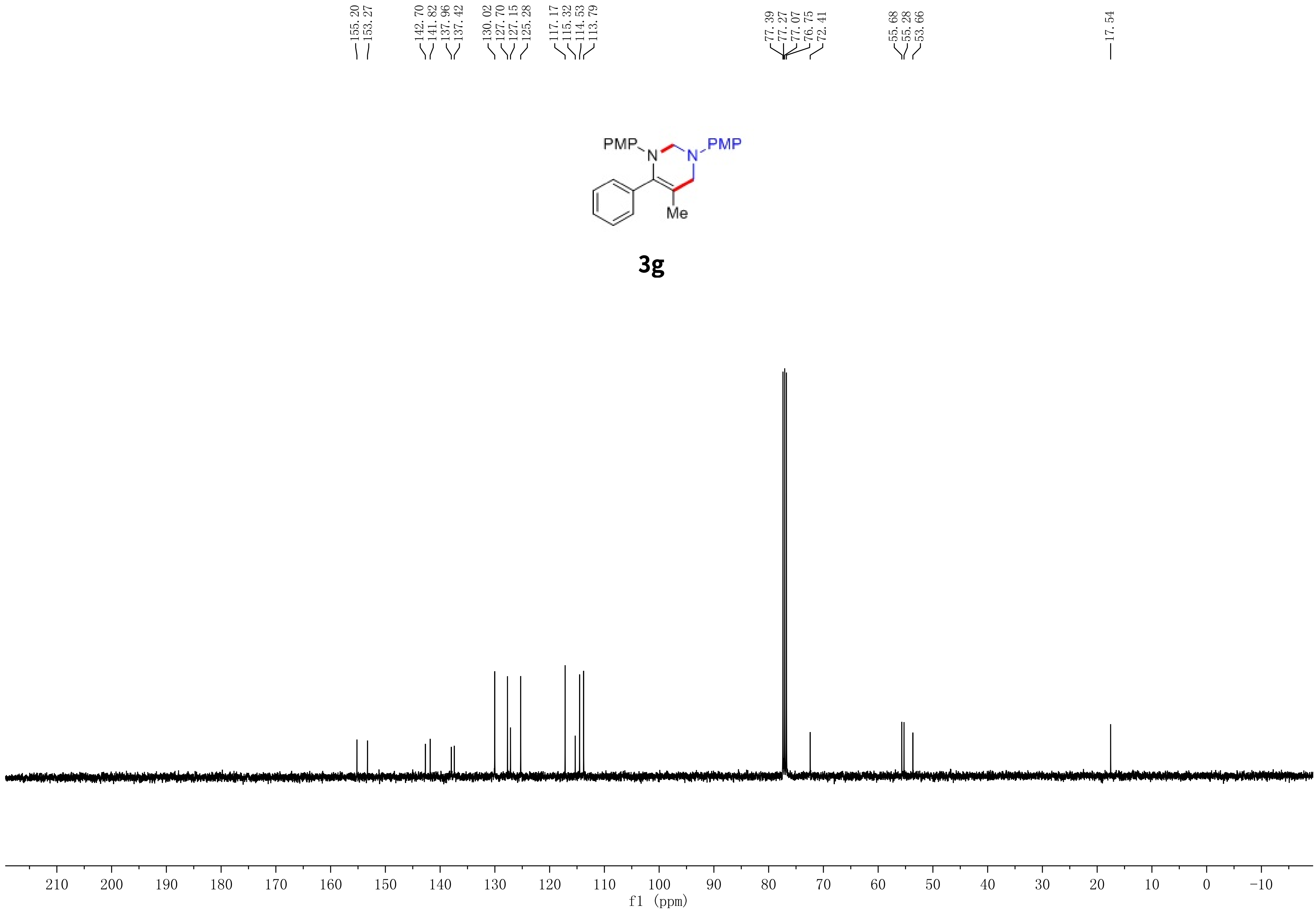

155.20
153.27
142.70
141.82
137.96
137.42
130.02
127.70
127.15
125.28
117.17
115.32
114.53
113.79
77.39
77.27
77.07
76.75
72.41
55.68
55.28
53.66
17.54
PMP
N
N
PMP
Me
3g
210 200 190 180 170 160 150 140 130 120 110 100 90 80 70 60 50 40 30 20 10 0 -10
f1 (ppm)

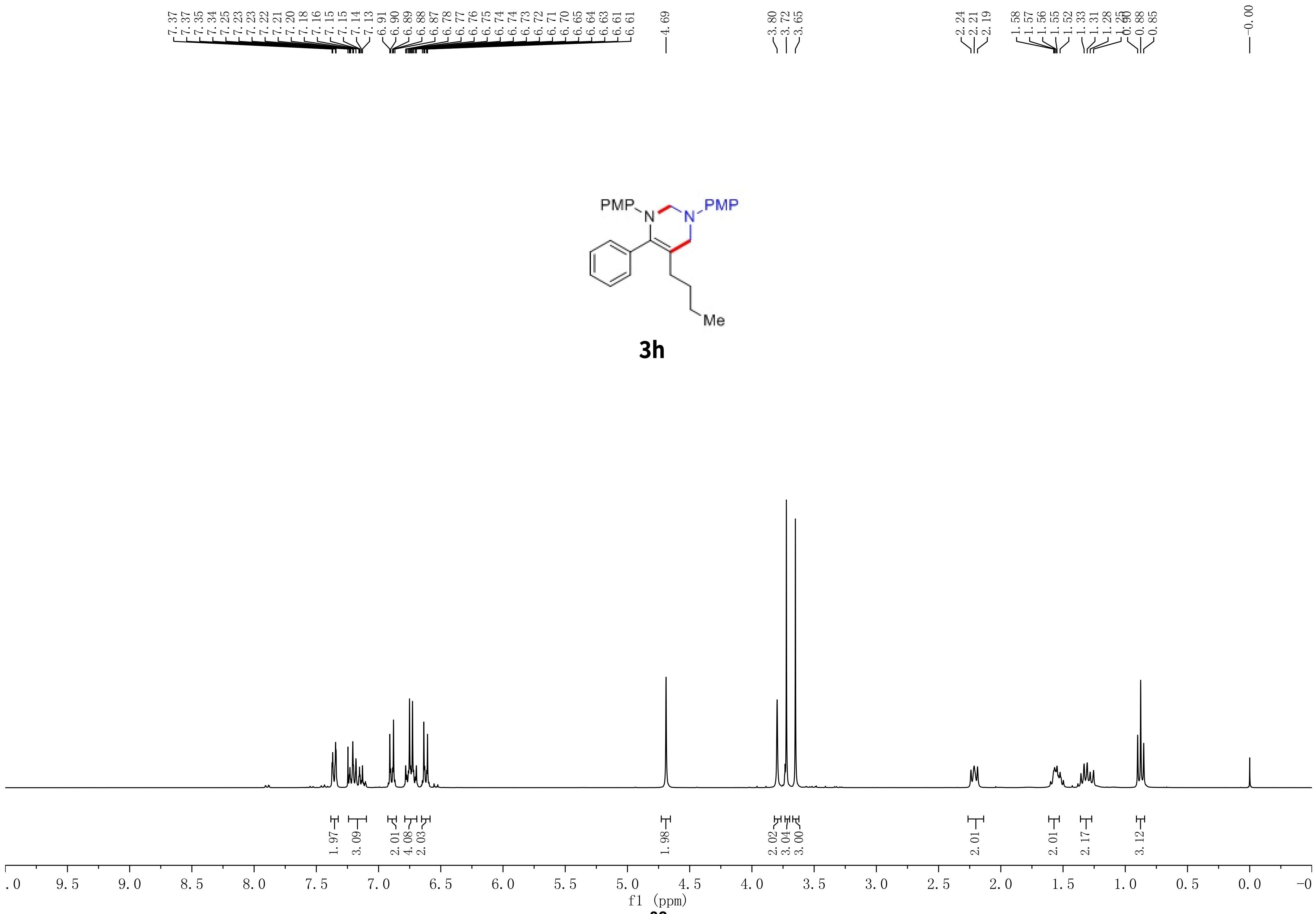
7.37 7.37 7.35 7.34 7.25 7.23 7.23 7.22 7.21 7.20 7.18 7.16 7.15 7.15 7.14 7.13 6.91 6.90 6.89 6.88 6.87 6.78 6.77 6.76 6.75 6.74 6.74 6.73 6.72 6.71 6.70 6.65 6.64 6.63 6.61 6.61
4.69
3.80 3.72 3.65
2.24 2.21 2.19
1.58 1.57 1.56 1.55 1.52 1.33 1.31 1.28 1.25 0.90 0.88 0.85
0.00
PMP
N
N
PMP
Me
3h
1.97 3.09 2.01 4.08 2.03 1.98 2.02 3.04 3.00 2.01 2.01 2.17 3.12
f1 (ppm)

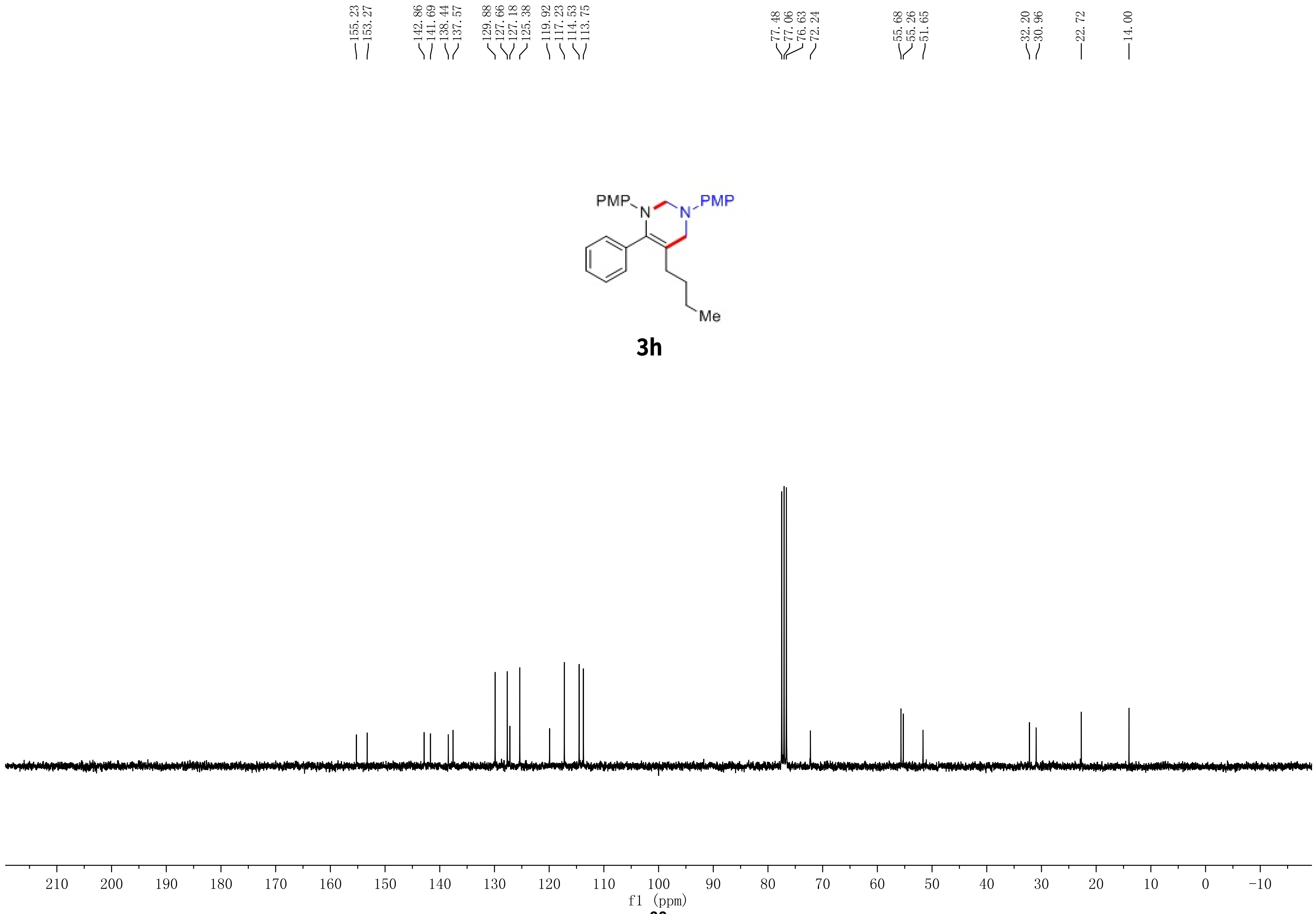
155.23
153.27
142.86
141.69
138.44
137.57
129.88
127.66
127.18
125.38
119.92
117.23
114.53
113.75
77.48
77.06
76.63
72.24
55.68
55.26
51.65
32.20
30.96
22.72
14.00
PMP
N
N
PMP
Me
3h
210 200 190 180 170 160 150 140 130 120 110 100 90 80 70 60 50 40 30 20 10 0 -10
f1 (ppm)

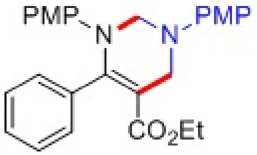
PMP
N
N
PMP
CO2Et

3i

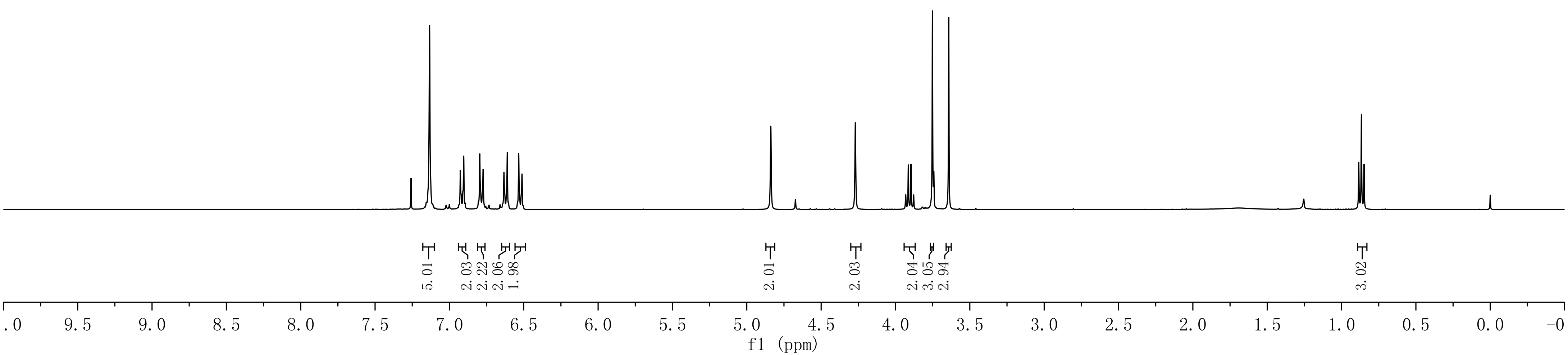
7.26
7.13
6.93
6.90
6.80
6.77
6.63
6.63
6.61
6.53
6.51
4.84
4.27
3.93
3.91
3.90
3.88
3.75
3.64
0.88
0.87
0.85
-0.00
5.01
2.03
2.22
2.06
1.98
2.01
2.03
2.04
3.05
2.94
3.02
f1 (ppm)

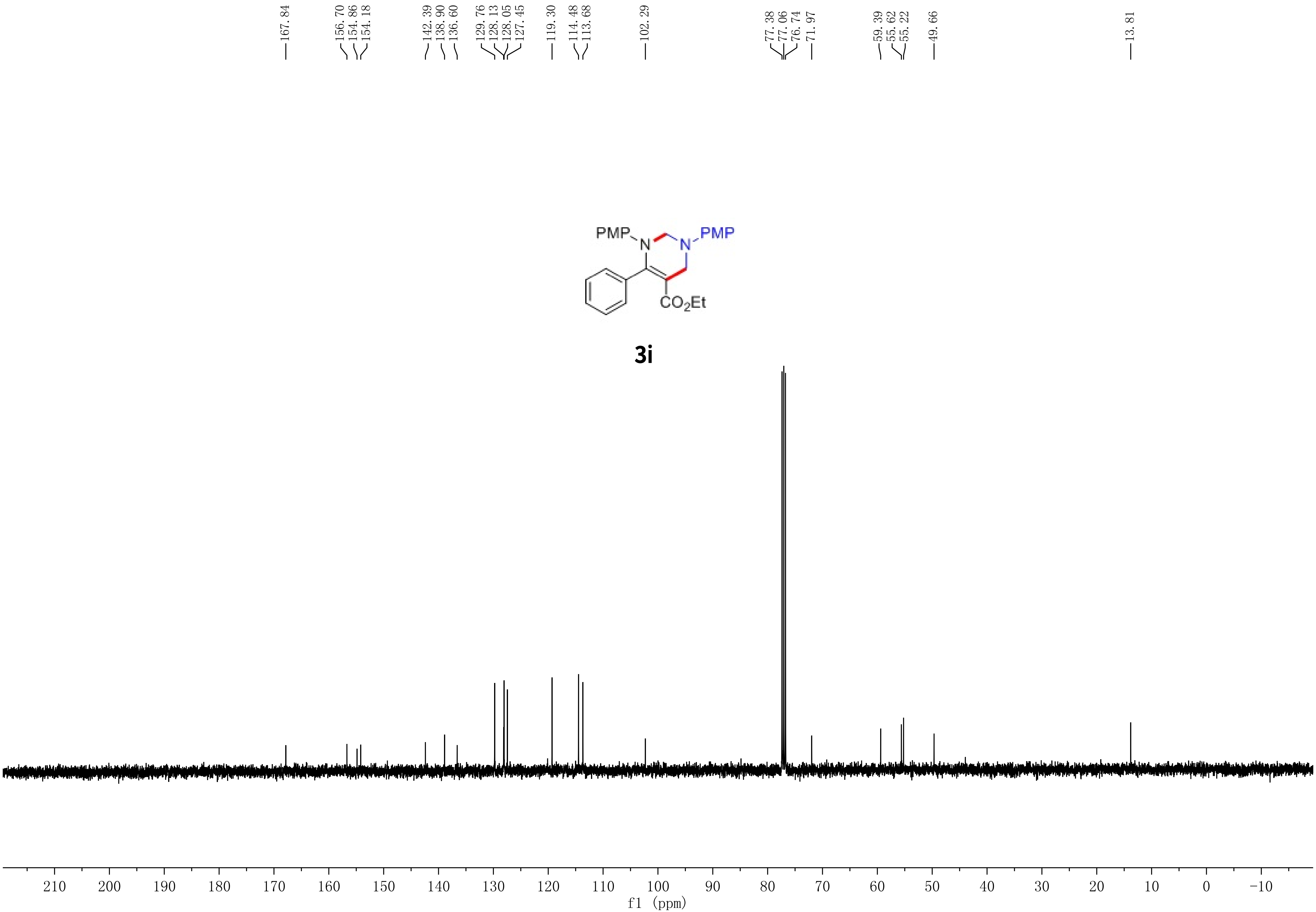

167.84
156.70
154.86
154.18
142.39
138.90
136.60
129.76
128.13
128.05
127.45
119.30
114.48
113.68
102.29
77.38
77.06
76.74
71.97
59.39
55.62
55.22
49.66
13.81
PMP
N
N
PMP
CO2Et
210 200 190 180 170 160 150 140 130 120 110 100 90 80 70 60 50 40 30 20 10 0 −10
f1 (ppm)

3i

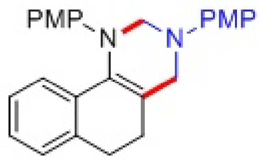
PMP
N
N
PMP

3j

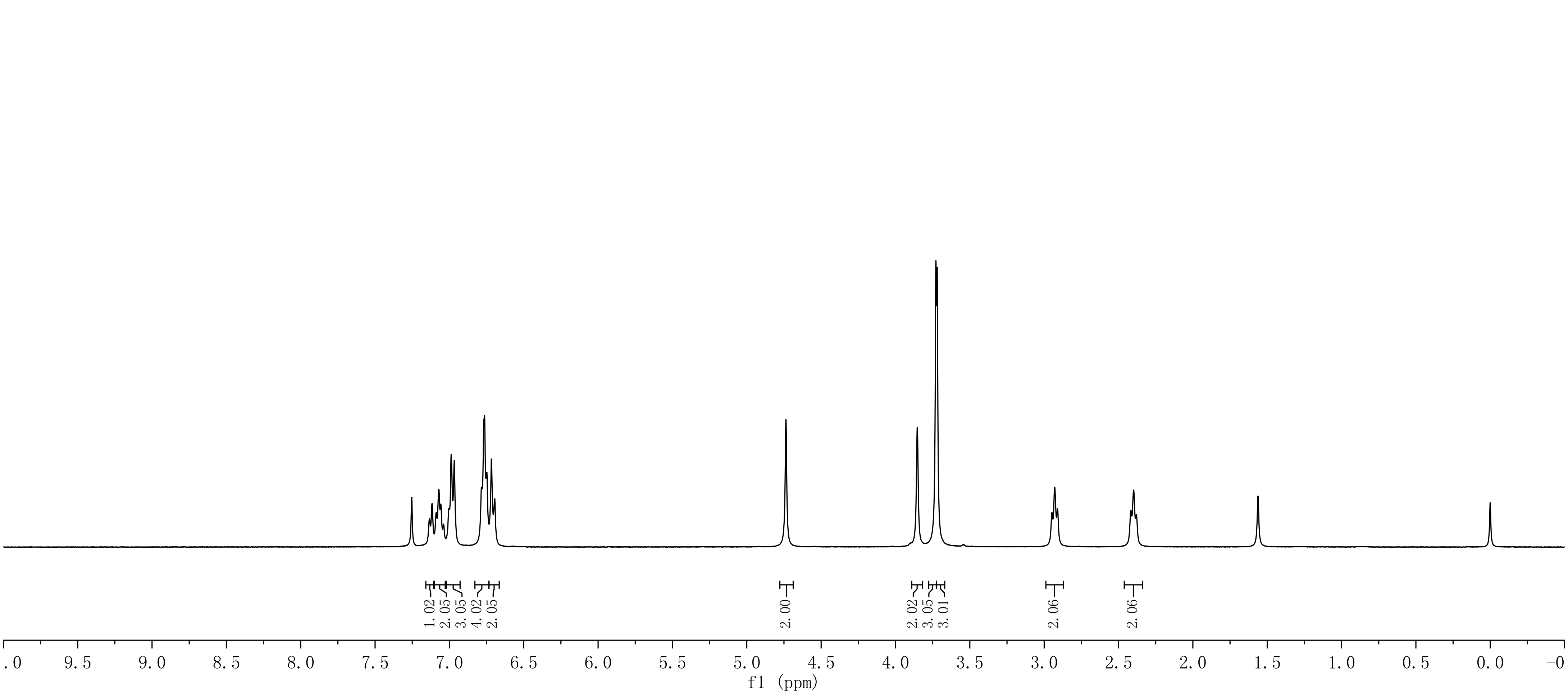
7.25
7.13
7.12
7.09
7.07
7.06
7.04
7.00
6.99
6.97
6.78
6.76
6.75
6.72
6.70
4.74
3.85
3.73
3.72
2.95
2.93
2.91
2.42
2.40
2.38
1.56
0.00
1.02
2.05
3.05
4.02
2.05
2.00
2.02
3.05
3.01
2.06
2.06
.0
9.5
9.0
8.5
8.0
7.5
7.0
6.5
6.0
5.5
5.0
4.5
4.0
3.5
3.0
2.5
2.0
1.5
1.0
0.5
0.0
-0
f1 (ppm)

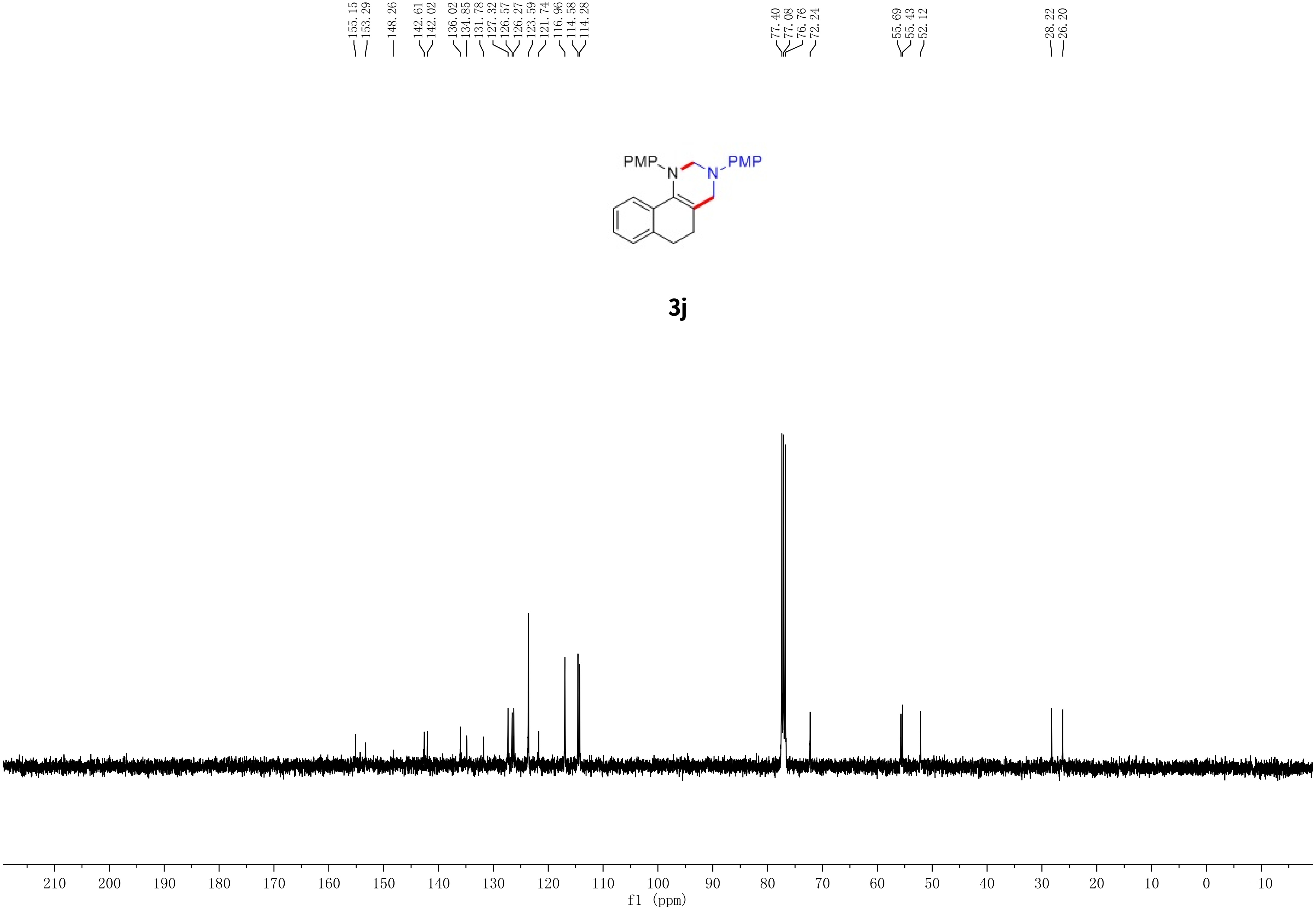
155.15
153.29
148.26
142.61
142.02
136.02
134.85
131.78
127.32
126.57
126.27
123.59
121.74
116.96
114.58
114.28
77.40
77.08
76.76
72.24
55.69
55.43
52.12
28.22
26.20
PMP
N
N
PMP
210
200
190
180
170
160
150
140
130
120
110
100
90
80
70
60
50
40
30
20
10
0
-10
f1 (ppm)

3j

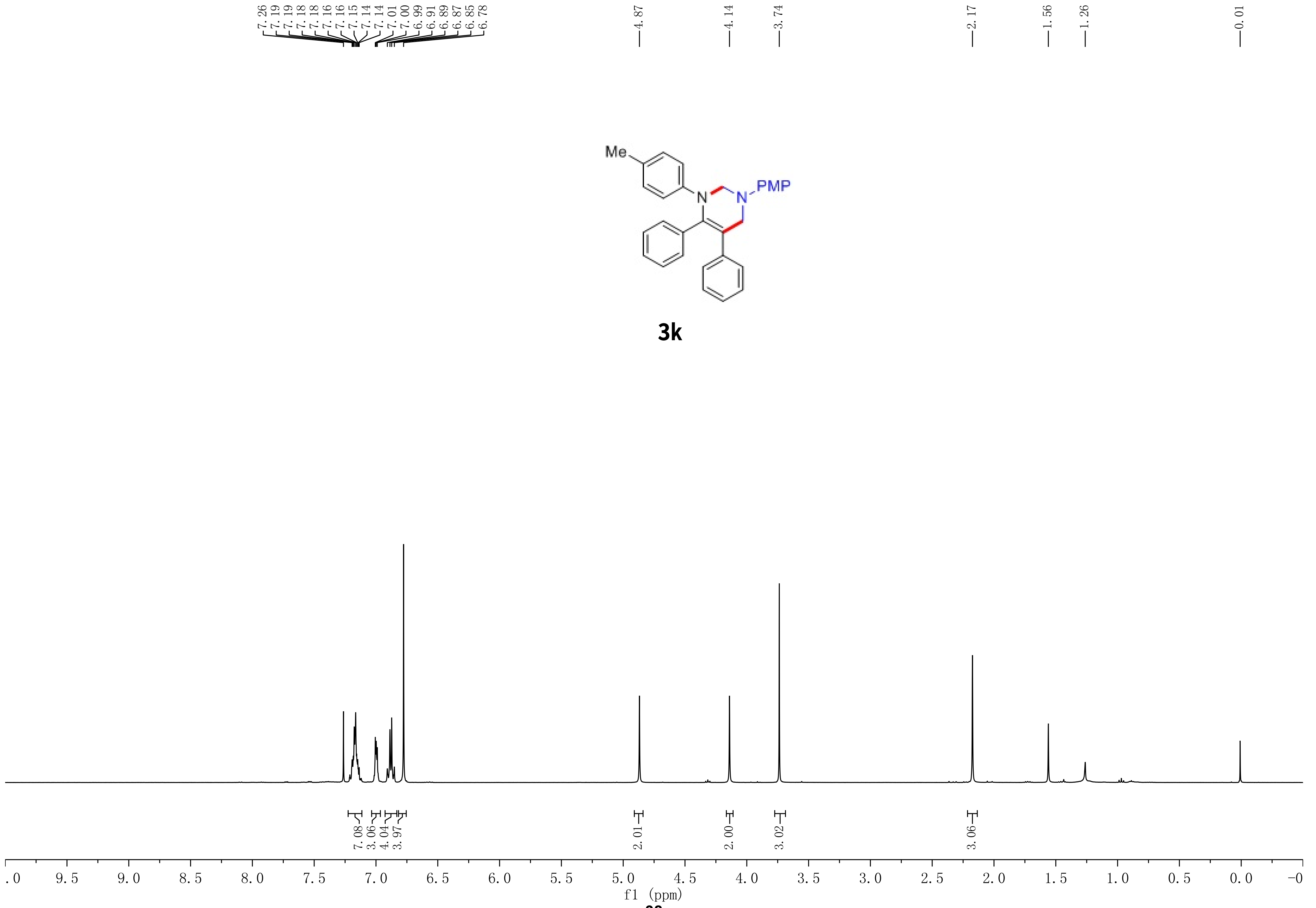
7.26
7.19
7.19
7.18
7.18
7.16
7.15
7.14
7.14
7.01
7.00
6.99
6.91
6.89
6.87
6.85
6.78
4.87
4.14
3.74
2.17
1.56
1.26
0.01
Me
N
N
PMP
3k
7.08
3.06
4.04
3.97
2.01
2.00
3.02
3.06
f1 (ppm)

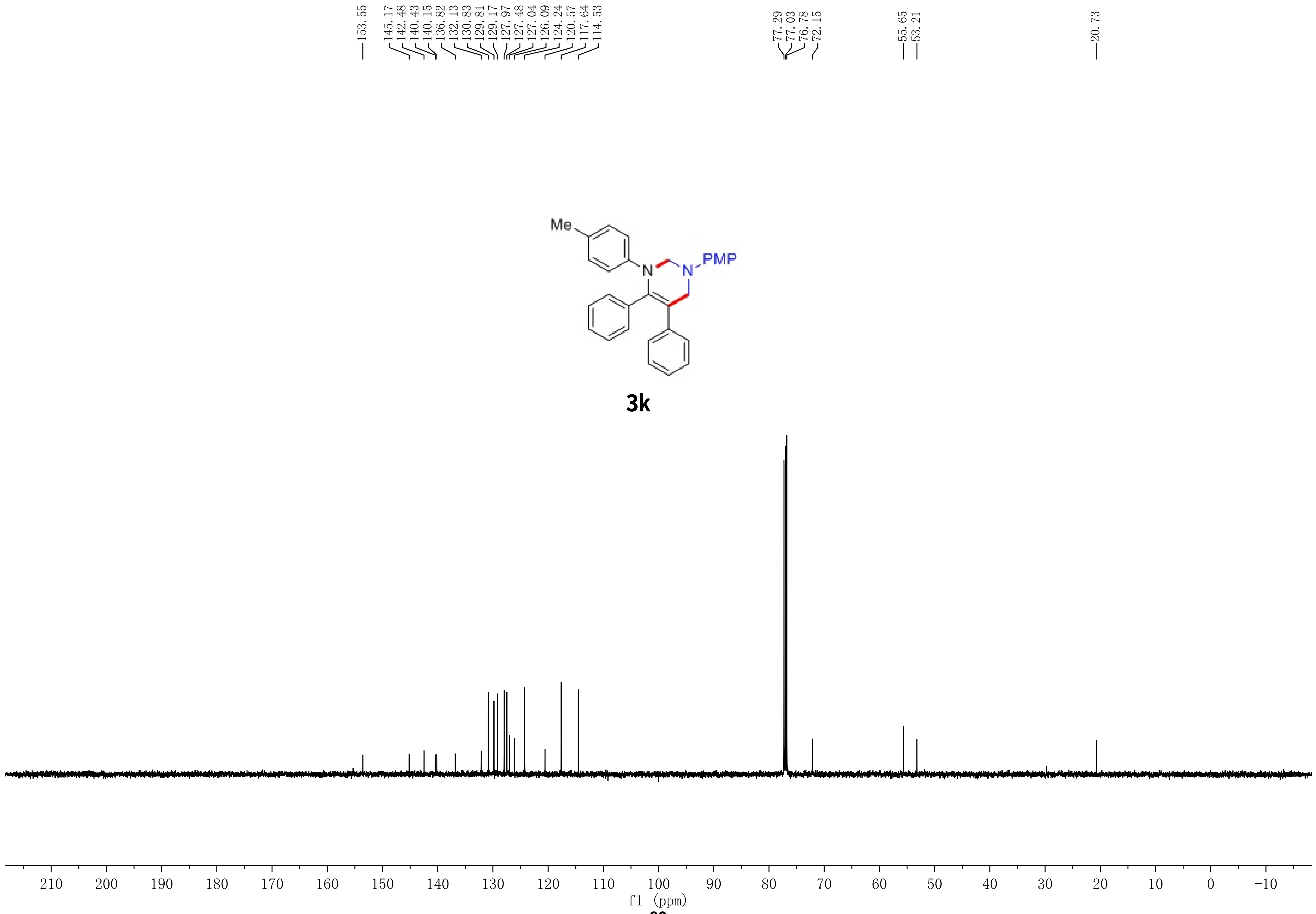

153.55
145.17
142.48
140.43
140.15
136.82
132.13
130.83
129.81
129.17
127.97
127.48
127.04
126.09
124.24
120.57
117.64
114.53
77.29
77.03
76.78
72.15
55.65
53.21
20.73
Me
N
N
PMP
210 200 190 180 170 160 150 140 130 120 110 100 90 80 70 60 50 40 30 20 10 0 −10
f1 (ppm)

3k

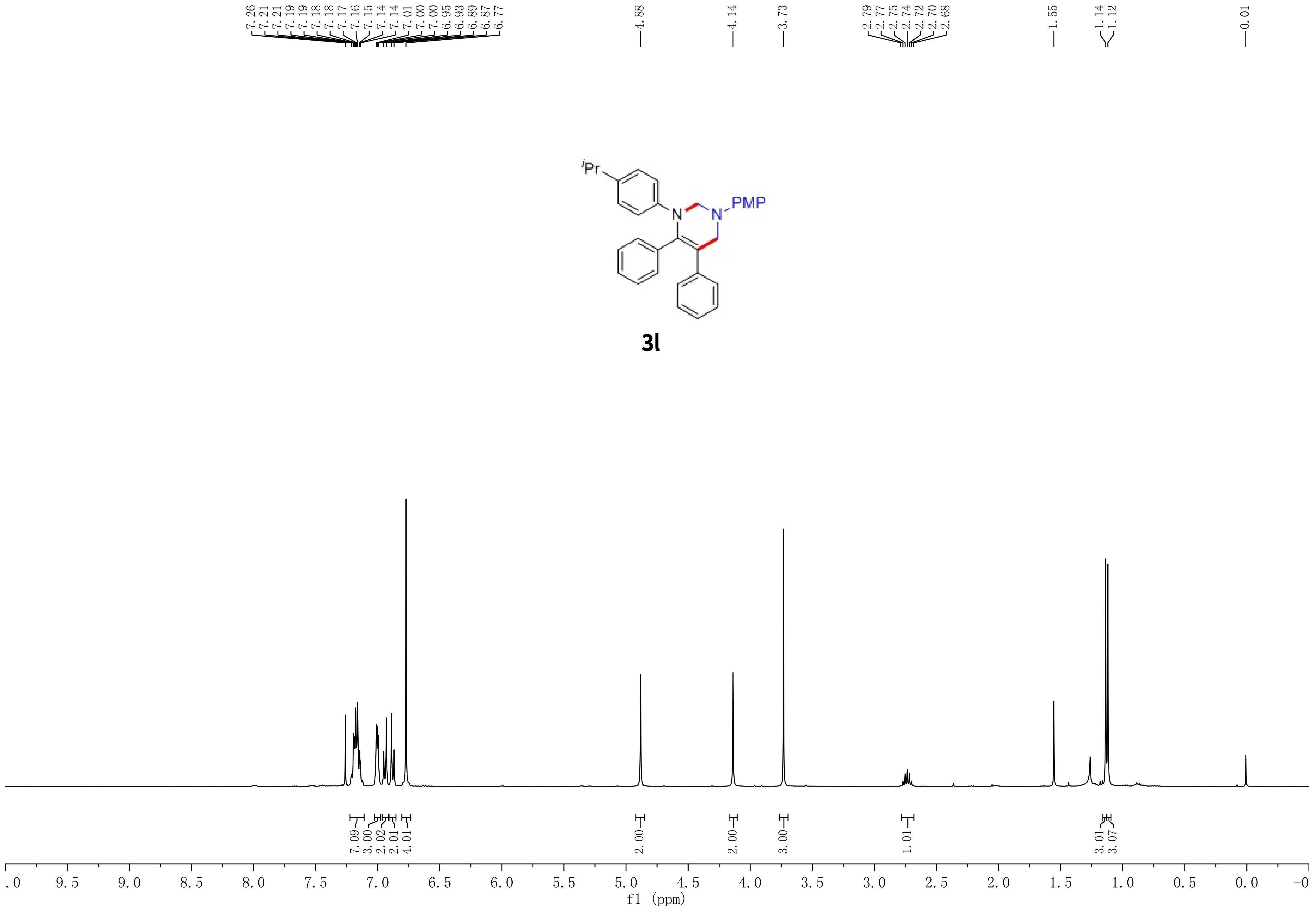
7.26 7.21 7.21 7.19 7.19 7.18 7.18 7.17 7.16 7.15 7.14 7.14 7.01 7.00 6.95 6.93 6.89 6.87 6.77
4.88
4.14
3.73
2.79 2.77 2.75 2.74 2.72 2.70 2.68
1.55
1.14 1.12
0.01
iPr
N
N
PMP
3l
7.09 3.00 2.02 2.01 4.01
2.00
2.00
3.00
1.01
3.01 3.07
f1 (ppm)

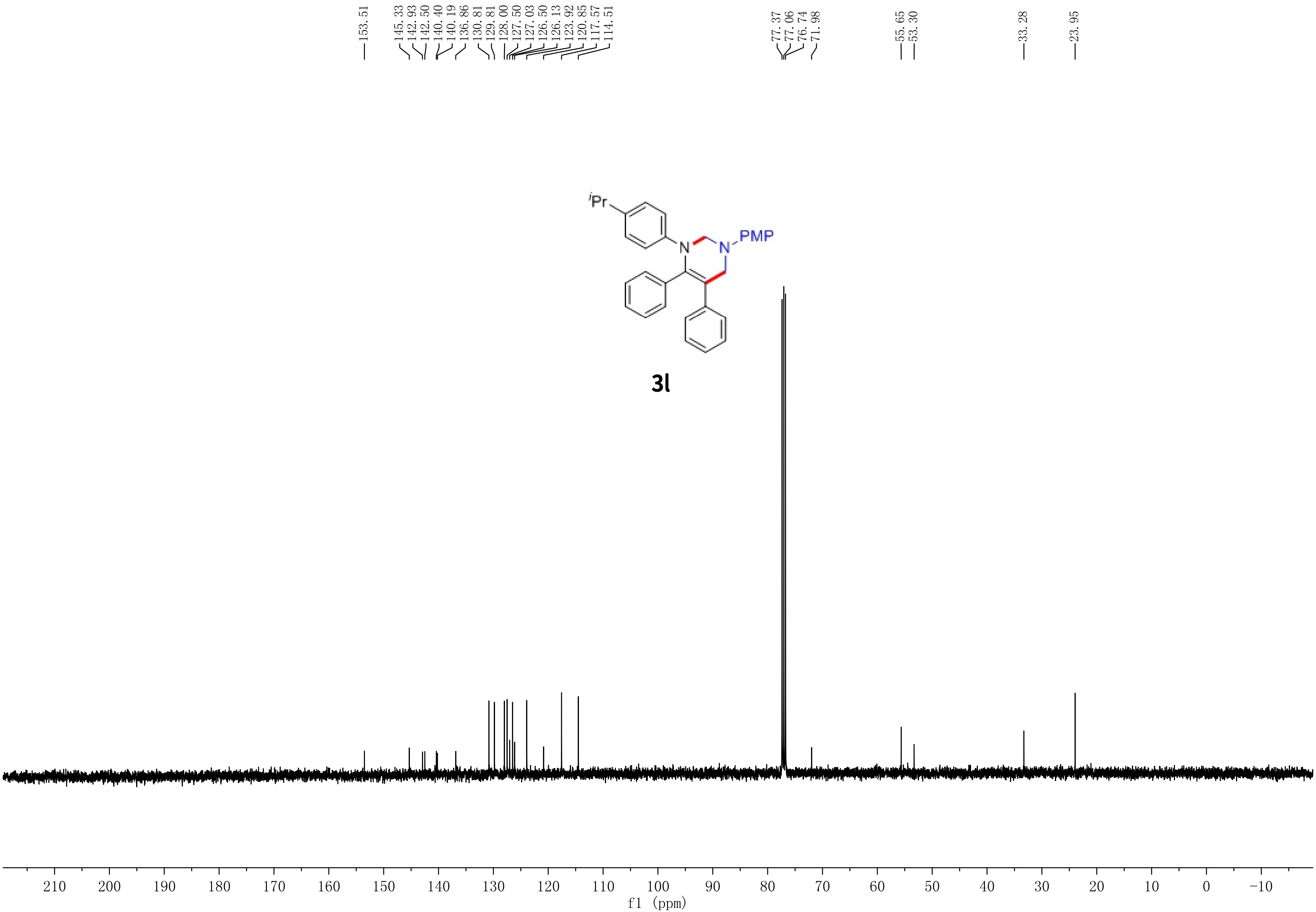
153.51
145.33
142.93
142.50
140.40
140.19
136.86
130.81
129.81
128.00
127.50
127.03
126.50
126.13
123.92
120.85
117.57
114.51
77.37
77.06
76.74
71.98
55.65
53.30
33.28
23.95
iPr
N
N
PMP
3l
210 200 190 180 170 160 150 140 130 120 110 100 90 80 70 60 50 40 30 20 10 0 -10
f1 (ppm)

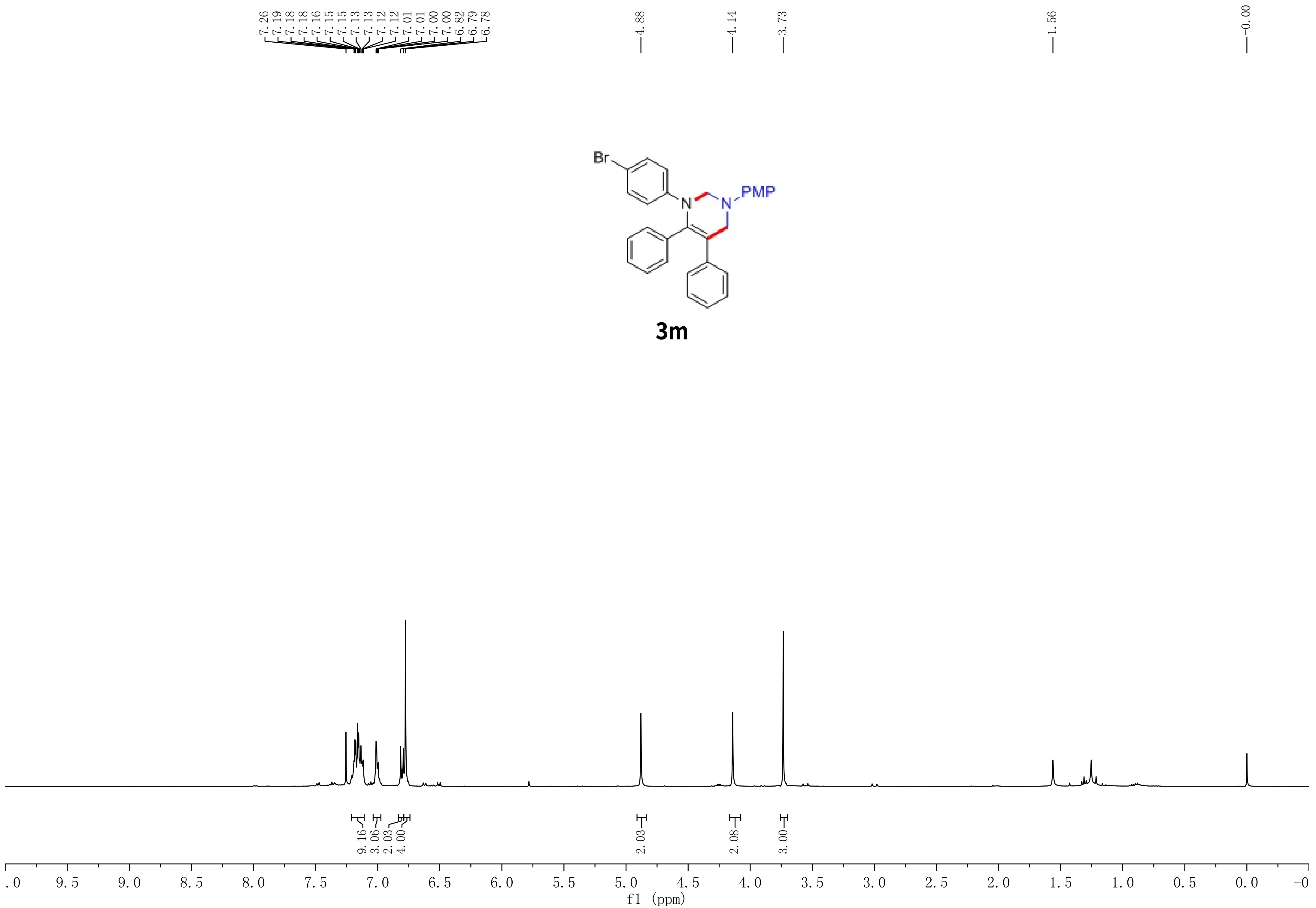
7.26
7.19
7.18
7.18
7.16
7.15
7.15
7.13
7.13
7.12
7.12
7.01
7.01
7.00
7.00
6.82
6.79
6.78
4.88
4.14
3.73
1.56
-0.00
Br
N
N
PMP
9.16
3.06
2.03
4.00
2.03
2.08
3.00
f1 (ppm)

3m

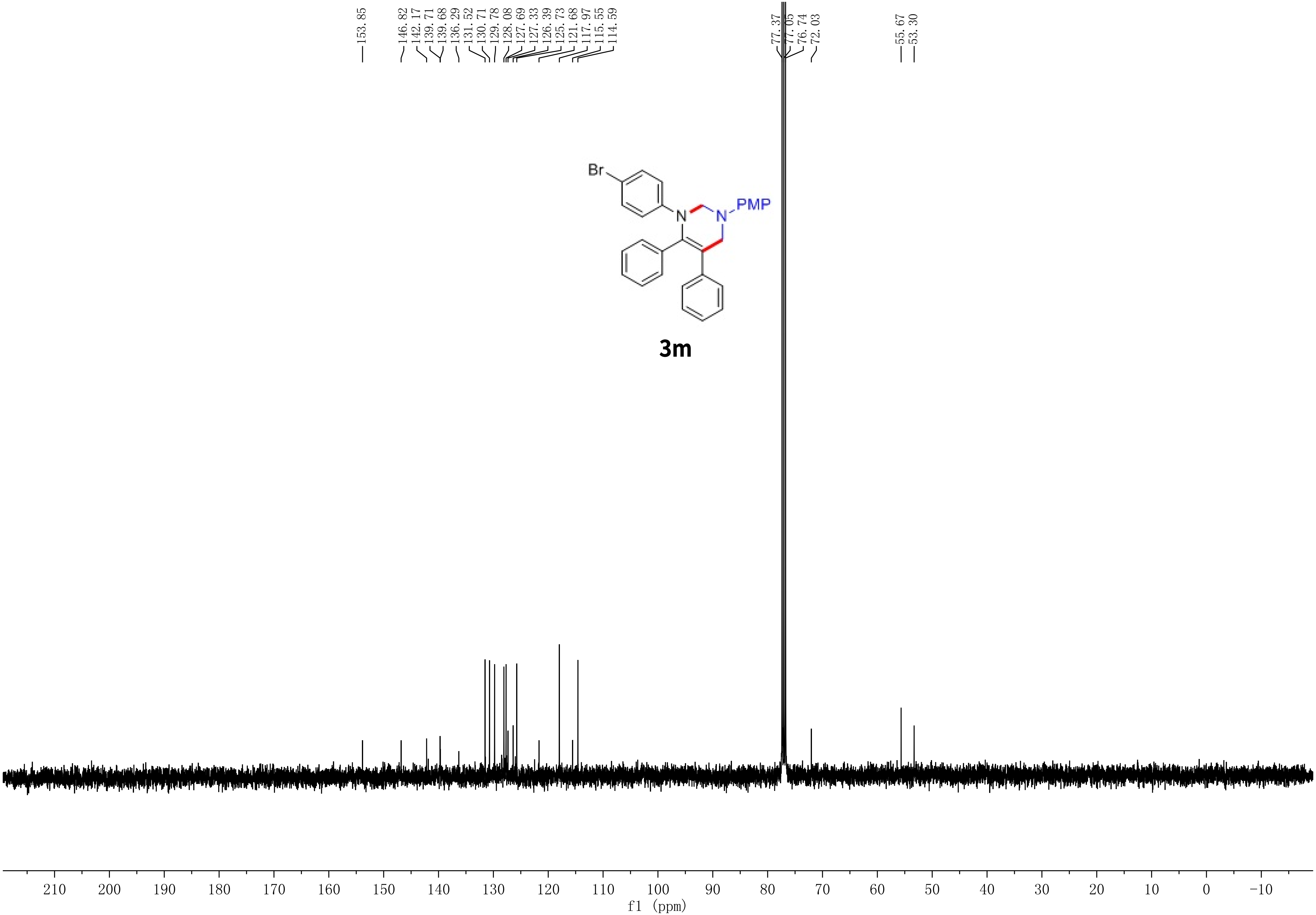
153.85
146.82
142.17
139.71
139.68
136.29
131.52
130.71
129.78
128.08
127.69
127.33
126.39
125.73
121.68
117.97
115.55
114.59
77.37
77.05
76.74
72.03
55.67
53.30
Br
N
N
PMP
3m
210 200 190 180 170 160 150 140 130 120 110 100 90 80 70 60 50 40 30 20 10 0 -10
f1 (ppm)

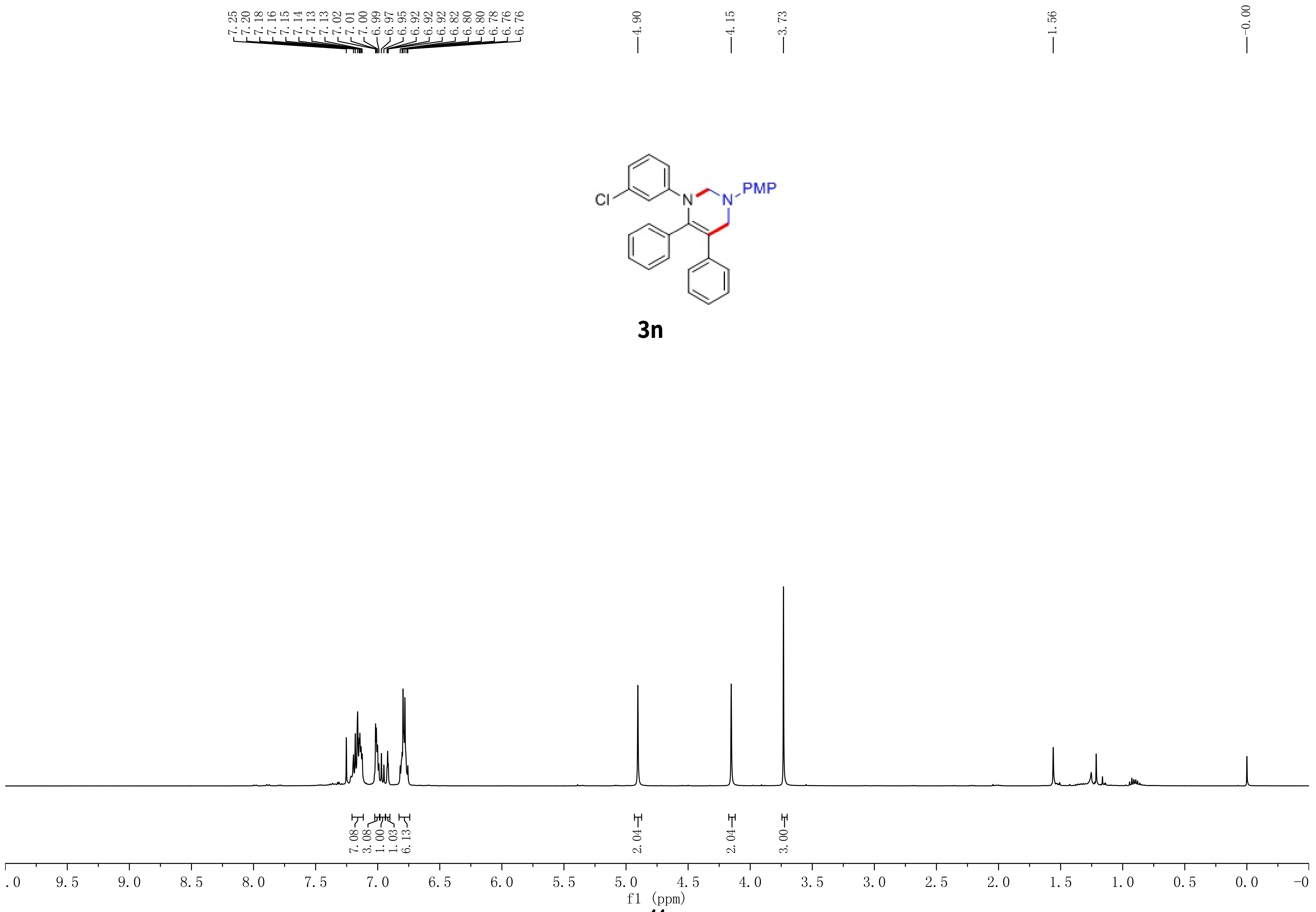
7.25
7.20
7.18
7.16
7.15
7.14
7.13
7.13
7.02
7.01
7.00
6.99
6.97
6.95
6.92
6.92
6.92
6.82
6.80
6.80
6.78
6.76
6.76
4.90
4.15
3.73
1.56
0.00
Cl
N
N
PMP
3n
7.08
3.08
1.00
1.03
6.13
2.04
2.04
3.00
f1 (ppm)

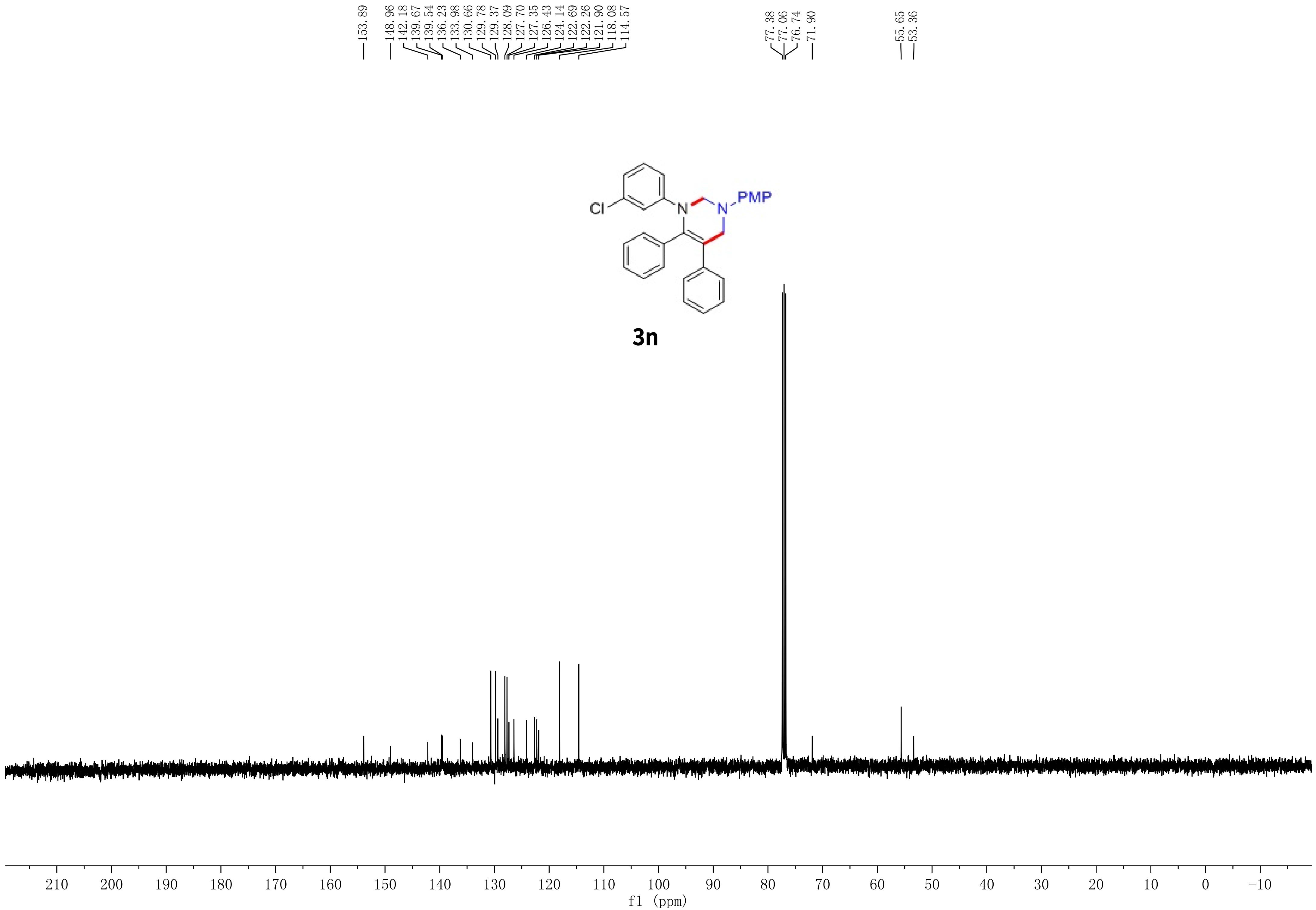
153.89
148.96
142.18
139.67
139.54
136.23
133.98
130.66
129.78
129.37
128.09
127.70
127.35
126.43
124.14
122.69
122.26
121.90
118.08
114.57
77.38
77.06
76.74
71.90
55.65
53.36
Cl
N
N
PMP
3n
210
200
190
180
170
160
150
140
130
120
110
100
90
80
70
60
50
40
30
20
10
0
-10
f1 (ppm)

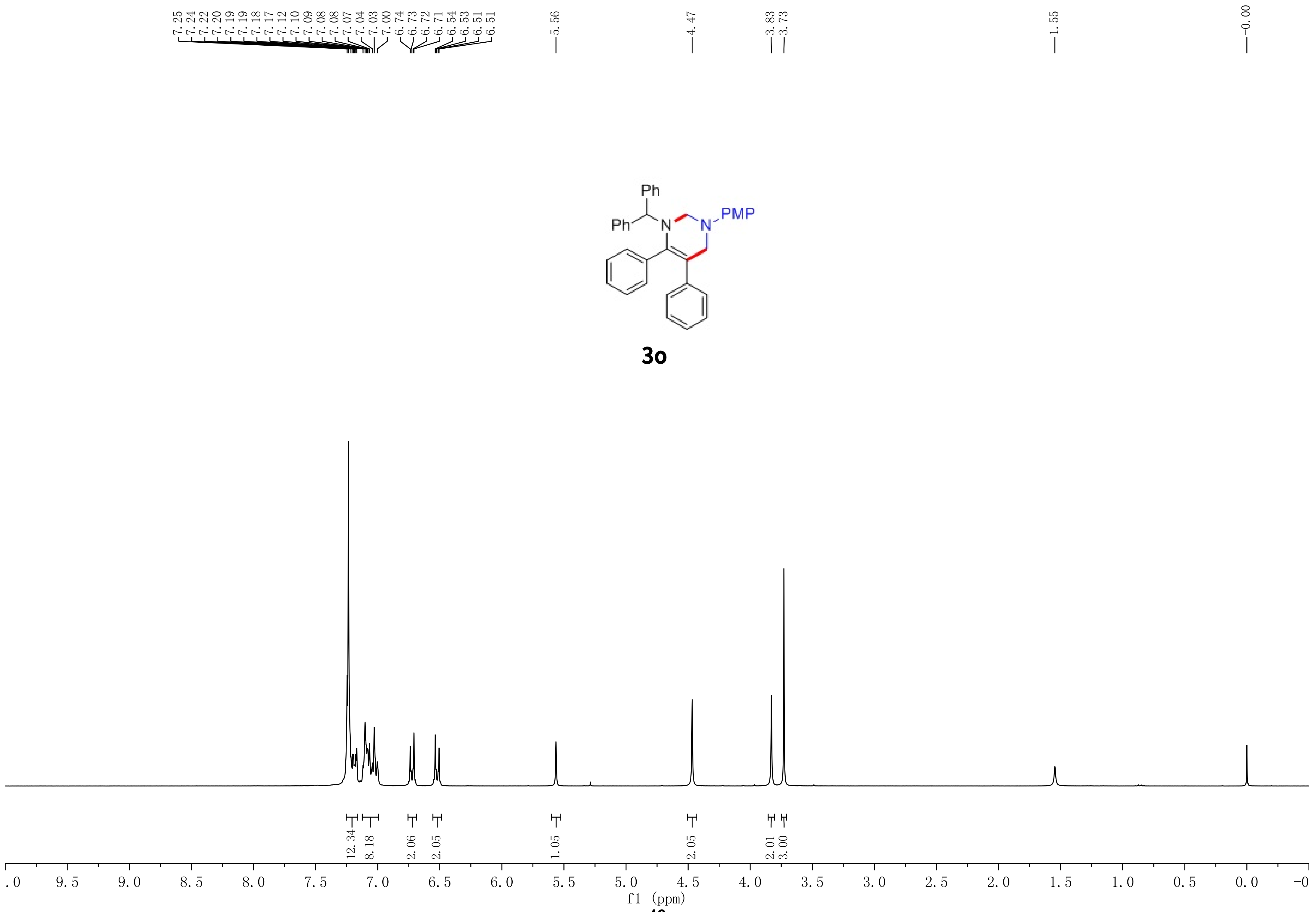
7.25
7.24
7.22
7.20
7.19
7.19
7.18
7.17
7.12
7.10
7.09
7.08
7.08
7.07
7.04
7.03
7.00
6.74
6.73
6.72
6.71
6.54
6.53
6.51
6.51
5.56
4.47
3.83
3.73
1.55
-0.00
Ph
Ph
N
N
PMP
3o
12.34
8.18
2.06
2.05
1.05
2.05
2.01
3.00
f1 (ppm)

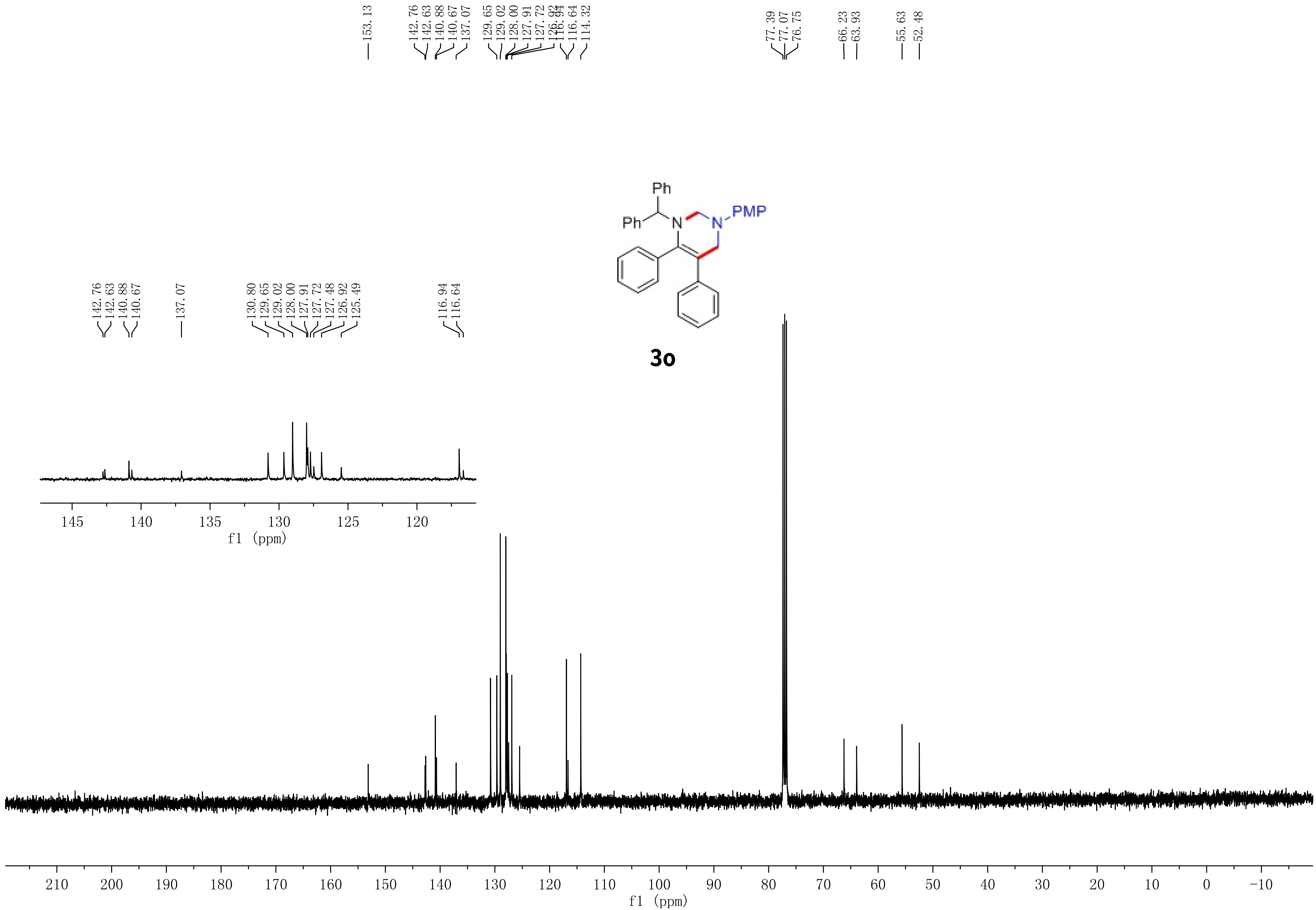
153.13
142.76
142.63
140.88
140.67
137.07
129.65
129.02
128.00
127.91
127.72
126.92
116.94
116.64
114.32
77.39
77.07
76.75
66.23
63.93
55.63
52.48
Ph
Ph
N
N
PMP
3o
142.76
142.63
140.88
140.67
137.07
130.80
129.65
129.02
128.00
127.91
127.72
127.48
126.92
125.49
116.94
116.64
145 140 135 130 125 120
f1 (ppm)
210 200 190 180 170 160 150 140 130 120 110 100 90 80 70 60 50 40 30 20 10 0 -10
f1 (ppm)

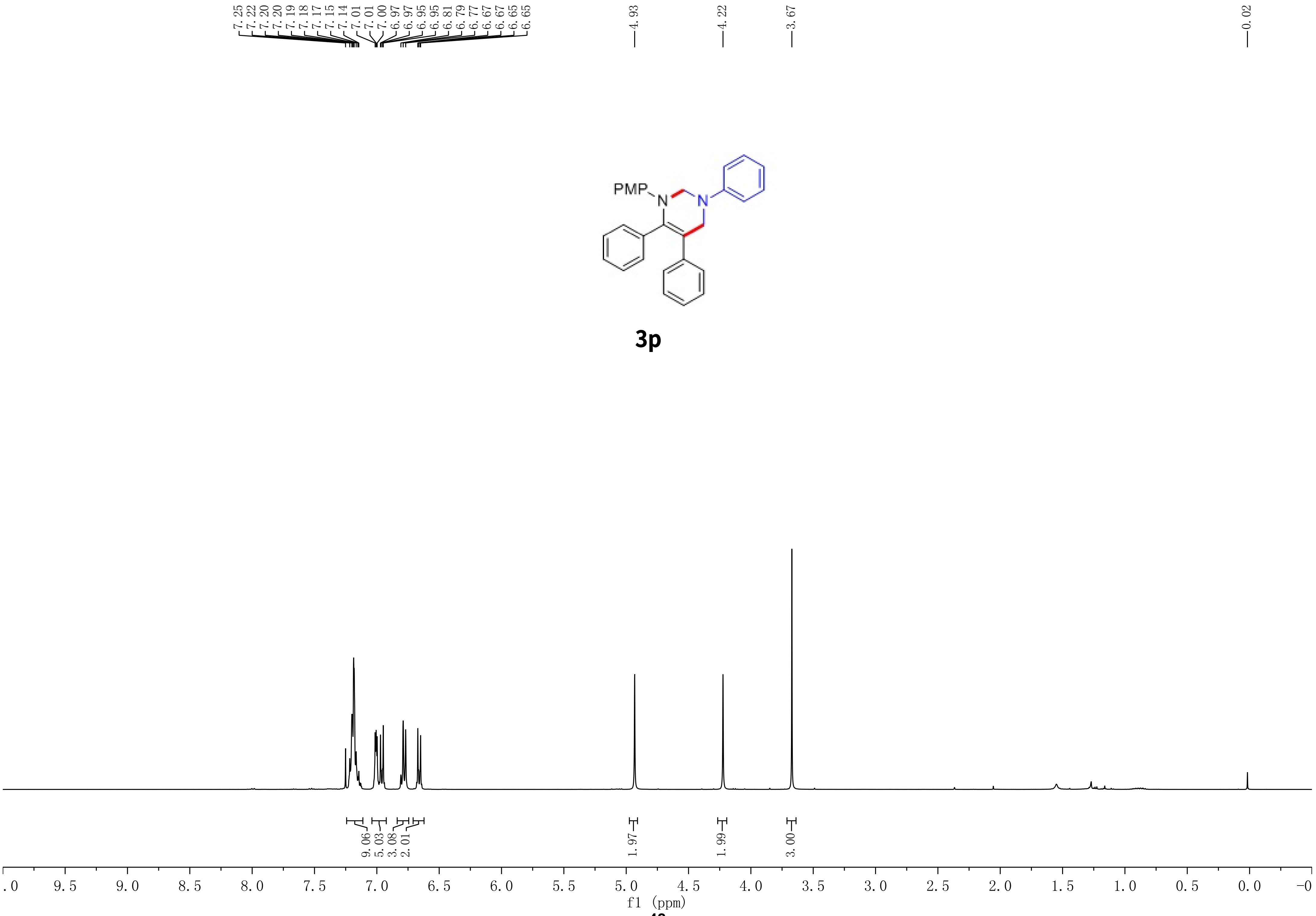
7.25
7.22
7.20
7.20
7.19
7.18
7.17
7.15
7.14
7.01
7.01
7.00
6.97
6.97
6.95
6.95
6.81
6.79
6.77
6.67
6.67
6.65
6.65
4.93
4.22
3.67
0.02
9.06
5.03
3.08
2.01
1.97
1.99
3.00
. 0
9.5
9.0
8.5
8.0
7.5
7.0
6.5
6.0
5.5
5.0
4.5
4.0
3.5
3.0
2.5
2.0
1.5
1.0
0.5
0.0
−0
f1 (ppm)
PMP
N
N

3p

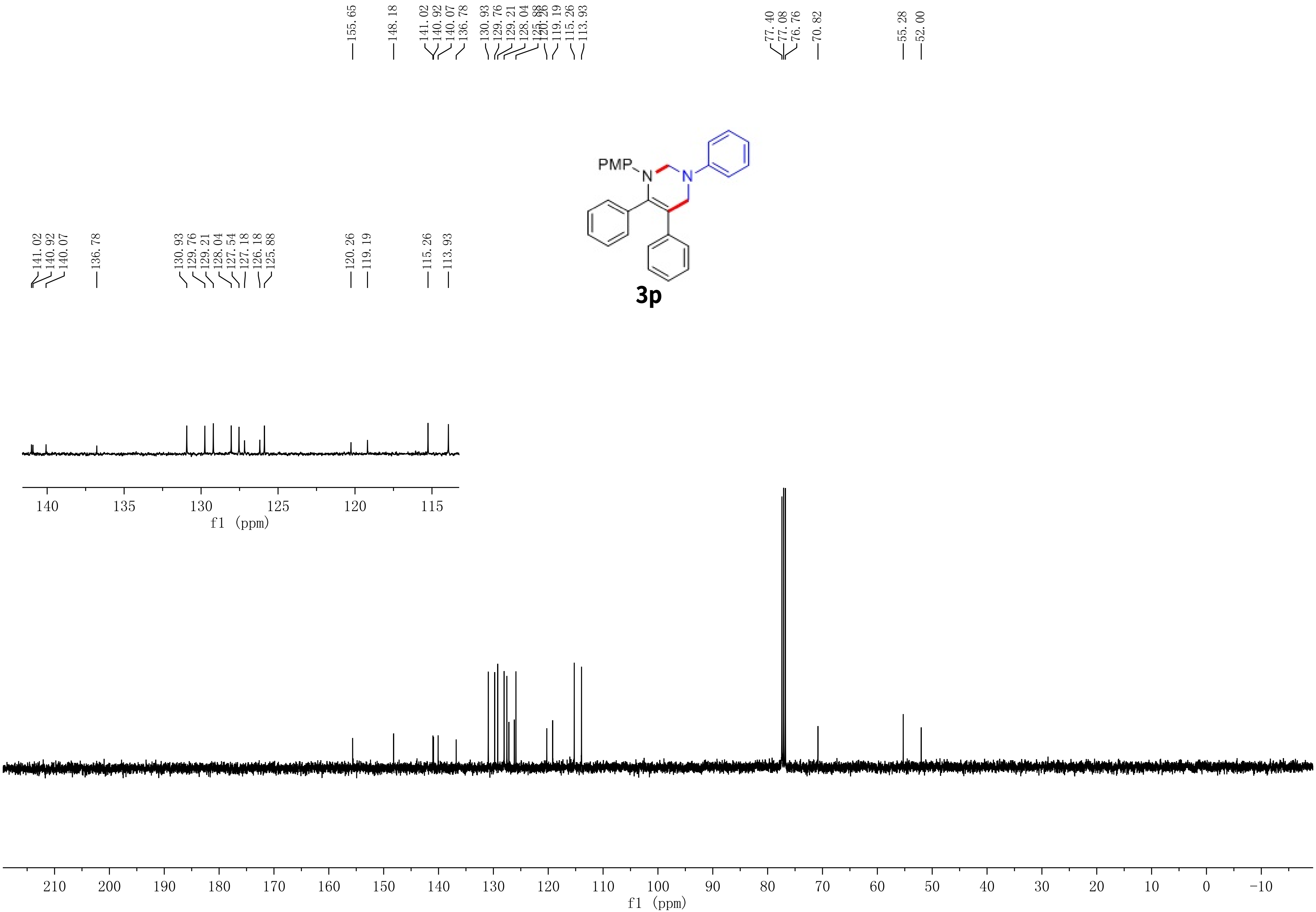
155.65
148.18
141.02
140.92
140.07
136.78
130.93
129.76
129.21
128.04
127.54
127.18
126.18
125.88
120.26
119.19
115.26
113.93
77.40
77.08
76.76
70.82
55.28
52.00
PMP
N
N
3p
140 135 130 125 120 115
f1 (ppm)
210 200 190 180 170 160 150 140 130 120 110 100 90 80 70 60 50 40 30 20 10 0 -10
f1 (ppm)

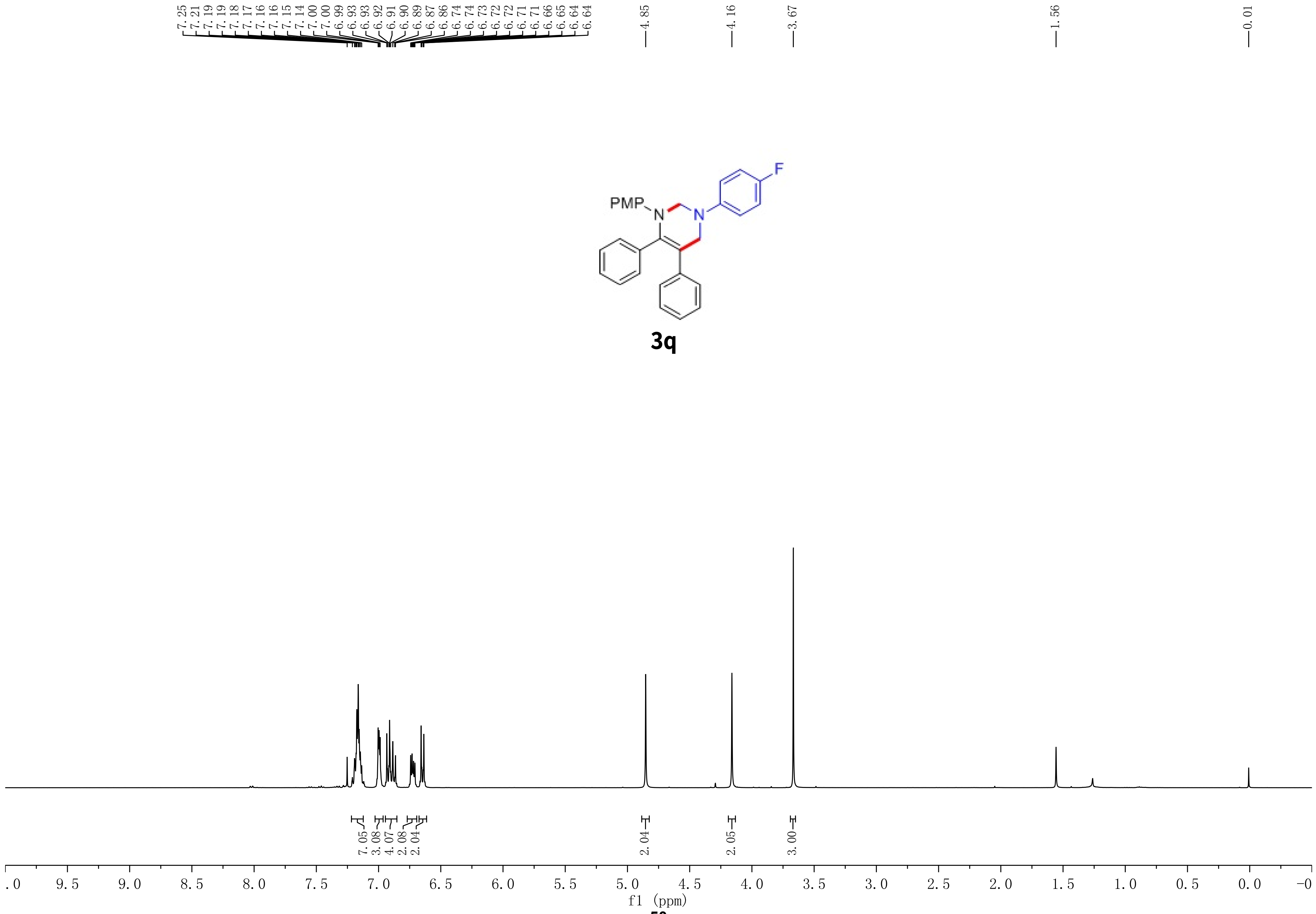
7.25 7.21 7.19 7.19 7.18 7.17 7.16 7.16 7.15 7.14 7.00 7.00 6.99 6.93 6.93 6.92 6.91 6.90 6.89 6.87 6.86 6.74 6.74 6.73 6.72 6.72 6.71 6.71 6.66 6.65 6.64 6.64
4.85
4.16
3.67
1.56
0.01
PMP
N
N
F
7.05
3.08
4.07
2.08
2.04
2.04
2.05
3.00
f1 (ppm)

3q

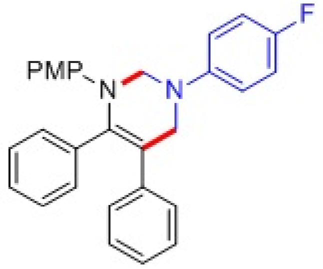
PMP
N
N
F

3q

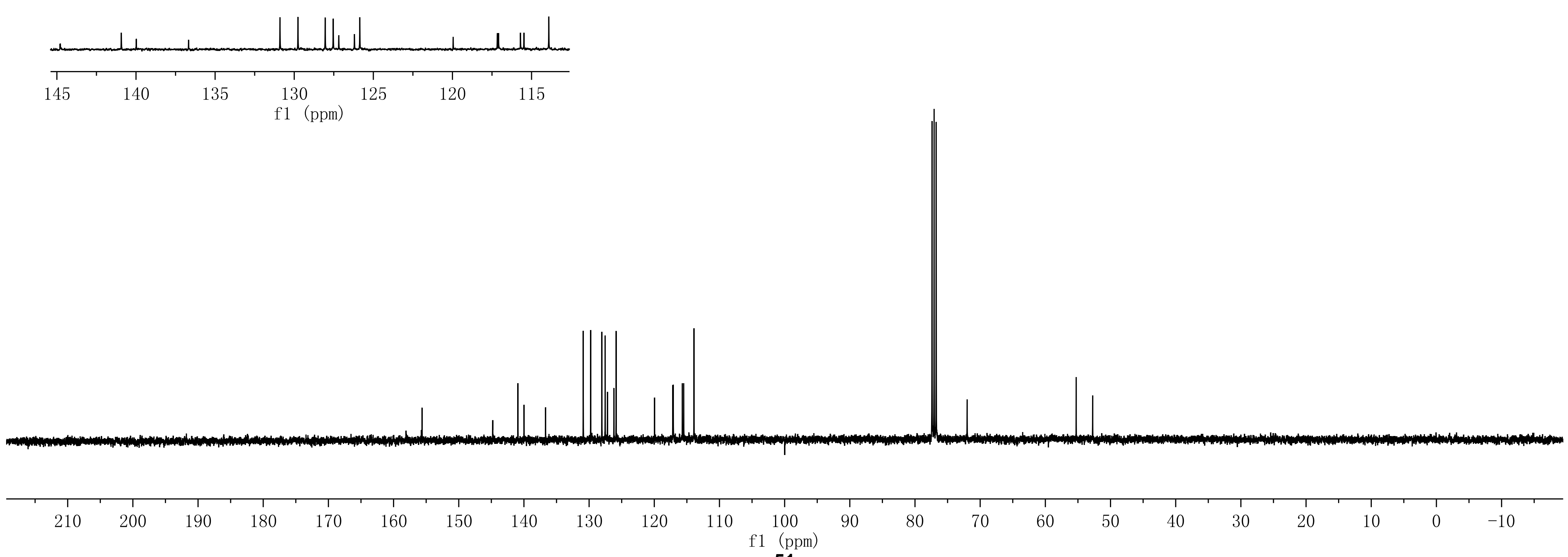
155.63
144.80
144.78
140.93
139.98
136.68
130.90
129.76
128.04
125.85
119.95
117.16
117.09
115.70
115.48
113.91
77.39
77.07
76.75
72.00
55.27
52.74
144.80
144.78
140.93
139.98
136.68
130.90
129.76
128.04
127.54
127.18
126.19
125.85
119.95
117.16
117.09
115.70
115.48
113.91
145 140 135 130 125 120 115
f1 (ppm)
210 200 190 180 170 160 150 140 130 120 110 100 90 80 70 60 50 40 30 20 10 0 -10
f1 (ppm)

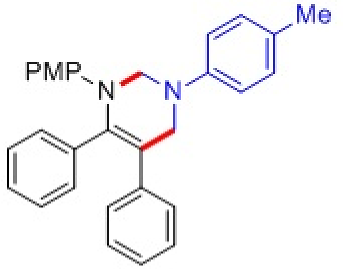
PMP
N
N
Me

3r

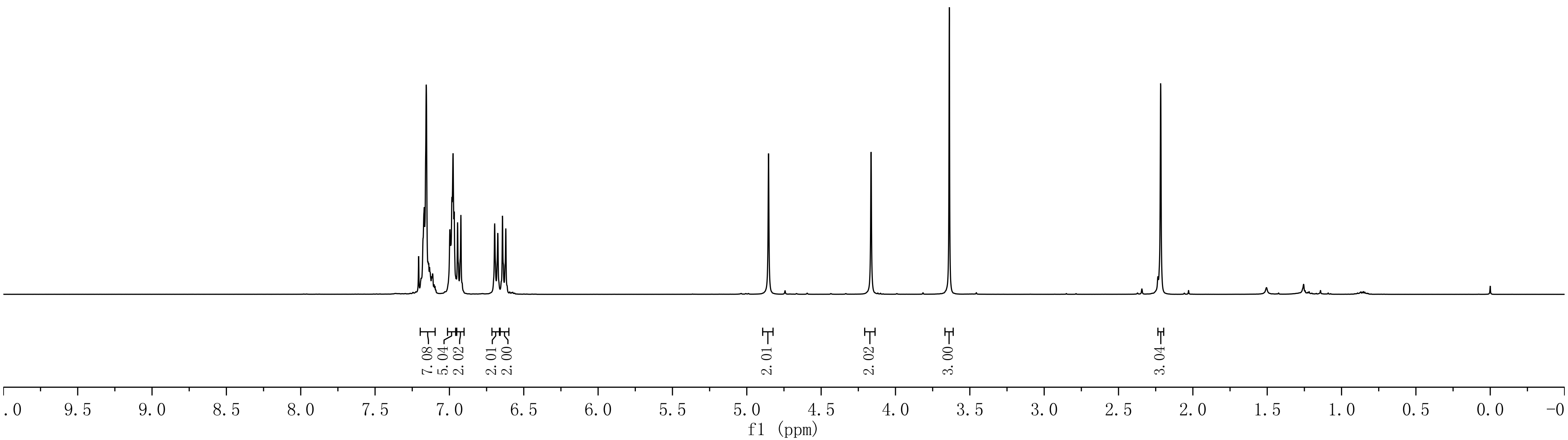
7.21
7.17
7.16
7.14
7.14
7.13
7.11
7.00
6.98
6.98
6.97
6.94
6.92
6.70
6.67
6.64
6.62
4.85
4.16
3.64
2.22
1.50
0.00
7.08
5.04
2.02
2.01
2.00
2.01
2.02
3.00
3.04
.0
9.5
9.0
8.5
8.0
7.5
7.0
6.5
6.0
5.5
5.0
4.5
4.0
3.5
3.0
2.5
2.0
1.5
1.0
0.5
0.0
-0
f1 (ppm)

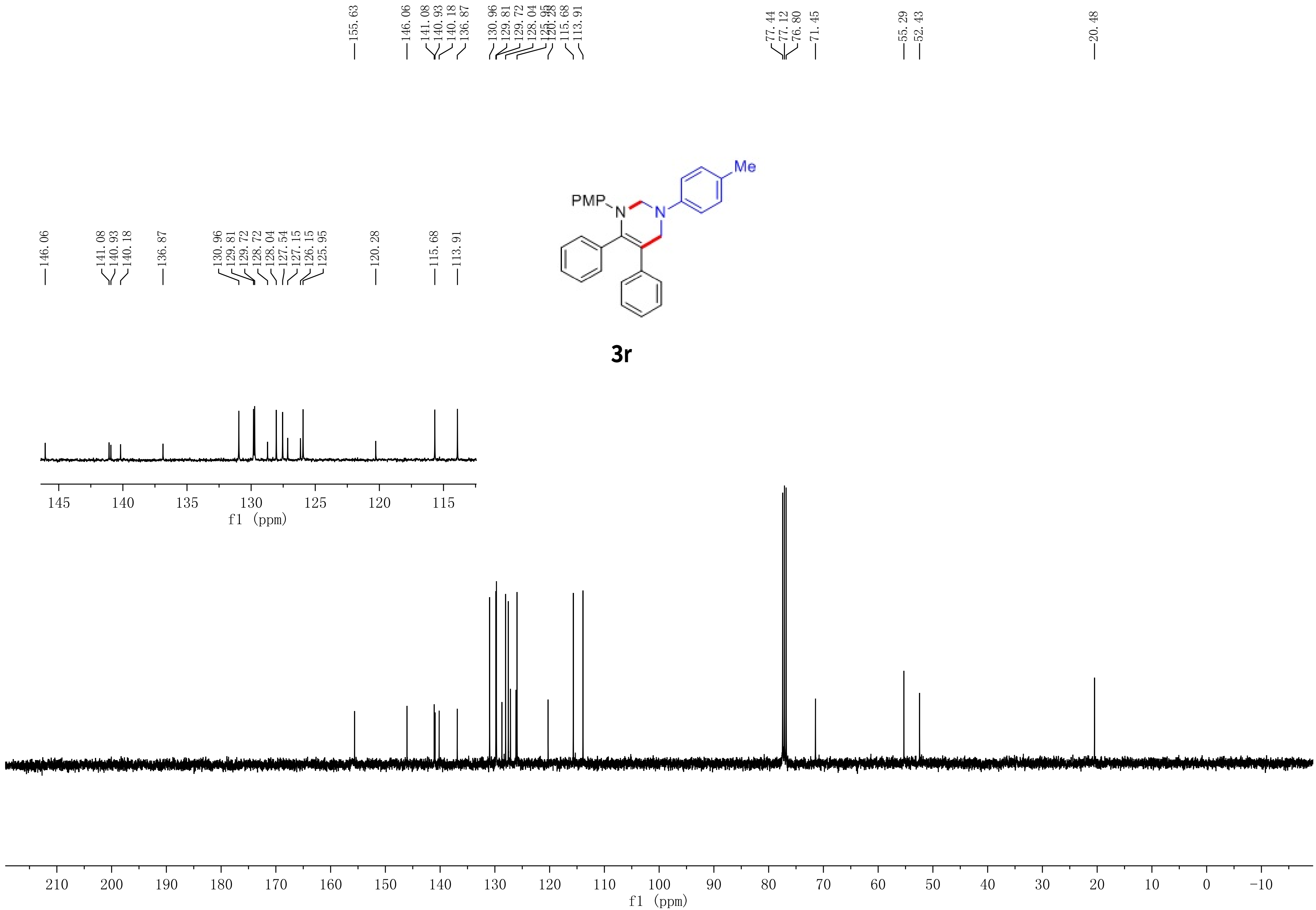
155.63
146.06
141.08
140.93
140.18
136.87
130.96
129.81
129.72
128.04
115.68
113.91
77.44
77.12
76.80
71.45
55.29
52.43
20.48
146.06
141.08
140.93
140.18
136.87
130.96
129.81
129.72
128.72
128.04
127.54
127.15
126.15
125.95
120.28
115.68
113.91
Me
PMP
N
N
3r
145
140
135
130
125
120
115
f1 (ppm)
210
200
190
180
170
160
150
140
130
120
110
100
90
80
70
60
50
40
30
20
10
0
-10
f1 (ppm)

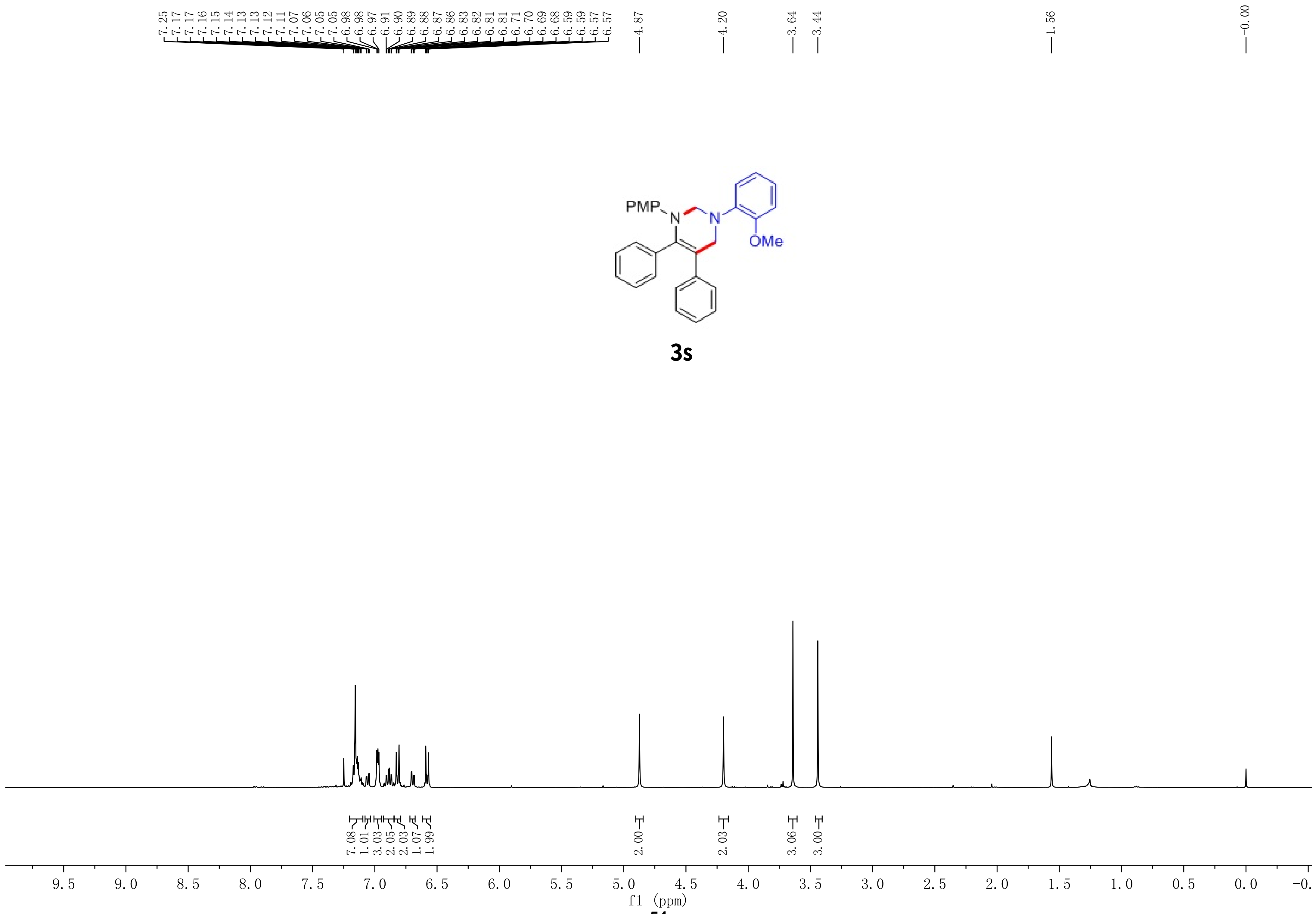
7.25 7.17 7.17 7.16 7.15 7.14 7.13 7.13 7.12 7.11 7.07 7.06 7.05 7.05 6.98 6.98 6.97 6.91 6.90 6.89 6.88 6.87 6.86 6.83 6.82 6.81 6.81 6.71 6.70 6.69 6.68 6.59 6.59 6.57 6.57
4.87
4.20
3.64
3.44
1.56
-0.00
PMP
N
N
OMe
3s
7.08 1.01 3.03 2.05 2.03 1.07 1.99
2.00
2.03
3.06
3.00
9.5 9.0 8.5 8.0 7.5 7.0 6.5 6.0 5.5 5.0 4.5 4.0 3.5 3.0 2.5 2.0 1.5 1.0 0.5 0.0 -0.
f1 (ppm)

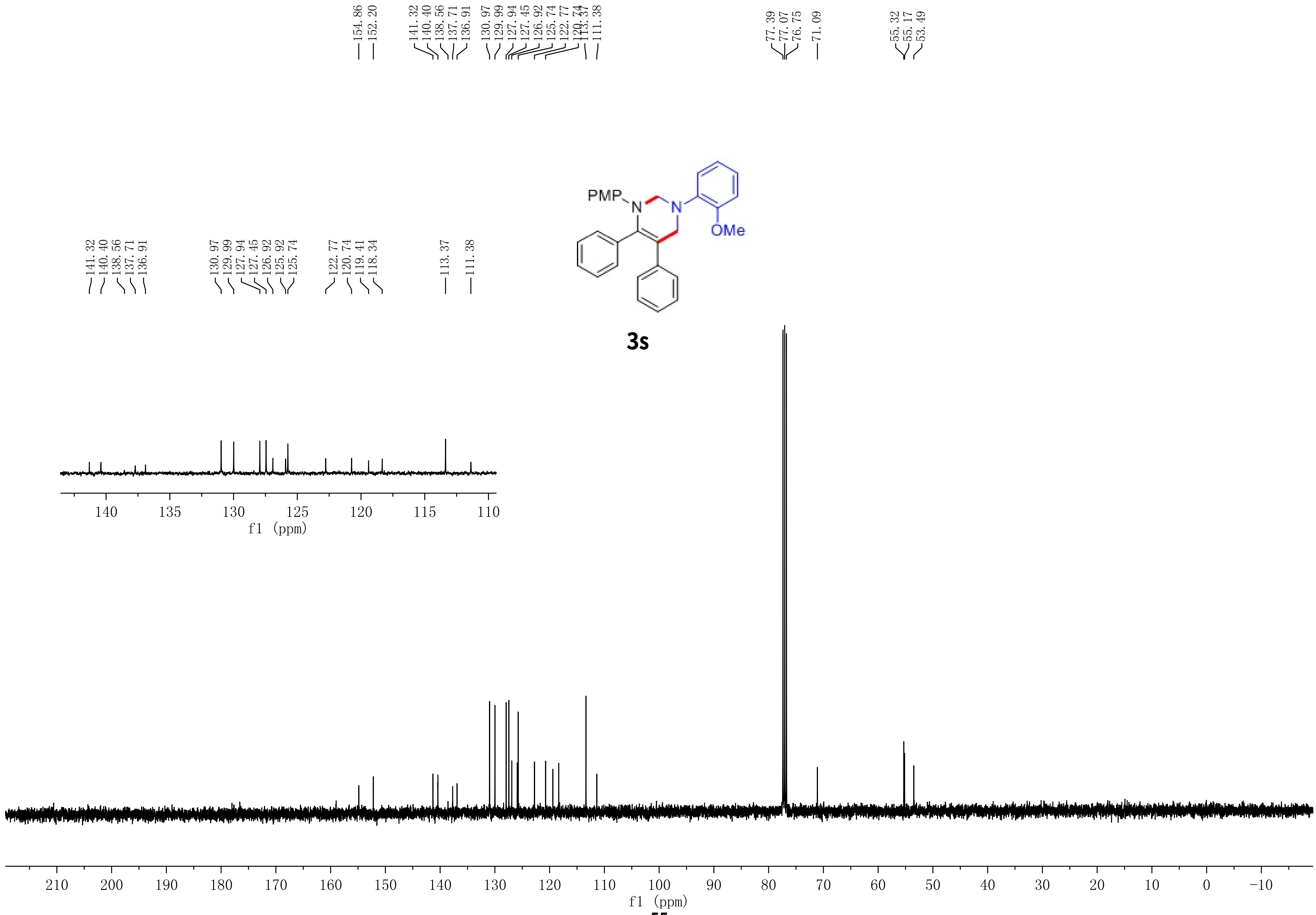
154.86
152.20
141.32
140.40
138.56
137.71
136.91
130.97
129.99
127.94
127.45
126.92
125.74
122.77
120.74
113.37
111.38
77.39
77.07
76.75
71.09
55.32
55.17
53.49
PMP
N
N
OMe
3s
141.32
140.40
138.56
137.71
136.91
130.97
129.99
127.94
127.45
126.92
125.92
125.74
122.77
120.74
119.41
118.34
113.37
111.38
140 135 130 125 120 115 110
f1 (ppm)
210 200 190 180 170 160 150 140 130 120 110 100 90 80 70 60 50 40 30 20 10 0 -10
f1 (ppm)

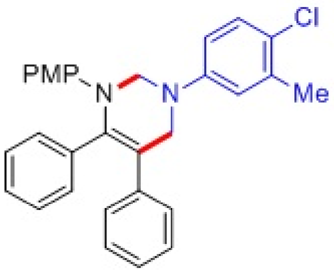
PMP
N
N
Cl
Me

3t

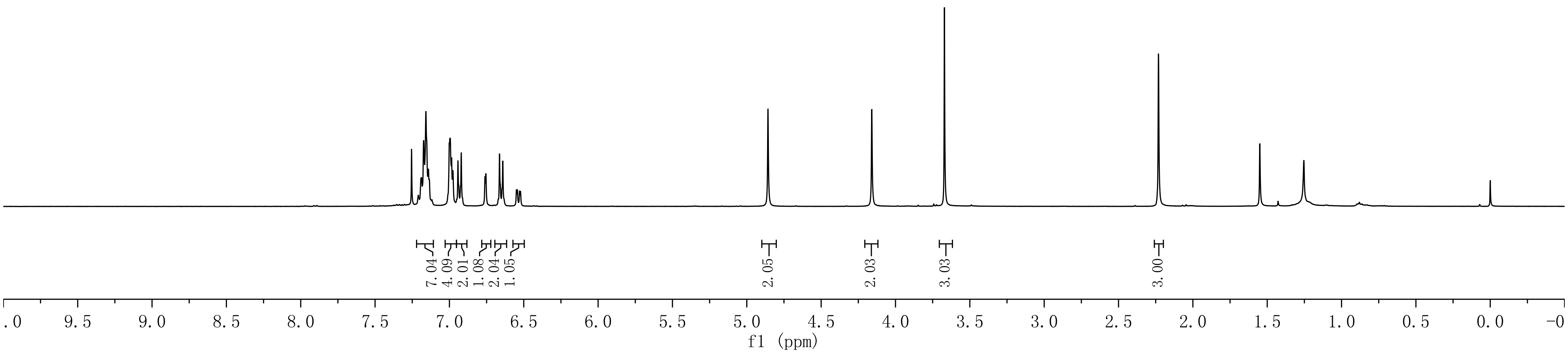
7.25 7.21 7.19 7.19 7.17 7.16 7.15 7.14 7.14 7.12 7.00 6.99 6.99 6.98 6.94 6.92 6.76 6.75 6.66 6.64 6.55 6.54 6.53 6.52
4.86
4.16
3.67
2.23
1.55
1.25
0.00
7.04 4.09 2.01 1.08 2.04 1.05
2.05
2.03
3.03
3.00
f1 (ppm)

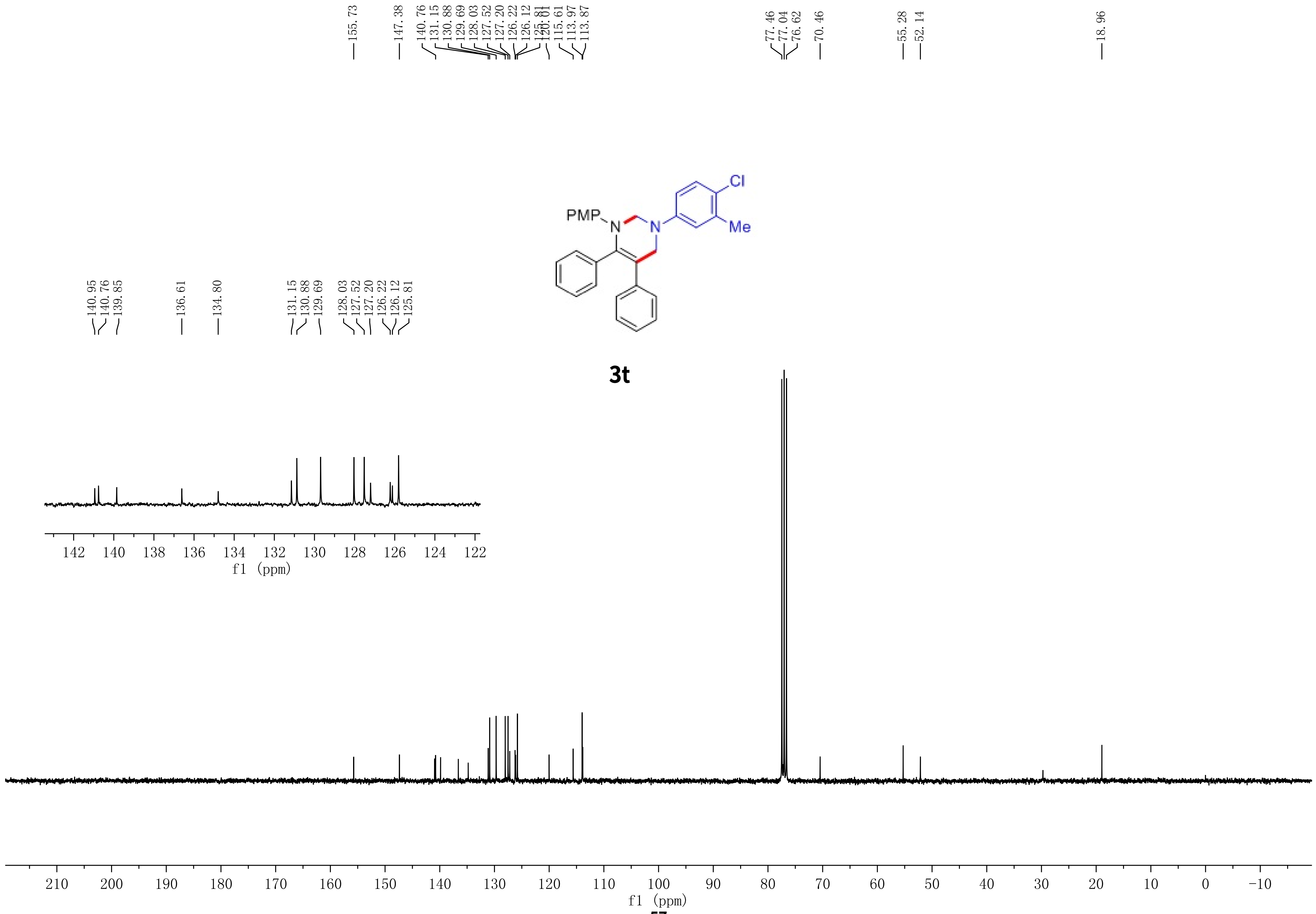
155.73
147.38
140.76
131.15
130.88
129.69
128.03
127.52
127.20
126.22
126.12
125.81
120.01
115.61
113.97
113.87
77.46
77.04
76.62
70.46
55.28
52.14
18.96
140.95
140.76
139.85
136.61
134.80
131.15
130.88
129.69
128.03
127.52
127.20
126.22
126.12
125.81
PMP
N
N
Cl
Me
3t
f1 (ppm)
f1 (ppm)

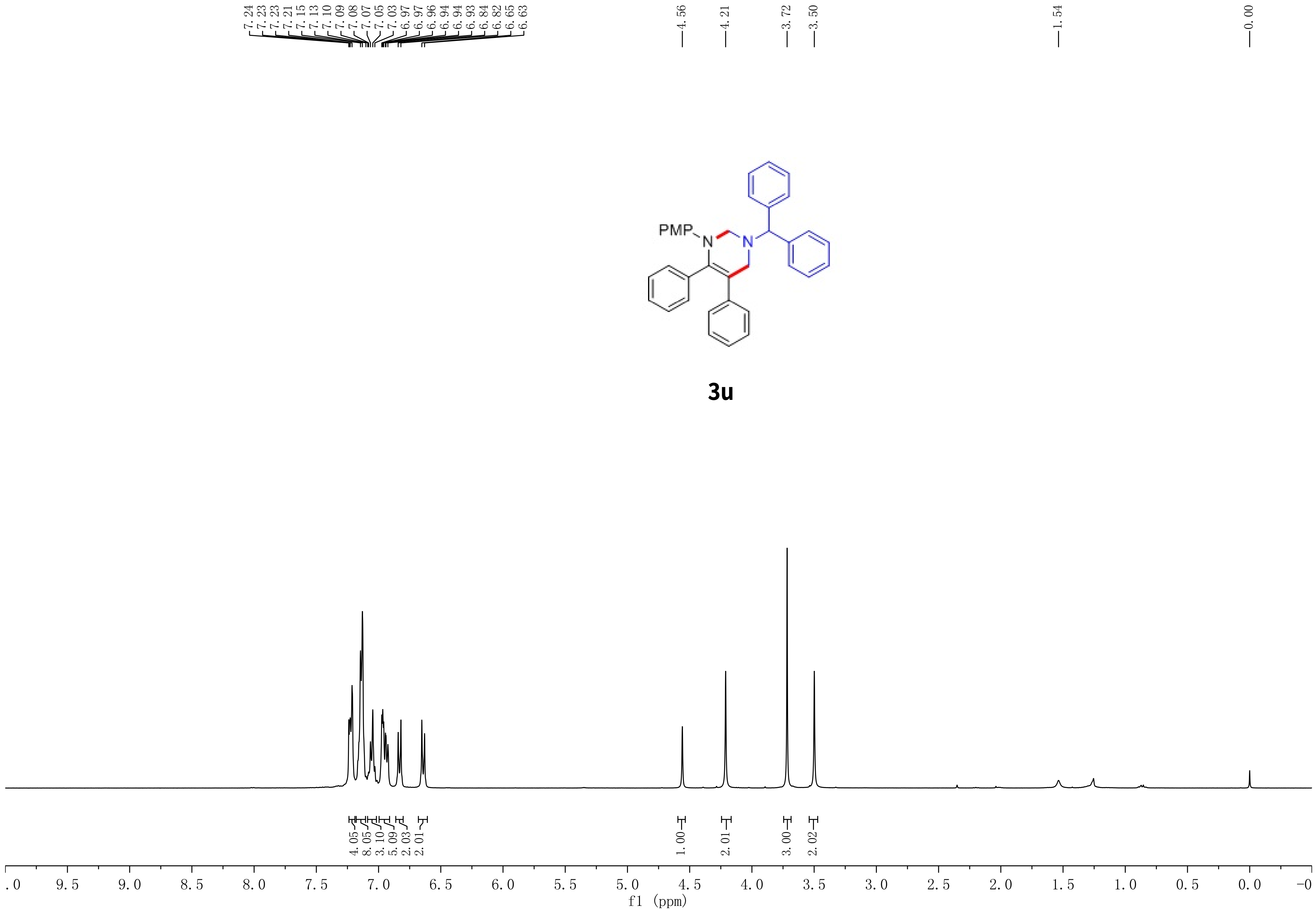
7.24 7.23 7.23 7.21 7.15 7.13 7.10 7.09 7.08 7.07 7.05 7.03 6.97 6.97 6.96 6.94 6.93 6.84 6.82 6.65 6.63
4.56
4.21
3.72
3.50
1.54
0.00
PMP
N
N
4.05 8.05 3.10 5.09 2.03 2.01
1.00
2.01
3.00
2.02
f1 (ppm)

3u

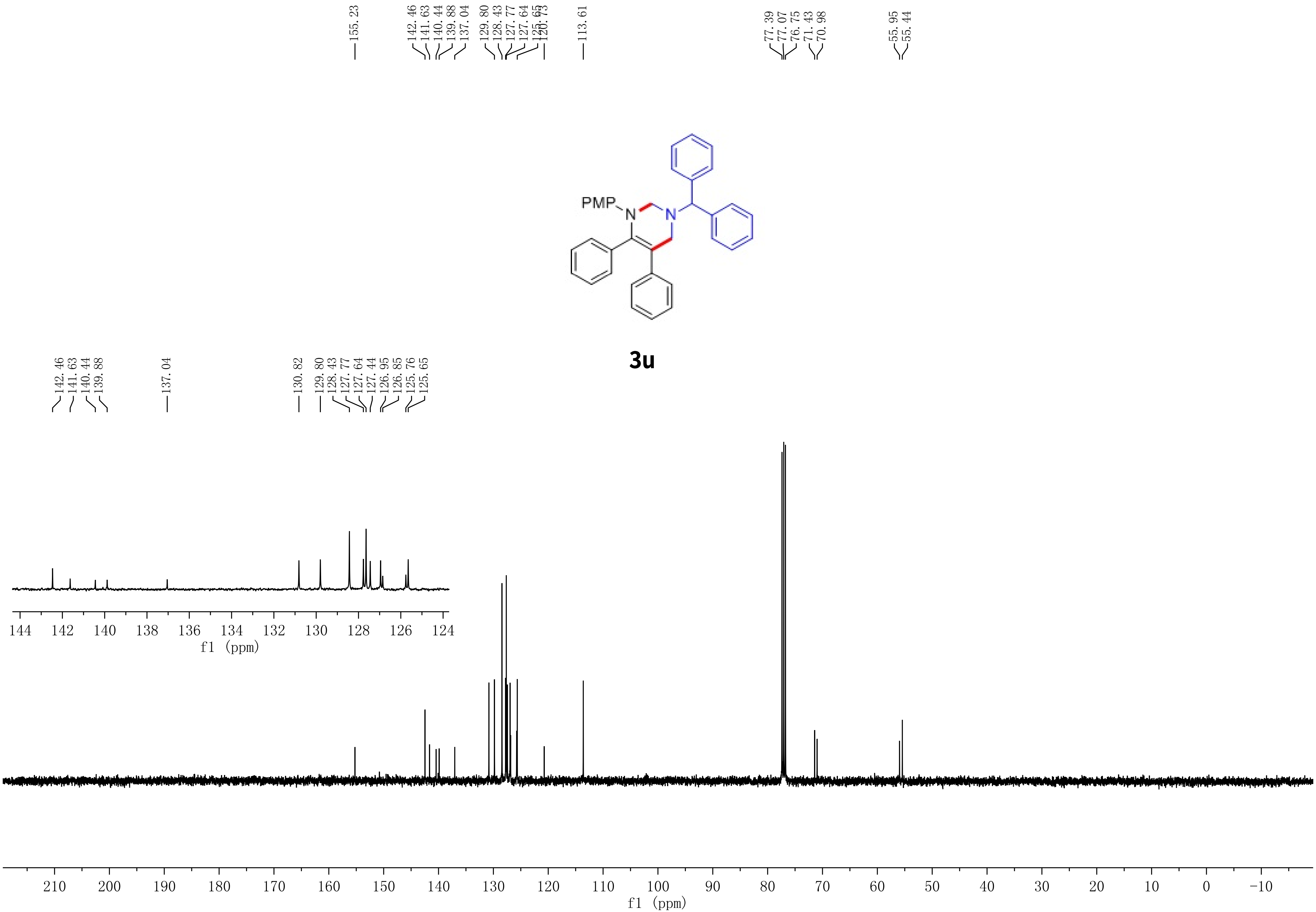
155.23
142.46
141.63
140.44
139.88
137.04
129.80
128.43
127.77
127.64
120.73
113.61
77.39
77.07
76.75
71.43
70.98
55.95
55.44
PMP
N
N
130.82
127.44
126.95
126.85
125.76
125.65
f1 (ppm)
f1 (ppm)

3u

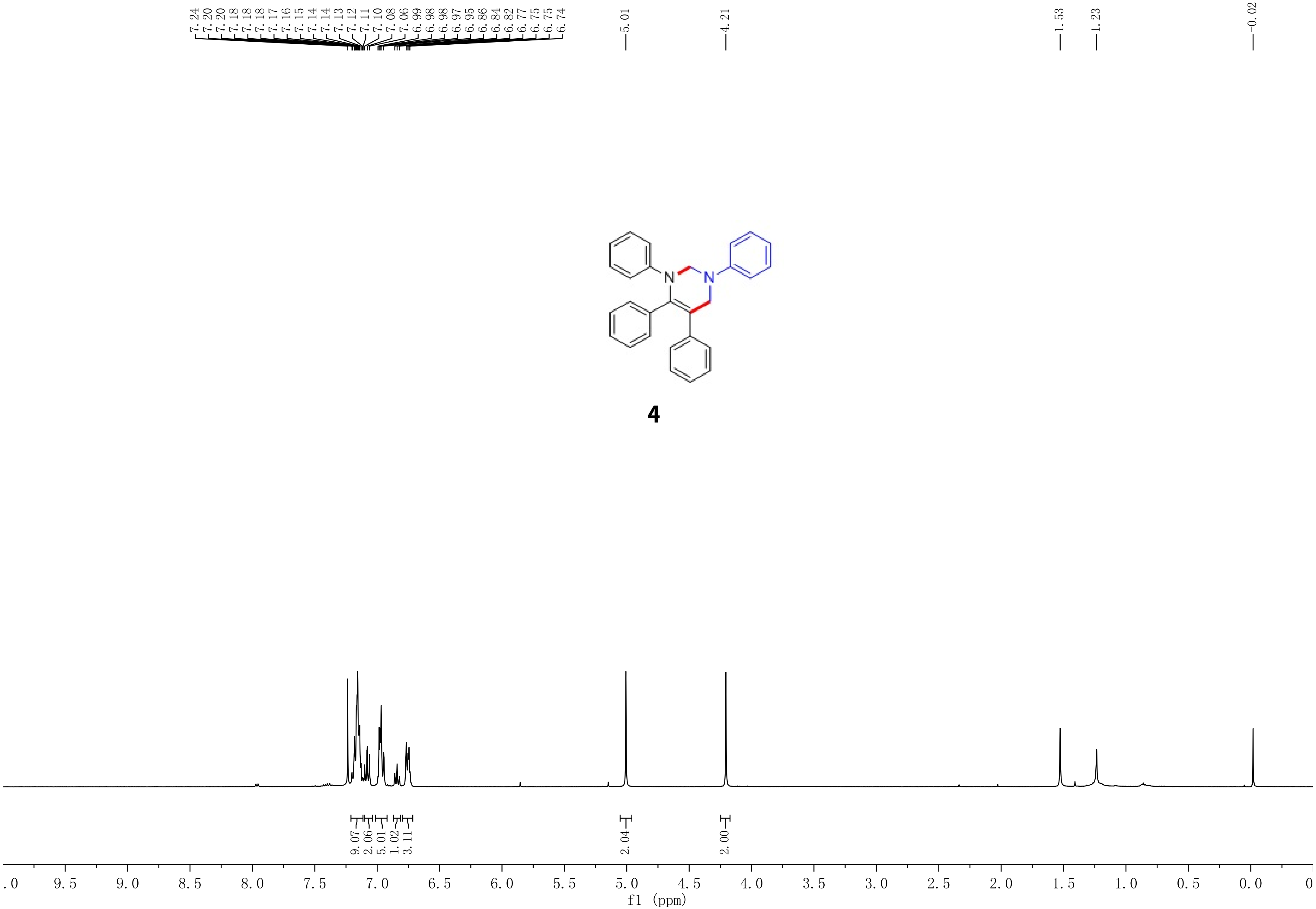
7.24
7.20
7.20
7.18
7.18
7.18
7.17
7.16
7.15
7.14
7.14
7.13
7.12
7.11
7.10
7.08
7.06
6.99
6.98
6.98
6.97
6.95
6.86
6.84
6.82
6.77
6.75
6.75
6.74
5.01
4.21
1.53
1.23
-0.02
N
N
4
9.07
2.06
5.01
1.02
3.11
2.04
2.00
f1 (ppm)

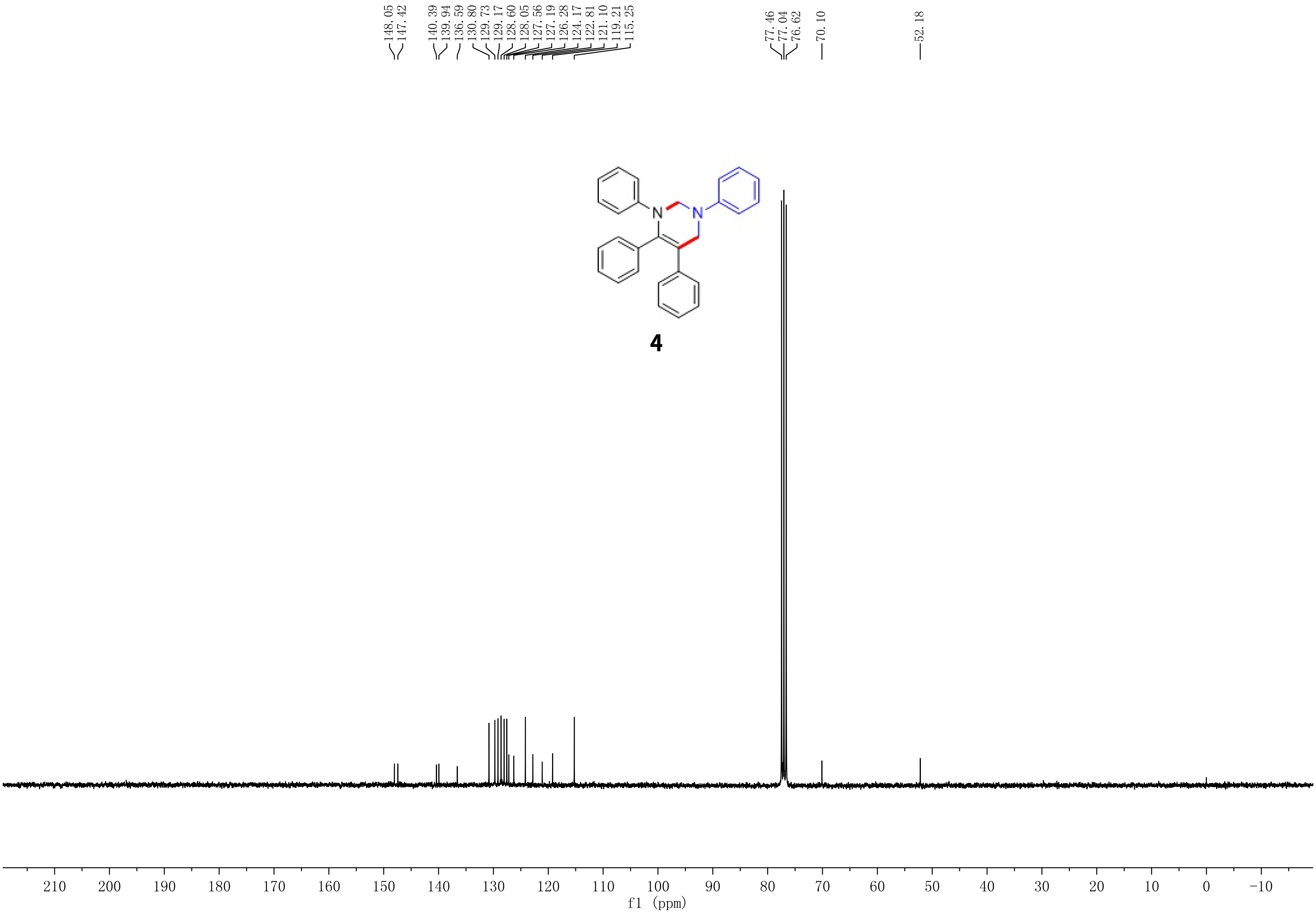
148.05
147.42
140.39
139.94
136.59
130.80
129.73
129.17
128.60
128.05
127.56
127.19
126.28
124.17
122.81
121.10
119.21
115.25
77.46
77.04
76.62
70.10
52.18
N
N
4
210 200 190 180 170 160 150 140 130 120 110 100 90 80 70 60 50 40 30 20 10 0 -10
f1 (ppm)

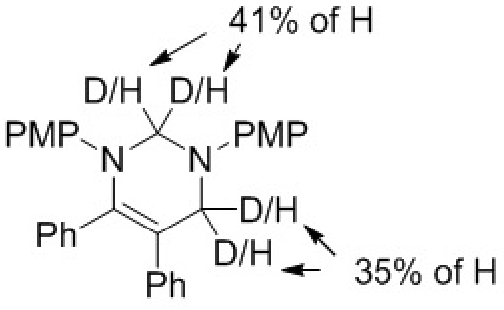
41% of H
D/H
D/H
PMP
N
N
PMP
D/H
D/H
Ph
Ph
35% of H

D-3a

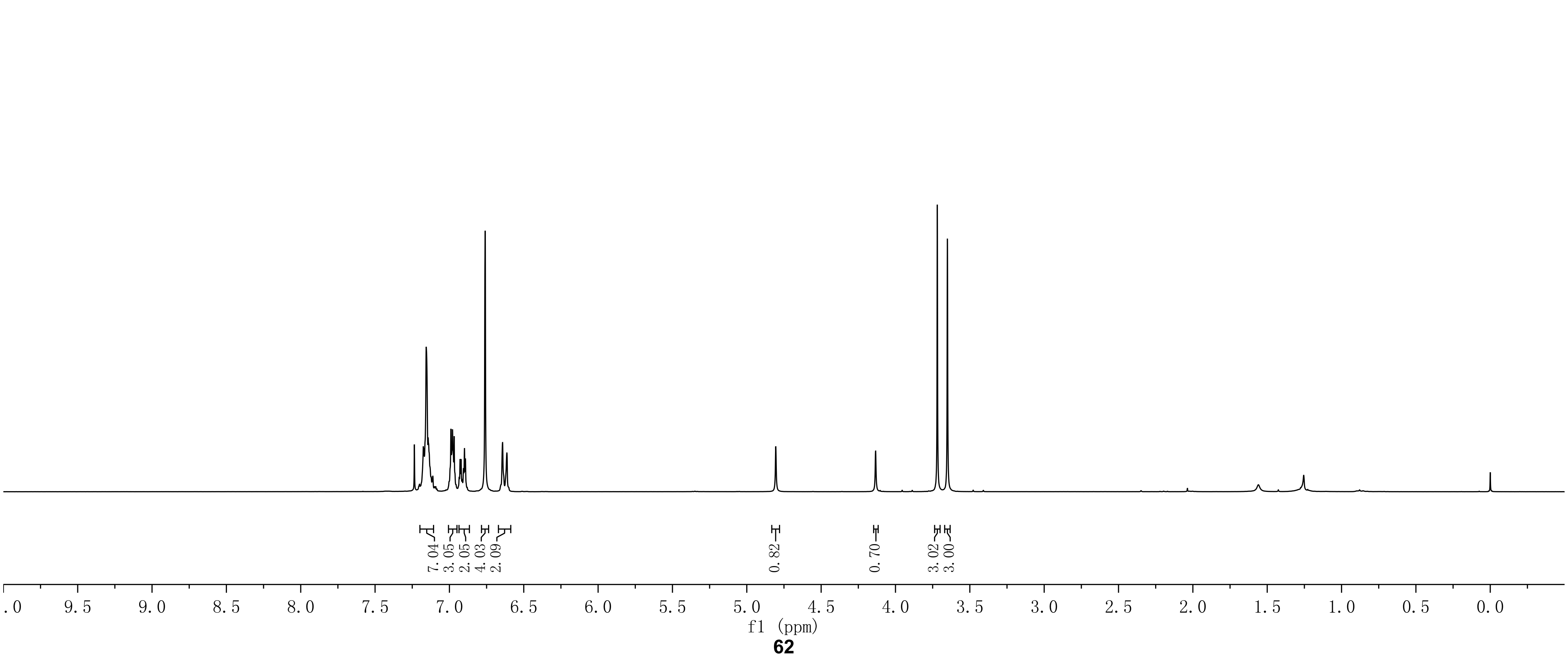
7.24
7.18
7.17
7.16
7.14
7.12
7.11
6.99
6.98
6.97
6.93
6.93
6.92
6.91
6.91
6.90
6.89
6.76
6.64
6.61
4.81
4.13
3.72
3.65
1.56
-0.00
7.04
3.05
2.05
4.03
2.09
0.82
0.70
3.02
3.00
9.5
9.0
8.5
8.0
7.5
7.0
6.5
6.0
5.5
5.0
4.5
4.0
3.5
3.0
2.5
2.0
1.5
1.0
0.5
0.0
f1 (ppm)

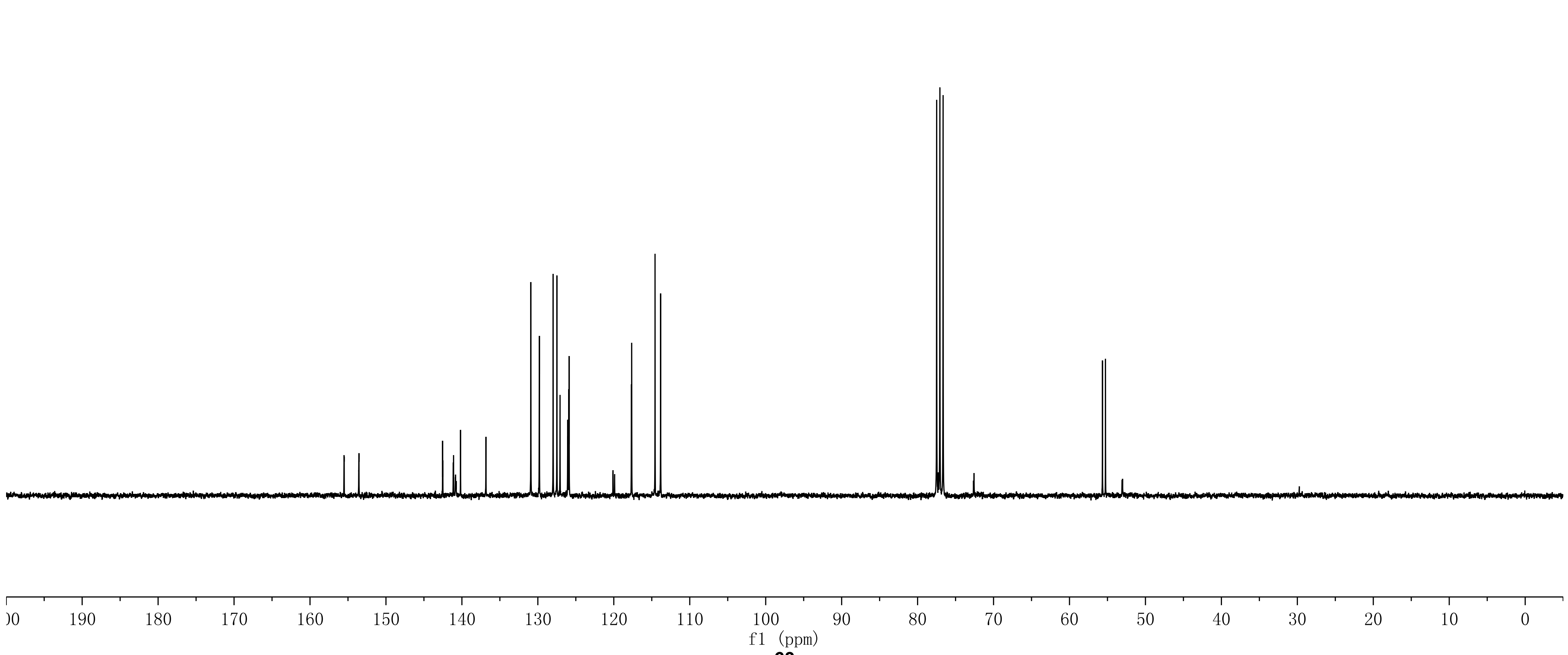
155.52
153.55
142.54
142.51
141.14
141.10
140.87
140.83
140.76
140.18
136.84
130.92
129.80
127.99
127.49
125.88
120.10
119.90
117.69
117.64
114.57
113.83
77.49
77.07
76.64
72.64
72.58
55.66
55.26
53.08
53.01
190 180 170 160 150 140 130 120 110 100 90 80 70 60 50 40 30 20 10 0
f1 (ppm)

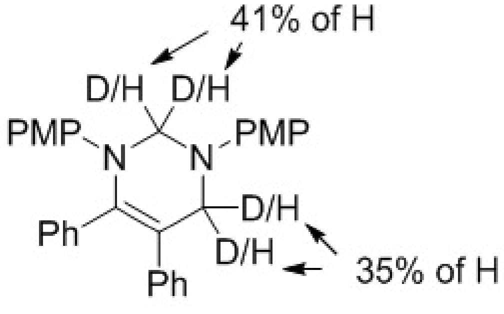
41% of H
D/H
D/H
PMP
N
N
PMP
D/H
D/H
Ph
Ph
35% of H

D-3a
